# Use of Natural Products in Leishmaniasis Chemotherapy: An Overview

**DOI:** 10.3389/fchem.2020.579891

**Published:** 2020-11-23

**Authors:** Luiza F. O. Gervazoni, Gabrielle B. Barcellos, Taiana Ferreira-Paes, Elmo E. Almeida-Amaral

**Affiliations:** Laboratório de Bioquímica de Tripanosomatideos, Instituto Oswaldo Cruz, Fundação Oswaldo Cruz, Rio de Janeiro, Brazil

**Keywords:** natural product, Leishmaniasis, chemotherapy, *in vivo*, *in vitro*, intracellular amastigotes

## Abstract

Leishmaniasis is an infectious parasitic disease that is caused by protozoa of the genus *Leishmania*, a member of the Trypanosomatidae family. Leishmaniasis is classified by the World Health Organization as a neglected tropical disease that is responsible for millions of deaths worldwide. Although there are many possible treatments for leishmaniasis, these treatments remain mostly ineffective, expensive, and long treatment, as well as causing side effects and leading to the development of resistance. For novel and effective treatments to combat leishmaniasis, many research groups have sought to utilize natural products. In addition to exhibiting potential as therapeutic compounds, natural products may also contribute to the development of new drugs based on their chemical structures. This review presents the most promising natural products, including crude extracts and isolated compounds, employed against *Leishmania* spp.

## Introduction

Caused by protozoa of the genus *Leishmania*, which is a member of the Trypanosomatidae family, leishmaniasis is an infectious parasitic disease. This disease has a wide variety of clinical manifestations, ranging from the cutaneous form to the visceral form. Visceral leishmaniasis, the form that can cause death, affects the organs and viscera of mammalian hosts; conversely, cutaneous leishmaniasis, which can be divided into different manifestations, affects the skin and mucous membranes of mammalian hosts. Classified as a neglected disease by the World Health Organization (WHO, 2016), leishmaniasis affects over 300 million people across all continents.

The current treatment for leishmaniasis is based on pentavalent antimonials, which are drugs that were developed over decades (Vianna, 1912; Burza et al., 2018) with a long-established administration profile in the hospital environment. These drugs are becoming increasingly ineffective due to resistance. Amphotericin B emerged as an alternative treatment; however, its long-standing treatment and dose-dependent side effects led to the development of a liposomal formulation to significantly reduce the side effects and duration of treatment; nevertheless, this formulation is expensive. Paromomycin is already registered in India, but the effectiveness of this treatment has not been determined to date in Africa. As the most promising treatment discovered in recent decades and the first oral drug for leishmaniasis, miltefosine is registered in India and a small number of other countries and has recently been registered by the FDA (IMPAVIDO) for the treatment of visceral and cutaneous leishmaniasis. Miltefosine is effective but expensive and teratogenic (DNDi, 2016).

Although there are many possibilities for leishmaniasis treatment, these treatments remain mostly ineffective, expensive and old, as well as causing side effects and leading to the development of resistance. For novel and effective treatments to combat leishmaniasis, many research groups have investigated natural products.

Natural products are secondary metabolites present in the roots, stalks, leaves, fruits, seeds, vegetables, and other parts of plants with a wide structural variety that mediates interactions between plants and their environment. These metabolites are usually observed around the world in diets as main foods or teas, spices, and sauces. A considerable number of metabolites have anti-protozoal activity (Winkel, 2006; Schmidt et al., 2012a).

Natural products are known in pharmacology for having potential applications as therapeutic drugs, which have been described since ancient times, in addition to contributing to the discovery and development of new drugs based on the chemical structures of these products with specific modifications (Viegas et al., 2006; Ioset, 2008).

In this review, we present the most promising crude extracts and isolated compounds derived from the four major plant metabolic pathways; these products were recently studied to determine their effectiveness as chemotherapy agents for treating leishmaniasis.

All crude extracts and compounds that have defined IC_50_ values are represented in tables at the end of each section.

## Materials and Methods

This review aims to update and summarize information concerning the early drug discovery process based on crude extracts, fractions, and isolated compounds obtained from natural products, specifically herbal-derived compounds, to treat leishmaniasis. The keywords employed in this study included leishmaniasis, natural products, chemotherapy, *in vivo*, *in vitro*, and intracellular amastigote using the current databases: PubMed, Web of Science, Science Direct, and Google Scholar. Our search covered English-language articles published in international scientific journals, indexed over the period 2000-2020. The choice criteria were articles that investigated the leishmanicidal activity of natural products against promastigote, axenic amastigote, and intracellular amastigote forms, the mechanism of action and/or the use of advanced techniques to search for alternative treatments for leishmaniasis. Selected articles describing the use of novel natural products with leishmanicidal activity against promastigotes of *Leishmania* spp. were considered. All crude extracts and compounds that have defined IC_50_ values are represented in Tables 1–**7**.

**Table 1:** Leishmanicidal activities of crude extracts and fractions.

**Class**	**Plant**	**Part**	**Extract/fraction**	***Leishmania* species**	**Assay**	**Values**	**References**
Crude extracts and fractions	*Jurinea dolomiaea*	Roots	Methanol extract	*Leishmania tropica*	*In vitro*	**Promastigotes:** IC_50_: 10.9 μg/mL	Shah et al., 2014
					*In vivo* toxicity assay	**Brine shrimp test:** CC_50_: 733.0 μg/mL	
			n-Hexane fraction		*In vitro*	**Promastigotes:** IC_50_: 7.2 μg/mL	
					*In vivo* toxicity assay	**Brine shrimp test:** CC_50_: 982.5 μg/mL	
			Chloroform fraction		*In vitro*	**Promastigotes:** IC_50_: 47.7 μg/mL	
					*In vivo* toxicity assay	**Brine shrimp test:** CC_50_: 834.5 μg/mL	
			Ethyl acetate fraction		*In vitro*	**Promastigotes:** IC_50_: 5.3 μg/mL	
					*In vivo* toxicity assay	**Brine shrimp test:** CC_50_: 569.5 μg/mL	
			n-Butanol fraction		*In vitro*	**Promastigotes:** IC_50_: 21.8 μg/mL	
					*In vivo* toxicity assay	**Brine shrimp test:** CC_50_: 958.3 μg/mL	
			Water fraction		*In vitro*	**Promastigotes:** IC_50_: 6.0 μg/mL	
					*In vivo* toxicity assay	**Brine shrimp test:** CC_50_: 1593.0 μg/mL	
Crude extracts and fractions	*Asparagus gracilis*	Aerial	Methanol extract	*Leishmania tropica*	*In vitro*	**Promastigotes:** IC_50_: 33.9 μg/mL	Shah et al., 2014
					*In vivo* toxicity assay	**Brine shrimp test:** CC_50_: 321.5 μg/mL	
			n-Hexane fraction		*In vitro*	**Promastigotes:** IC_50_: 36.6 μg/mL	
					*In vivo* toxicity assay	**Brine shrimp test:** CC_50_: 280.6 μg/mL	
			Chloroform fraction		*In vitro*	**Promastigotes:** IC_50_: 28.3 μg/mL	
					*In vivo* toxicity assay	**Brine shrimp test:** CC_50_: 383.5 μg/mL	
			Ethyl acetate fraction		*In vitro*	**Promastigotes:** IC_50_: 13.5 μg/mL	
					*In vivo* toxicity assay	**Brine shrimp test:** CC_50_: 211.9 μg/mL	
			n-Butanol fraction		*In vitro*	**Promastigotes:** IC_50_: 18.9 μg/mL	
					*In vivo* toxicity assay	**Brine shrimp test:** CC_50_: 588.6 μg/mL	
			Water fraction		*In vitro*	**Promastigotes:** IC_50_: 12.6 μg/mL	
					*In vivo* toxicity assay	**Brine shrimp test:** CC_50_: 460.0 μg/mL	
Crude extracts and fractions	*Sida cordata*	Whole plant	Methanol extract	*Leishmania tropica*	*In vitro*	**Promastigotes:** IC_50_: 41.8 μg/mL	Shah et al., 2014
					*In vivo* toxicity assay	**Brine shrimp test:** CC_50_: 125.7 μg/mL	
			n-Hexane fraction		*In vitro*	**Promastigotes:** IC_50_: 9.2 μg/mL	
					*In vivo* toxicity assay	**Brine shrimp test:** CC_50_: 879.5 μg/mL	
			Chloroform fraction		*In vitro*	**Promastigotes:** IC_50_: 125.5 μg/mL	
					*In vivo* toxicity assay	**Brine shrimp test:** CC_50_: 802.8 μg/mL	
			Ethyl acetate fraction		*In vitro*	**Promastigotes:** IC_50_: 56.8 μg/mL	
					*In vivo* toxicity assay	**Brine shrimp test:** CC_50_: 309.9 μg/mL	
			n-Butanol fraction		*In vitro*	**Promastigotes:** IC_50_: 228.5 μg/mL	
					*In vivo* toxicity assay	**Brine shrimp test:** CC_50_: 882.4 μg/mL	
			Water fraction		*In vitro*	**Promastigotes:** IC_50_: 259.1 μg/mL	
					*In vivo* toxicity assay	**Brine shrimp test:** CC_50_: 211.9 μg/mL	
Crude extracts and fractions	*Stellaria media*	Whole plant	Methanol extract	*Leishmania tropica*	*In vitro*	**Promastigotes:** IC_50_: 185.9 μg/mL	Shah et al., 2014
					*In vivo* toxicity assay	**Brine shrimp test:** CC_50_: 436.7 μg/mL	
			n-Hexane fraction		*In vitro*	**Promastigotes:** IC_50_: 170.4 μg/mL	
					*In vivo* toxicity assay	**Brine shrimp test:** CC_50_: 542.5 μg/mL	
			Chloroform fraction		*In vitro*	**Promastigotes:** IC_50_: 155.5 μg/mL	
					*In vivo* toxicity assay	**Brine shrimp test:** CC_50_: 600.0 μg/mL	
			Ethyl acetate fraction		*In vitro*	**Promastigotes:** IC_50_: 36.4 μg/mL	
					*In vivo* toxicity assay	**Brine shrimp test:** CC_50_: 789.3 μg/mL	
			n-Butanol fraction		*In vitro*	**Promastigotes:** IC_50_: 49.5 μg/mL	
					*In vivo* toxicity assay	**Brine shrimp test:** CC_50_: 760.2 μg/mL	
			Water fraction		*In vitro*	**Promastigotes:** IC_50_: 184.8 μg/mL	
					*In vivo* toxicity assay	**Brine shrimp test:** CC_50_: 660.7 μg/mL	
Crude extracts and fractions	*Bowdichia virgiloides* Kunth.	Leaves	Ethanolic	*L. amazonesis*	*In vitro*	NA	Ribeiro et al., 2014
			Hexanic			NA	
	*Campomanesia lineatifolia* Ruiz & Pav	Leaves	Ethanolic			**Promastigotes:** IC_50_: 103.3 μg/mL	
			Buthanolic fraction			**Promastigotes:** IC_50_: 62.3 μg/mL	
			Dichloromethane fraction			NA	
			Ethyl acetate fraction			**Promastigotes:** IC_50_: 96.1 μg/mL	
			Hexanic fraction			**Promastigotes:** IC_50_: 147.7 μg/mL	
	*Cecropia pachystachya* Trécul	Leaves	Ethanolic			NA	
			Hexanic			NA	
	*Chrysobalanus icaco* L.	Leaves	Ethanolic			**Promastigotes:** IC_50_: 61.5 μg/mL	
			Hexanic			**Promastigotes:** IC_50_: 62.3 μg/mL	
			Buthanolic fraction			**Promastigotes:** IC_50_: 61.2 μg/mL	
			Dichloromethane fraction			**Promastigotes:** IC_50_: 91.6 μg/mL	
			Ethyl acetate fraction			**Promastigotes:** IC_50_: 77.3 μg/mL	
			Hexanic fraction			**Promastigotes:** IC_50_: 130.2 μg/mL	
Crude extracts and fractions	*Diospyros hispida* D.C.	Leaves	Ethanolic	*L. amazonesis*	*In vitro*	NA	Ribeiro et al., 2014
			Hexanic			NA	
	*Dipteryx alata* Vog.	Leaves	Ethanolic			**Promastigotes:** IC_50_: 51.5 μg/mL	
			Hexanic			**Promastigotes:** IC_50_: 0.08 μg/mL Intracellular amastigotes: 0.08 μg/mL	
	*Syzygium cumini* Lam.	Leaves	Hexanic			**Promastigotes:** IC_50_: 31.6 μg/mL	
	*Eugenia uniflora* L.	Leaves	Hexanic			NA	
	*Hymenaea courbaril* L.	Leaves	Ethanolic			**Promastigotes:** IC_50_: 44.1 μg/mL	
			Hexanic			**Promastigotes:** IC_50_: 35.8 μg/mL	
	*Hymenaea stignocarpa* Mart. ex. Hayne	Leaves	Ethanolic			**Promastigotes:** IC_50_: 4.7 μg/mL	
			Hexanic			**Promastigotes:** IC_50_: 199.4 μg/mL	
	*Jacaranda caroba* Vell.	Leaves	Ethanolic			**Promastigotes:** IC_50_: 13.2 μg/mL	
			Hexanic			NA	
Crude extracts and fractions	*Jacaranda cuspidifolia* Mart.	Roots Leaves	Ethanolic	*L. amazonesis*	*In vitro*	NA	Ribeiro et al., 2014
			Ethanolic			**Promastigotes:** IC_50_: 10.96 μg/mL **Intracellular amastigotes:** 5 μg/mL	
			Hexanic			NA	
			Chloroformic fraction			**Promastigotes:** IC_50_: 7.4 μg/mL **Intracellular amastigotes:** 5 μg/mL	
	*Jacaranda ulei* Bureau & K. Schum.	Stem bark Leaves	Ethanolic			NA	
			Ethanolic			NA	
			Hexanic			NA	
	*Vernonia phosphorea* Vell.	Stem bark Roots Leaves	Hexanic			**Promastigotes:** IC_50_: 160.9 μg/mL	
			Ethanolic			**Promastigotes:** IC_50_: 92.7 μg/mL	
			Ethanolic			NA	
			Hexanic			NA	
Crude extracts and fractions	*Physalis angulate* (EEPa)	Stem	Ethanolic extract	*L. amazonesis*	*In vitro*	**Promastigotes:** IC_50_: 5.4 μg/mL **Intracellular amastigotes:** IC_50_: 1.2 μg/mL	Nogueira et al., 2013
				*L. braziliensis*		**Promastigotes:** IC_50_: 4.5 μg/mL	
	*Tetradenia riparia* (TrEO)	Leaves	Essential oil	*L. amazonesis*	*In vitro*	**Promastigotes:** IC_50_: 0.03 μg/mL **Intracellular amastigotes:** IC_50_: 0.03 μg/mL	Demarchi et al., 2015

*NA, no activity (IC_50_ > 200 μg/mL)*.

## Crude Extracts and Fractions (Leaves, Roots, Seeds, Fruits, and Stalks)

It is well-known that crude extracts have been employed as medicinal drugs since the times of ancient civilizations. In those eras, the simple action of grinding the leaves of certain plants was considered to be medicine. With the growth of technology and knowledge, extracts from leaves, seeds, and other parts of the plant have been tested against several diseases, and some of these extracts have been highly successful (Viegas et al., 2006).

Four plants from different families, namely, *Asparagus gracilis* from the *Asparagaceae* family, *Stellaria media* from the *Caryophyllaceae* family, *Sida cordata* from the *Malvaceae* family and *Jurinea dolomiaea* (*J. dolomiaea*) from the *Asteraceae* family, were tested against a strain of *Leishmania tropica* isolated from a patient from Pakistan. All four plants were prepared as methanol extracts or n-hexane, chloroform, ethyl acetate, n-butanol and water fractions. The most potent methanol extract was from *J. dolomiaea*, which exhibited an IC_50_ value of 10.9 μg/mL, but the highest antileishmanial activity was obtained from the ethyl acetate fraction from *J. dolomiaea* with an IC_50_ value of 5.3 μg/mL. All of the extracts and fractions were not toxic, exhibiting IC_50_ values greater than 100 μg/mL and potent extracts with selectivity indices greater than 10 (Shah et al., 2014).

A series of 16 Brazilian medicinal plants were investigated *in vitro* to determine their efficacy against *L. amazonensis*. Among the 44 extracts and fractions, the most potent were the hexanic fraction of *Dipteryx alata* (*D. alata*) with an IC_50_ value of 0.08 μg/mL, the ethanolic fraction of *Hymenaea stignocarpa* with an IC_50_ value of 4.70 μg/mL, and both the chloroformic and ethanolic fractions of *Jacaranda cuspidifolia* (*J. cuspidifolia*), which exhibited IC_50_ values of 7.4 and 10.96 μg/mL, respectively (Ribeiro et al., 2014).

*Physalis angulata*, which is from the *Solanaceae* family, is a well-known medicinal plant (Mahalakshmi and Nidavani, 2014). For leishmaniasis, Nogueira et al. (2013) tested the ethanolic extract of this plant against two species of *Leishmania*. In an antipromastigote assay, EEPa (ethanolic extract of *Physalis angulata*) exhibited IC_50_ values of 5.35 and 4.50 g/mL for *Leishmania amazonensis* and *Leishmania braziliensis*, respectively. The antiamastigote assay using *L. amazonensis* demonstrated an IC_50_ value of 1.23 g/mL with a selectivity index of 5.

*Tetradenia riparia*, a plant from the *Lamiaceae* family, is commonly employed as a traditional medicine in Africa for infectious parasitic diseases, such as malaria, cryptococcosis, and candidiasis. Against an *L. amazonensis* promastigote, the essential oil of *Tetradenia riparia* (TrEO), which contains a mixture of terpenoids, exhibited an IC_50_ value of 0.03 μg/mL after 24 h. Cytotoxicity in human erythrocytes was tested, and at a concentration of 5 μg/mL, TrEO was determined not to be toxic (Demarchi et al., 2015). Alterations in promastigote morphology were observed. TrEO was able to induce cytoplasmic vacuolization, and membranous profiles and lipid vesicles appeared in the organelle along with membrane blebbing, nuclear fragmentation and chromatin condensation, which are events that are associated with autophagy. Against the intracellular amastigote form, TrEO showed an IC_50_ value of 0.03 μg/mL and a selectivity index of 5.6. TrEO was also capable of increasing the mRNA expression of iNOS in murine peritoneal macrophages; however, alterations in nitrite production were not observed.

Crude extracts and fraction presented in this section are summarized in Table 1.

## Metabolites of the Shikimate Pathway

### Lignans and Neolignans

Lignans and neolignans are metabolites that can be found in approximately 60 vascular plant families (Winkel, 2006). Lignans are dimeric phenylpropanoids, and neolignans are small molecules with two phenylpropanoid units. The diversity in this class consists of the distribution of aromatic rings and the nature of the propyl fragments (Rye and Barker, 2013). Several groups have chosen to investigate lignans and neolignans because they have properties favorable to drug development and for their anti-inflammatory and antioxidant activity, which may minimize the effects of the inflammatory response (Maia et al., 2020).

Notably, several studies have focused on the search for natural compounds with potential leishmanicidal activity directly using biological screening through phenotypic methods to assess the potential of lignans and neolignans against *Leishmania* species. The efficacy of a lignin found in garlic (*Allium sativum*) against *L. amazonensis* promastigotes was investigated. Dehydrodieuginol (DHDE), an ortho-biphenyl neolignan, showed an IC_50_ value of 42.2 μg/mL (Rodrigues et al., 2016).

The efficacy of 2,3-dihydrobenzofuran, a neolignan used to treat liver diseases and vascular diseases of the brain and found in propolis and other plants, against *L. amazonensis* was studied. This compound showed IC_50_ values of 1.04 and 1.4 μM for promastigotes and intracellular amastigotes, respectively. The intracellular amastigote activity may be mediated by the activation of macrophages, as *L. amazonensis*-infected BALB/c macrophages treated with 2,3-dihydrobenzofuran exhibited an increase in nitric oxide production, lysosomal volume, and macrophage phagocytic ability (De Castro Oliveira et al., 2017).

Saracoside and lyoniside, two lignans isolated from *Saraca indica*, were able to interact with *L. donovani* DNA, inducing apoptosis-like cell death. The IC_50_ values of lyoniside and saracoside against the intracellular amastigote were 0.79 and 0.82 μM, respectively. BALB/c mice infected with *L. donovani* were treated intraperitoneally with both lignans (lyoniside and saracoside) at doses of 2.5 and 5 mg/kg/day. Both doses of lyoniside and saracoside were capable of significantly decreasing the parasite loads in the spleen and liver (Saha et al., 2013).

Dyphylin, an arylnaphthalene lignin isolated from *Haplophyllum bucharicum*, is known to have activity against viruses and cancers. In promastigotes of *L. infantum*, dyphylin exhibited an IC_50_ value of 14.4 μM. Furthermore, dyphylin was not determined to be cytotoxic for macrophages exhibiting a CC_50_ value of 32.2 μM. In the intracellular amastigote of *L. infantum*, dyphylin exhibited an IC_50_ value of 0.2 μM, reaching a selectivity index of 178. As a possible mechanism of action, dyphylin may interferes with the cell cycle and protein synthesis and increases intracellular lipid accumulation. However, dyphylin did not increase nitric oxide (NO) production (Di Giorgio et al., 2005).

Niranthin, a lignan from *Phyllanthus amarus*, was evaluated against *L. donovani*. The compound was able to inhibit *L. donovani* promastigote proliferation and exhibited good activity against intracellular amastigote with an IC_50_ value of 1.26 μM; this lignan was not observed to be toxic to macrophages. The effects of niranthin were also tested against an antimony-resistant strain of *L. donovani*. Niranthin exhibited an IC_50_ value of 1.68 μM, indicating that the compound is able to inhibit resistant amastigotes. It is essential for the drug discovery process to determine how natural compounds interfere in the host-parasite relationship, since the ideal compound should not be as toxic to the host as it is to the parasite. Niranthin was determined to have the ability to induce apoptosis (Chowdhury et al., 2012).

To verify the effect of niranthin *in vivo*, BALB/c mice were infected intracardially with *L. donovani* and treated with niranthin for 3 weeks at intraperitoneal or intramuscular doses of 5 and 10 mg/kg/day. Splenic and hepatic parasitic loads were almost completely eliminated at the dose of 10 mg/kg/day. Immunological analyses were performed, indicating the ability of niranthin to increase NO levels and switch from a Th2 response to a Th1 response (Chowdhury et al., 2012).

To optimize the choice of lignans and neolignans with potential effects against *Leishmnia* species and to prevent possible failures from being detected only in preclinical tests, several computational tools can contribute strongly to database creation by predicting protein functions, modeling protein structures, simulating metabolic pathway kinetics, predicting biological activities, predicting toxicity, and predicting the affinities and flexibilities between receptors and ligands, which can facilitate the development and identification of drugs with the potential to treat various diseases and promote the development of efficacious drugs with reduced toxicity (Maia et al., 2020).

The design and synthesis of analogs of natural compounds is a strategy extensively used to identify effective new treatments against leishmaniasis. Design analogs enable the enhancement of many biological and chemical characteristics of compounds to afford new hits, such as bioactivity, selectivity, water solubility, and lipophilicity (Meanwell, 2011).

Along with the approach of advanced technologies for the design of new biologically more potent drugs, it is essential to understand the biology of the parasite to direct these studies.

Two neolignans, threo, threo-manassantin A and threo, erythro-manassantin A, isolated from *Saururus cernuus* exhibited activity against promastigotes (IC_50_ of 35.4 and 17.6 μM, respectively) and intracellular amastigotes (IC_50_ of 20.4 and 16.0 μM, respectively) of *Leishmania amazonensis*. Regardless of the mode of action, these compounds seem to act directly on parasites, since host cells did not show signs of cell activation. Both molecules were determined to be able to interact with the parasite plasmatic membrane and to interfere with the parasite nucleus (Brito et al., 2019).

Virtual screening and experimental validation have been utilized to identify lignans with leishmanicidal potential, low toxicity, and selective activity against several *Leishmania* targets. A set of 160 lignans (i.e., 14 furans, 10 furofurans, 14 dibenzylbutyrolactols, 22 dibenzylbutanes, 21 dibenzocycloocyadienes, 17 aryltetralins, 3 arylnaphthalenes, 8 neolignan alkyl aryl ethers, 16 neolignan benzofurans, and 9 neolignan benzodiones) were selected using predictive models that were built using data for *L. major* and *L. braziliensis* (Maia et al., 2020).

In brief, the workflow consisted of predicting the ADMET properties of these lignans. Through this tool, 42 compounds have good lipophilicity, water solubility, pharmacokinetic action and low or no predicted risk for the development of mutagenicity, tumorigenesis, negative effects on the reproductive system, or irritability. Next, ligand-based virtual screening was performed to evaluate the potential antileishmanial activity of these compounds using the random forest (RF) algorithm with the parameters of specificity, sensitivity, accuracy, positive predicted value (PPV), and negative predicted value (NPV) for performance and robustness. This model was able to select 11 compounds with active potential, with probabilities ranging from 50 to 75%, for *L. major*, and 21 potentially active compounds against *L. braziliensis* were selected and exhibited the same probabilities.

To choose the potential targets in both *L. major* and *L. braziliensis*, sequence alignment was employed to verify the similarities and identities of the enzymes selected in this study across different species. In addition, differences and structural similarities could be identified that might contribute to rational drug planning. After sequence alignment and homology modeling were performed, three enzymes were chosen for both species: GPDH (glycerol-3-phosphate dehydrogenase), PTR1 (pteridine reductase 1), and TR (trypanothione reductase).

After all these virtual screenings, four lignans (secoisolariciresinol, pinoresinol-4-O-β-_D_-glucopyranoside, epipinoresinol-4-O-β-_D_-glucopyranoside, and pinoresinol-4-O-β-d-apiofuranosyl-(1 → 2)-β-d-glucopyranoside) were selected, and their potential to inhibit the growth of promastigote forms of *L. major* and *L. braziliensis* was tested. Epipinoresinol-4-O-β-d-glucopyranoside was the only compound that exhibited activity against both species tested, presenting IC_50_ values for *L. major* and *L. braziliensis* of 36.5 and 5.4 μM, respectively (Maia et al., 2020).

Lignans that have a defined IC_50_ are summarized in Table 2.

**Table 2:** Chemical structure and leishmanicidal activities of lignans and neoligans.

**Class**	**Subclass**	**Compound name and chemical structure**	***Leishmania* species**	**Assay**	**Values**	**References**
Neolignanes	Benzofuran	**2,3-Dihydrobenzofuran** 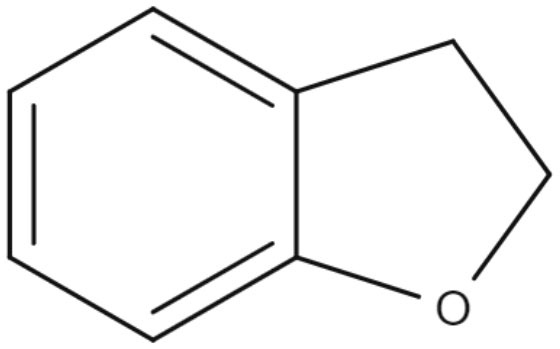	*L. amazonensis*	*In vitro*	**Promastigotes:** IC_50_: 1.04 μM **Intracellular amastigotes:** IC_50_: 1.4 μM	De Castro Oliveira et al., 2017
	Ortho-biphenyl	**Dehydrodieuginol (DHDE)** 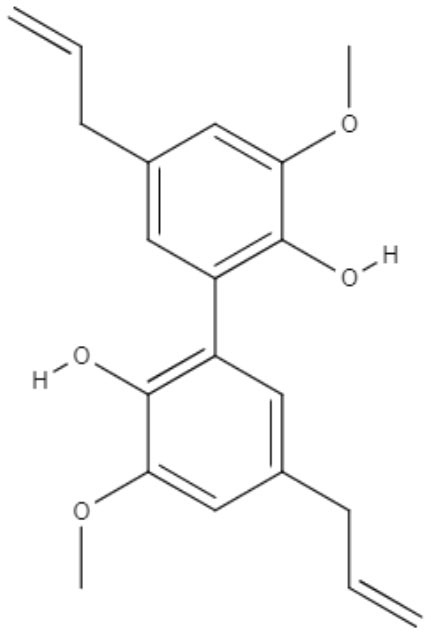	*L. amazonensis*	*In vitro*	**Promastigotes:** IC_50_: 42.4 μg/mL	Rodrigues et al., 2016
Neolignanes	Tetrahydrofuran dineolignans	**Threo,threo-manassatin A** 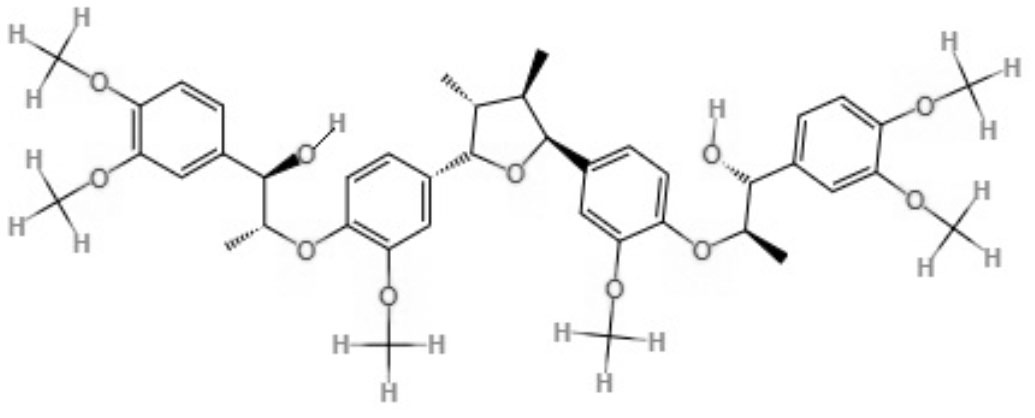	*L. amazonensis*	*In vitro*	**Promastigotes:** IC_50_: 35.4 μM **Intracelular amastigotes:** IC_50_: 20.4 μM	Brito et al., 2019
		**Erytro-manassatin A** 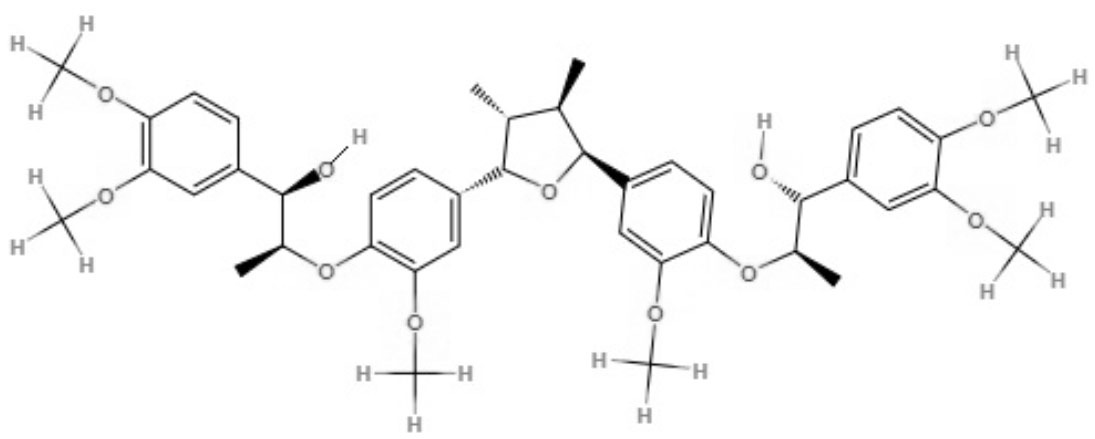			**Promastigotes:** IC_50_: 17.6 μM **Intracelular amastigotes:** IC_50_: 16.0 μM	
Lignans	Dibenzylbutanes	**Niranthin** 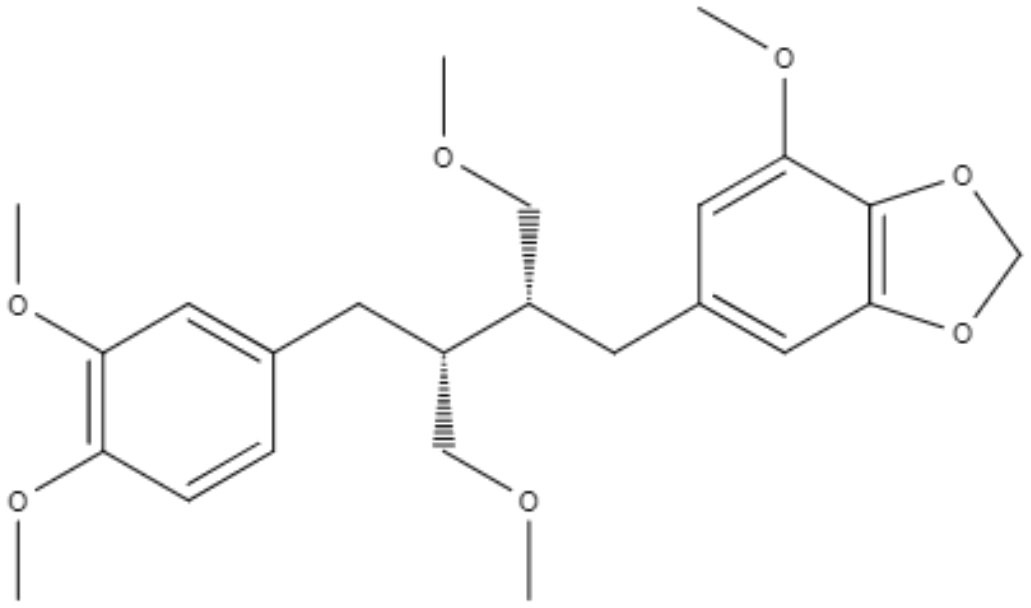	*L. donovani*	*In vitro*	**Intracellular amastigotes:** IC_50_: 1.26 μM **Antimony-resistant intracellular amastigotes:** IC_50_: 1.68 μM	Chowdhury et al., 2012
				*In vivo*	ND	
	Furofuran	**Epipinoresinol-4-O-β** **-**_**D**_**-glucopyranoside** 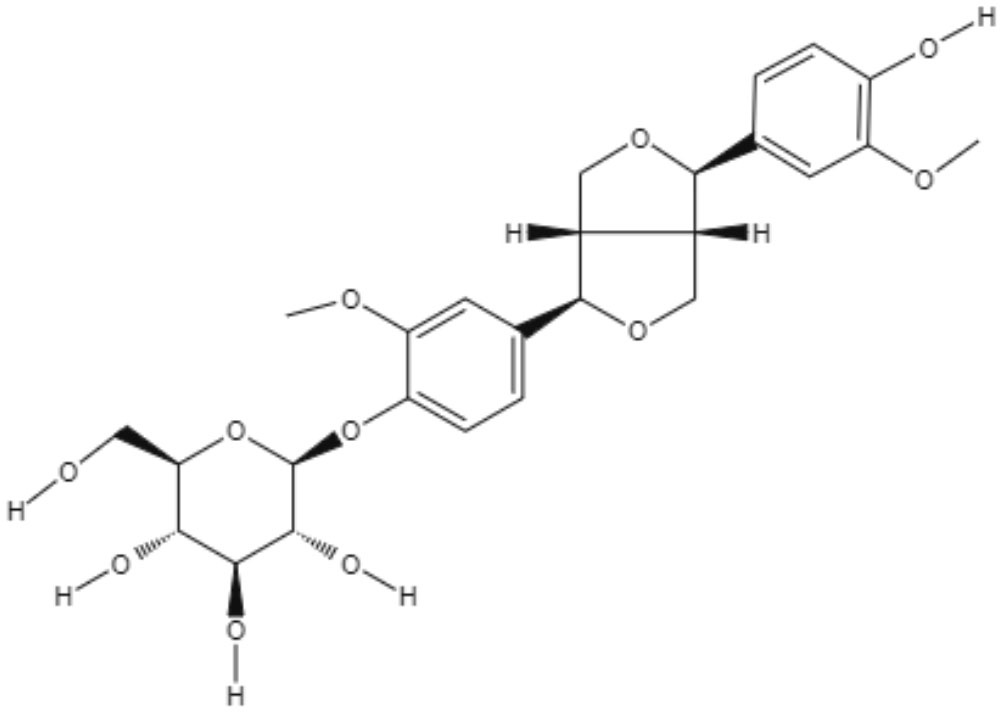	*L. major*	*In vitro*	**Promastigotes:** IC_50_: 36.5 μM	Maia et al., 2020
			*L. braziliensis*		**Promastigotes:** IC_50_: 5.4 μM	
Lignan	**Xanthine**	**Diphyllin** 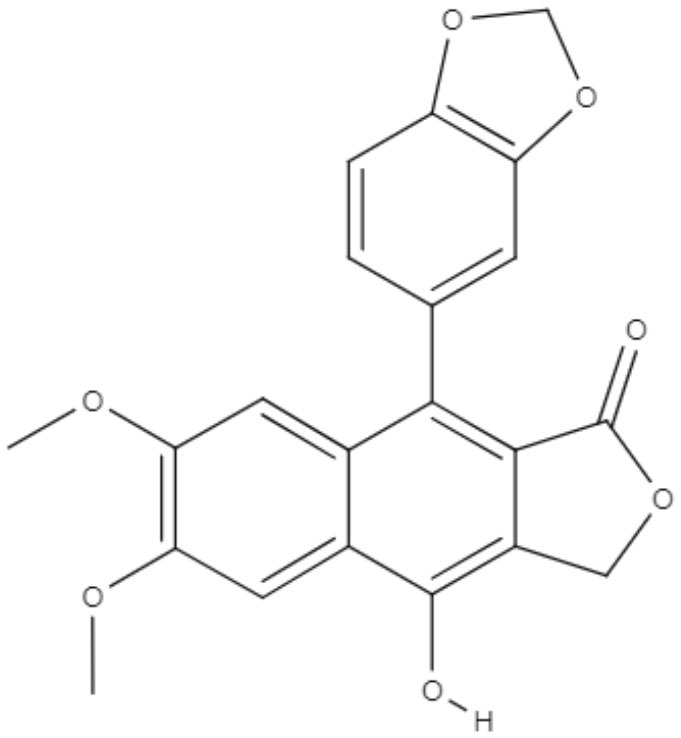	*L. infantum*	*In vitro*	**Promastigotes:** IC_50_: 14.4 μM **Intracelullar amastigotes:** IC_50_: 0.2 μM	Di Giorgio et al., 2005
	**Aryltetralin lignans**	**Lyoniside** 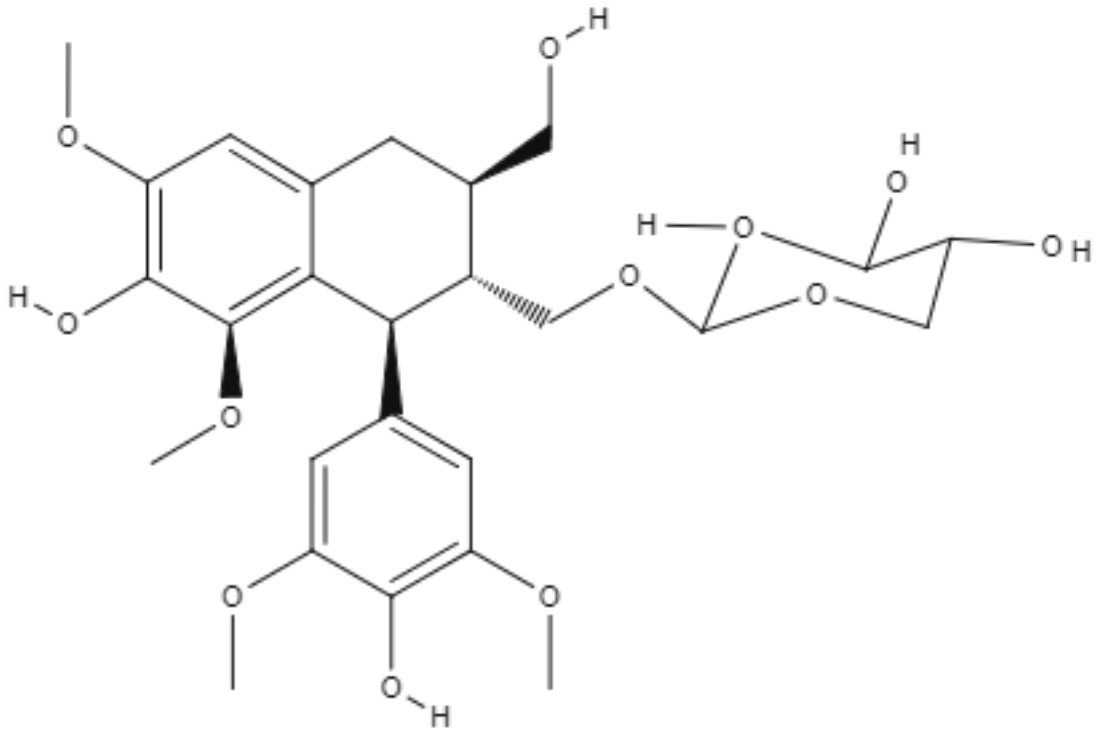	*L. donovani*	*In vitro*	**Intracelullar amastigotes:** IC_50_: 0.79 μM	Saha et al., 2013
				*In vivo*	ND	
**Lignan**	**Aryltetralin lignans**	**Sacaroside** 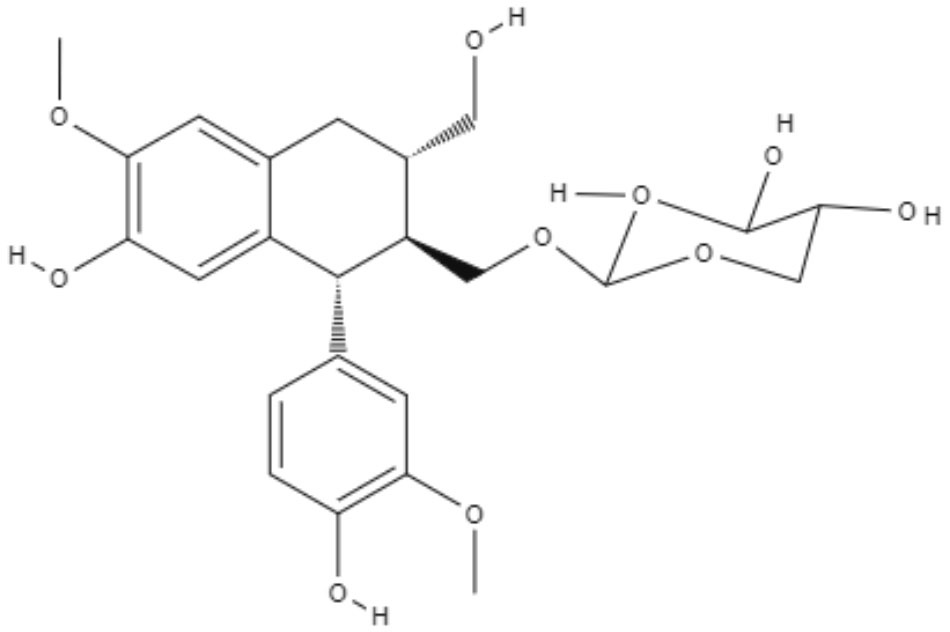	*L. donovani*	*In vitro*	**Intracelullar amastigotes:** IC_50_: 0.82 μM	Saha et al., 2013
				*In vivo*	ND	

*ND, Not determined*.

### Coumarins

Coumarins are derivatives that have a hydroxyl group, which differs in their biological properties. Many enzymes are related to coumarin biosynthesis; therefore, this group has various classes, such as simple coumarin, dimeric coumarin, furanocoumarin, and pyranocoumarin (Jain and Joshi, 2012). The diversity of structures within the coumarin group enables them to exhibit many biological activities, including anti-*Leishmania* activity. Various studies have described not only the *in vitro* activity of these compounds against *Leishmania* but also their mechanism of action and performance in preclinical studies. Mammaea A/BB, which were extracted from *Calophyllum brasiliense*, showed IC_50_ values of 7.4 μM against promastigotes and 14.3 μM against intracellular amastigotes of *Leishmania amazonensis* (Brezan et al., 2008). Regarding the mechanism of action, mammaea A/BB was able to induce mitochondrial membrane damage and cause changes in ultrastructure in *L. amazonensis* promastigotes (Brenzan et al., 2012). However, only when topically and intramuscularly administered did mammaea A/BB reduce the lesion sizes in mice infected with *L. amazonensis* compared to mice treated with meglumine antimoniate (Tiuman et al., 2012).

*Helietta apiculata* Benth is a native plant of Paraguay, Brazil, and Argentina and is popularly known as “canela-de-veado” in Brazil. (+)-3-(1′-dimethylallyl)-Decursinol and (-)-heliettin, two coumarins extracted from *Helietta apiculata* Benth, were tested against *L. amazonensis in vitro* and *in vivo*. In *L. amazonensis* promastigotes, the IC_50_ values were 35.8 μM and 18.5 μM for (+)-3-(1′-dimethylallyl)-decursinol and (-)-heliettin, respectively (Table 3). In the *in vivo* study, BALB/c mice infected with *L. amazonensis* were injected intraperitoneally with 10 mg/kg/day (+)-3-(1′-dimethylallyl)-decursinol or (-)-heliettin for 14 days. Both coumarins were capable of decreasing parasite loads similar to those observed when the reference drug, meglumine antimoniate, was used (Ferreira et al., 2010).

**Table 3:** Chemical structure and leishmanicidal activities of coumarins.

**Class**	**Subclass**	**Compound name and chemical structure**	***Leishmania* species**	**Assay**	**Values**	**References**
Coumarin	Dihydropyranocoumarins	**(+)-3-(1′-dimethylallyl)-decursinol** 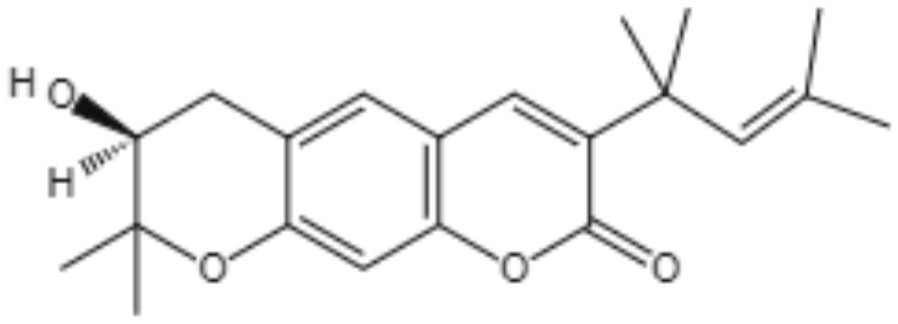	*L. amazonensis*	*In vitro*	**Promastigotes:** IC_50_: 35.8 μM	Ferreira et al., 2010
				*In vivo*	ND	
		**(-)-hellietin** 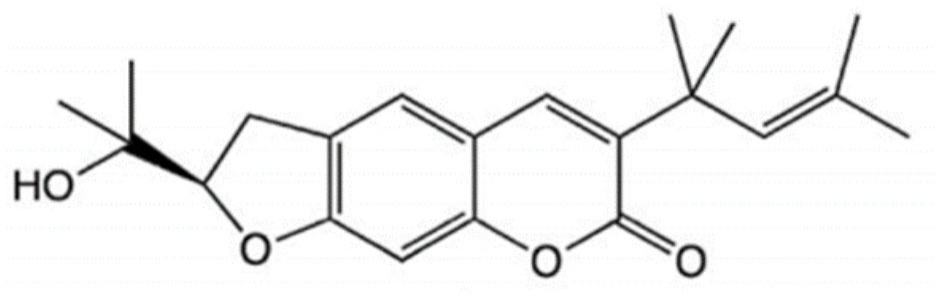		*In vitro*	**Promastigotes:** IC_50_: 18.5 μM	
				*In vivo*	ND	
Coumarin	Tricyclic pyranocoumarins	**Calanolide E1** 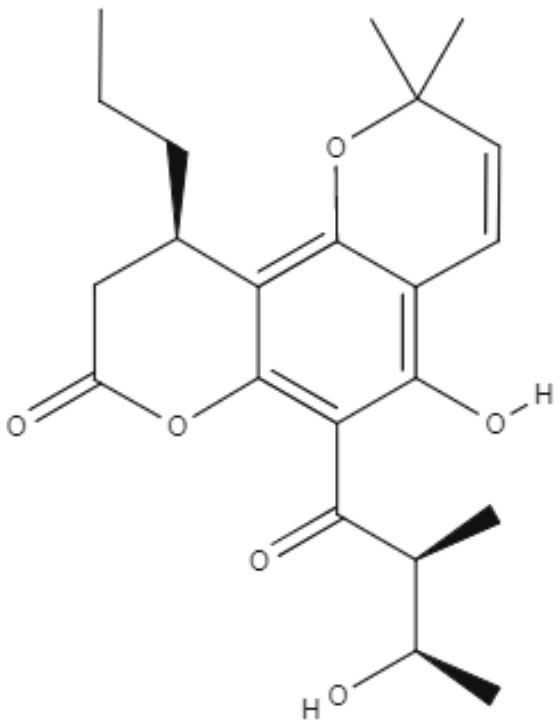	*L. major*	*In vitro*	**Promastigotes:** IC_50_: 36.5 μM	Silva et al., 2020
		**Calanolide E2** 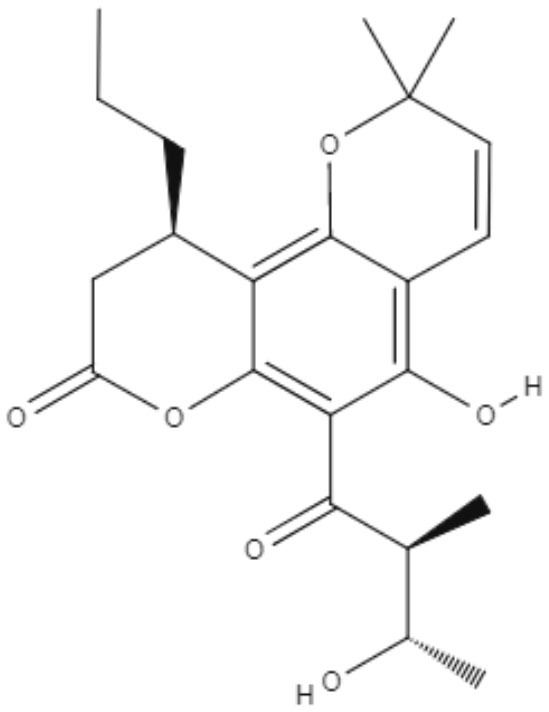			**Promastigotes:** IC_50_: 29.1 μM	
Coumarin	**Coumarin-type mammea**	**Mammae A/BB** 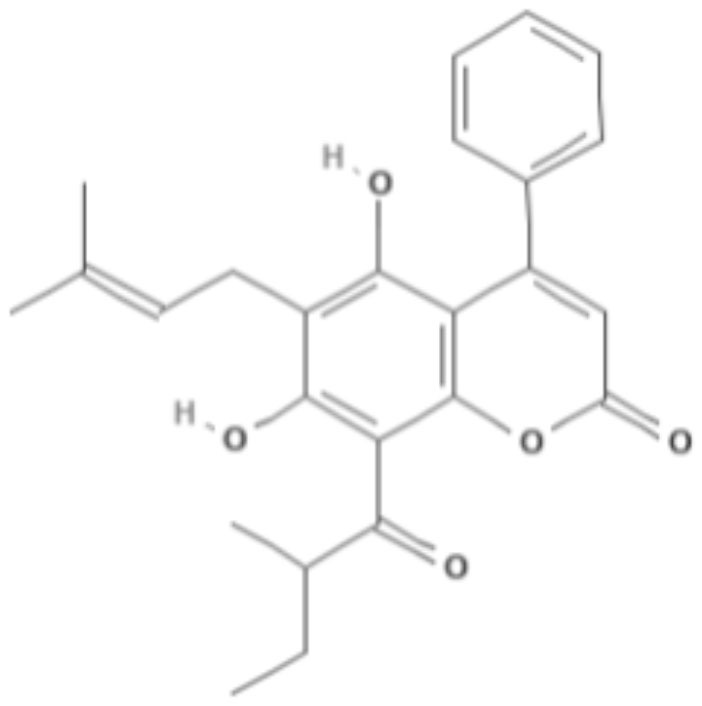	*L. amazonensis*	*In vitro*	**Promastigotes**: IC_50_: 3.0 μg/mL IC_90_:5 μg/mL **Axenic amastigotes**: IC_50_: 0.88 μg/mL IC_90_: 2.3 μg/mL	Brenzan et al., 2007
					**Promastigotes**: IC_50_: 7.4 ± 0.3 μM **Intracelullar amastigotes**: IC_50_: 14.3 ± 2.2 μM	Brezan et al., 2008
				*In vivo*	ND	Tiuman et al., 2012

*ND, Not determined*.

Part of their life cycle of *Leishmania* parasites occurs in the sand fly. Since these parasites develop entirely in the digestive system of the vector, interacting with digestive enzymes and other structures from the intestinal tract of the vector, little is known concerning the effect of plant-derived secondary metabolites during the interaction between parasites and vector or even on basic sand fly digestive physiology. Additionally, vector control is one of the key strategies for reducing the number of leishmaniasis cases, and it needs more research and development (Ferreira et al., 2018).

Based on this concept, Ferreira et al. (2010) tested the effect of two coumarins, esculin and esculetin, on sand flies infected with *L. infantum* and *L. mexicana*. These molecules were added to the sugar meal of *Lutzomia longipalpis*. Interestingly, esculetin significantly reduced the viability of *L. infantum* and *L. mexicana* in a concentration-dependent manner. Esculin also might block the transmission of leishmaniasis with no repellent effects or reduction in the amount of sugar ingested. In this way, these compounds may represent promising tools for starting the development of antiparasitic sugar baits with less selective pressure for resistance in vector populations (Ferreira et al., 2010). This work demonstrates that coumarin is a promising natural compound that can act on two fronts: as a treatment for leishmaniasis and as a tool to control leishmaniasis vectors.

Two coumarins obtained from stem bark of *Calophyllum brasiliense* demonstrated activity against amastigotes of *Leishmania infantum*. Calanolides E1 (1) and E2 (2) presented IC_50_ values of 37.1 and 29.1 μM, respectively (Table 3). The structure-activity relationship between compounds 1 and 2 was determined. Compound 2, corresponding to anti stereochemistry between carbons C-20 and C-30, showed higher activity against amastigote forms of *L. infantum*, suggesting that the configuration of C-30 plays an important role in the interaction of this derivative and the tested parasites (Silva et al., 2020).

Moreover, compounds 1 and 2 were subjected to substructure filtering to evaluate their PAINS characteristics. This analysis is crucial to the development of new lead compounds, since some physical/chemical proprieties of the studied compounds could be associated with their reactivity (non-covalent binding) or non-specific interactions with therapeutic targets of parasites (Silva et al., 2020). Both compounds did not contain any PAINS substructures; in other words, there is a reduced probability that their biological activities are artifacts caused by reactivity or colloidal aggregation. These data suggest that coumarins 1 and 2 may serve as scaffolds in the design of novel drug candidates for leishmaniasis (Silva et al., 2020).

Coumarins that have a defined IC_50_ are summarized in Table 3.

### Caffeic Acid

Generally observed in carbohydrate derivatives, such as glycosides, starches, esters and sugar esters, caffeic acids are the most representative hydroxycinnamic acids. Structural modifications, such as amides or esters, may increase the diversity of biological properties of new analogs. Radicals exhibiting 3,4-dihydroxy-substitution patterns have shown inhibitory properties and have attracted interest with respect to being used as drugs (Touaibia et al., 2012).

Computer tools have been used as a preview screening of compounds to evaluate whether the compounds have the chemical characteristics of an oral drug. Before bioguided assays of caffeic acid against *Leishmania* sp. were conducted, an *in silico* test of caffeic acid was performed to assess its potential as an oral drug. Molinspiration property calculation software (www.molinspiration.com) was used to calculate the parameters related to oral bioavailability according to Lipinski's rule of five. Lipinski's rule of five describes important molecular properties for a drug's pharmacokinetics in the human body with a high probability of human intestinal absorption and oral bioavailability. Caffeic acid satisfied Lipinski's rule of five with no violation, demonstrating that it is a good drug candidate for oral administration. After the *in silico* evaluation, bioguide assays were developed. Caffeic acid showed IC_50_ values of 12.5 μg/mL against promastigotes (Bortoleti et al., 2019), 16.0 μM against intracellular amastigotes of *Leishmania amazonensis* (Montrieux et al., 2014) and 21.9 μM for intracellular amastigotes of *L. infantum* (Garcia et al., 2019) (Table 4). Regarding these promising *in vitro* results, the effects of caffeic acid in an *in vivo* model of infection were examined. A preclinical trial of caffeic acid in BALB/c mice infected with *L. amazonensis* promastigotes was conducted. The caffeic acid treatment was administered by the intralesional route every 4 days for 30 days of the experiment. The treatment was able to reduce lesion sizes and parasitic loads in treated animals compared to untreated animals and animals treated with vehicle (Montrieux et al., 2014).

**Table 4:** Chemical structure and leishmanicidal activities of caffeic acid.

**Class**	**Compound name and chemical structure**	***Leishmania* species**	**Assay**	**Values**	**References**
Caffeic acid	Caffeic acid 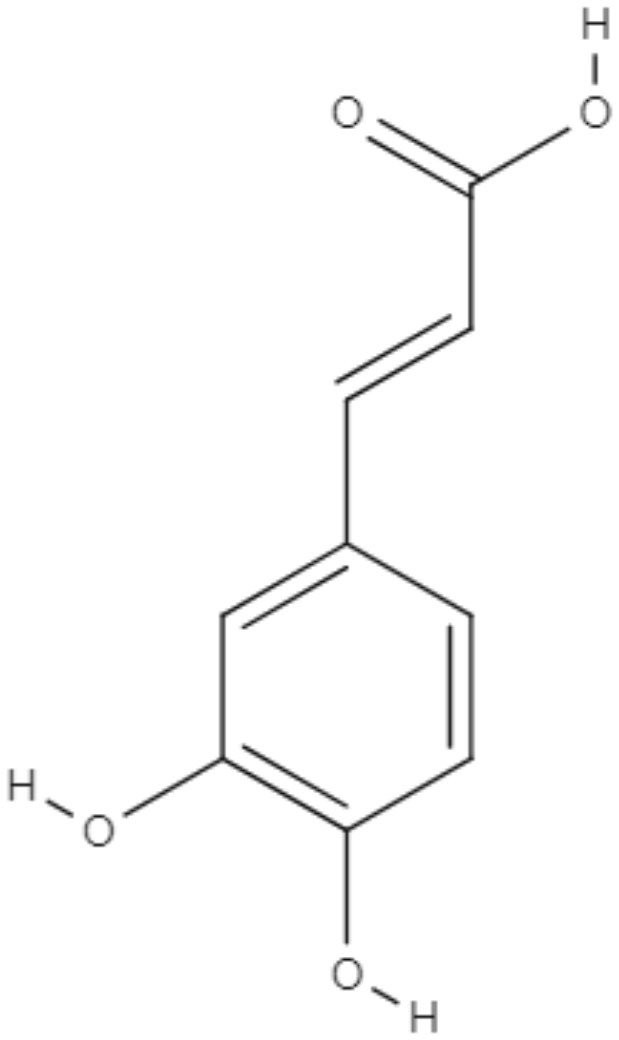	*L. amazonensis*	*In vitro*	**Promastigotes:** IC_50_: 5.2 μM **Intracellular amastigotes:** IC_50_: 16.0 μM	Montrieux et al., 2014
			*In vivo*	ND	
			*In vitro*	**Promastigotes:** IC_50_: 12.5 μg/mL	Bortoleti et al., 2019
			*In vivo*	ND	
		*L. infantum*	*In vitro*	**Intracellular amastigotes:** IC_50_: 21.9 μM	Garcia et al., 2019

*ND, Not determined*.

As a mechanism of action, caffeic acid was able to alter promastigote cell morphology and cell volume accompanied by loss of mitochondrial integrity, increase in reactive oxygen species (ROS) production, phosphatidylserine exposure, and loss of plasma membrane integrity, suggesting an apoptosis-like process. Caffeic acid also increased TNF-α, ROS, and NO and reduced IL-10 levels, as well as iron availability (Bortoleti et al., 2019; Garcia et al., 2019). Through these results, it is possible to conclude that caffeic acid has leishmanicidal effects with a mechanism of action that triggers multiple targets that affect the viability of the parasite.

Although caffeic acid has properties that make it a good candidate for oral drugs, its distribution in biological systems is limited due to its hydrophobic nature (Durak et al., 2020).

However, it is important to note that no studies were identified that employed advanced technologies in the investigation of caffeic acid or its derivatives in *Leishmania* spp. or evaluated the pharmacokinetics of this substance. One hypothesis for this scarcity of published research is that although caffeic acid has had its biological effects characterized, its limitations in relation to bioavailability, such as its hydrophobic nature, make the synthesis of this compound difficult (Durak et al., 2020).

### Quinones

Based on their aromatic carbon skeletons, quinones can be classified as benzoquinones, anthraquinones and naphthoquinones. The benzoquinones comprise ubiquinone and plastoquinone, which differ in their substitution patterns and exhibit different levels of unsaturation on their side chain. Ubiquinones are involved in respiratory chain reactions. Anthraquinones are the oldest known compounds that are used as colorants. Naphthalene is the most natural naphthoquinone and is important in medicinal chemistry because it exerts biological effects on various pathogens, such as *Leishmania* (Schmidt et al., 2012b).

Plumbagin, a naphthoquinone extracted from *Pera benensis*, was tested against *L. donovani* and exhibited an excellent IC_50_ value of 0.34 and 0.21 μM for promastigotes and axenic amastigotes, respectively. It has been shown that the possible mechanism of action of this compound involves the non-competitive inhibition of trypanothione reductase, a key enzyme in *Leishmania* redox homeostasis, leading to an increase in reactive oxygen species and changing the redox balance (Sharma et al., 2012).

Buparvaquone (BPQ) and its phosphate prodrugs (BPQ-3-PHOS and 3-POM-BPQ) are hydroxynaphthoquinone, which were tested against several species of *Leishmania (L. major, L. amazonensis, L. aethiopica, L. mexicana*, and *L. panamensis*) *in vitro*. BPQ, BPQ-3-PHOS, and 3-POM-BPQ demonstrated low IC_50_ values against promastigotes and amastigotes of all *Leishmania* species tested, exhibiting better activity than amphotericin B and pentostan (Mäntylä et al., 2004). The IC_50_ values of BPQ, BPQ-3-PHOS, and 3-POM-BPQ for each *Leishmania* species are described in Table 5.

**Table 5:** Chemical structure and leishmanicidal activities of quinones.

**Class**	**Subclass**	**Compound name and chemical structure**	***Leishmania* species**	**Assay**	**Values**	**References**
Quinones	**Hydroxynaphthoquinones**	**Buparvaquone (BPQ)** 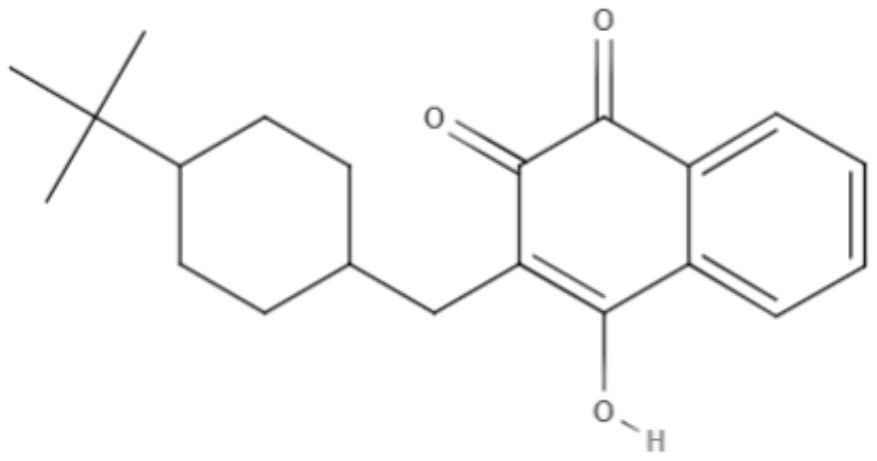	*L. donovani*	*In vitro*	**Promastigotes:** IC_50_: 0.006 μM **Intracellular amastigotes:** IC_50_: 0.04 μM	Mäntylä et al., 2004
			*L. aethiopica*		**Promastigotes:** IC_50_: 0.013 μM **Intracellular amastigotes:** IC_50_: 3.6 μM	
			*L. major*		**Promastigotes:** IC_50_: 0.001 μM **Intracellular amastigotes:** IC_50_: 1.8 μM	
			*L. amazonensis*		**Promastigotes:** IC_50_: 0.004 μM **Intracellular amastigotes:** IC_50_: 5.5 μM	
			*L. mexicana*		**Promastigotes:** IC_50_: 0.004 μM **Intracellular amastigotes:** IC_50_: 1.3 μM	
			*L. panamensis*		**Promastigotes:** IC_50_: 0.04 μM **Intracellular amastigotes:** IC_50_: 0.9 μM	
Quinones	**Hydroxynaphthoquinones**	**Buparvaquone-3-phosphate (BPQ-3-PHOS)** 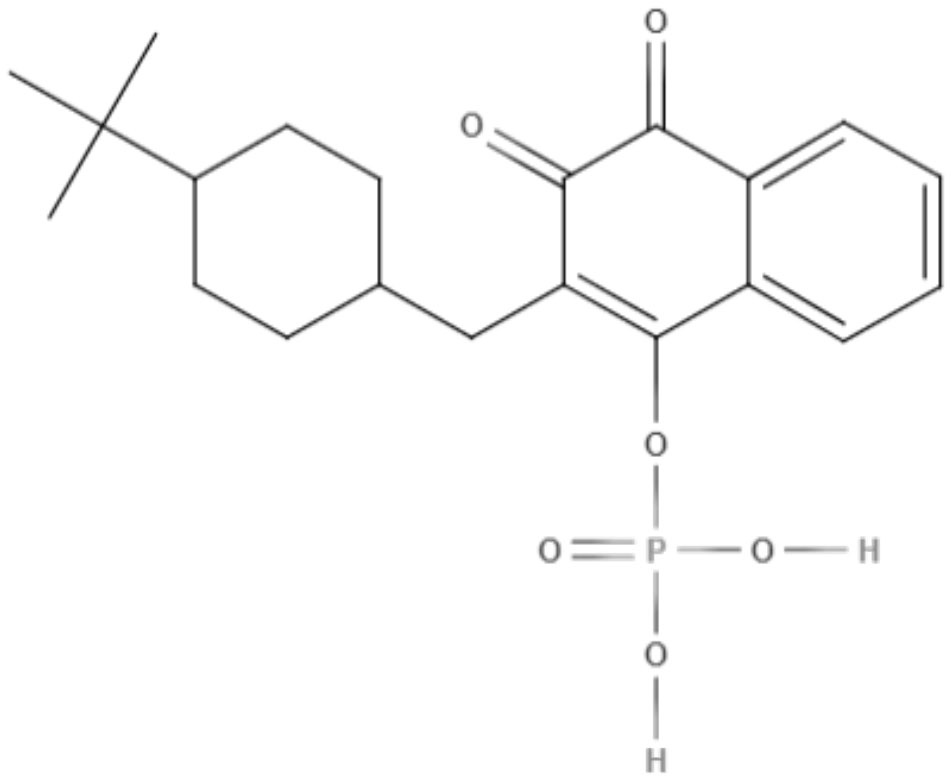	*L. donovani*	*In vitro*	**Promastigotes:** IC_50_: 0.009 μM **Intracellular amastigotes:** IC_50_: 4.3 μM	Mäntylä et al., 2004
			*L. aethiopica*		**Promastigotes:** IC_50_: 0.1 μM **Intracellular amastigotes:** IC_50_: 7.4 μM	
			*L. major*		**Promastigotes:** IC_50_: 0.06 μM **Intracellular amastigotes:** IC_50_: 6.3 μM	
			*L. amazonensis*		**Promastigotes:** IC_50_: 0.02 μM **Intracellular amastigotes:** IC_50_: 15.7 μM	
			*L. mexicana*		**Promastigotes:** IC_50_: 0.04 μM **Intracellular amastigotes:** IC_50_: 4.0 μM	
			*L. panamensis*		**Promastigotes:** IC_50_: 0.1 μM **Intracellular amastigotes:** IC_50_: 2.1 μM	
Quinones	Hydroxynaphthoquinones	**3-phosphonooxymethyl-buparvaquone (3-POM-BPQ)** 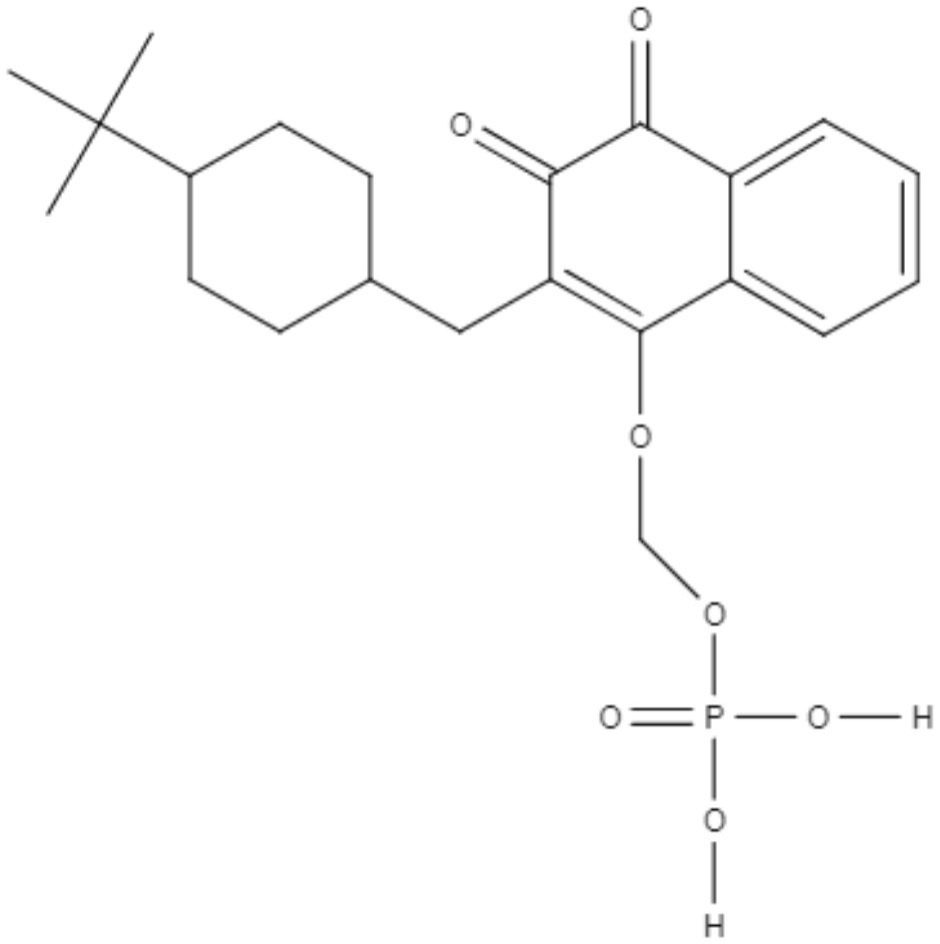	*L. donovani*	*In vitro*	**Promastigotes:** IC_50_: 0.003 μM **Intracellular amastigotes:** IC_50_: 0.1 μM	Mäntylä et al., 2004
			*L. aethiopica*		**Promastigotes:** IC_50_: 0.06 μM **Intracellular amastigotes:** IC_50_: 3.99 μM	
			*L. major*		**Promastigotes:** IC_50_: 0.012 μM **Intracellular amastigotes:** IC_50_: 1.9 1 μM	
			*L. amazonensis*		**Promastigotes:** IC_50_: 0.007 μM **Intracellular amastigotes:** IC_50_: 8.8 μM	
			*L. mexicana*		**Promastigotes:** IC_50_: 0.01 μM **Intracellular amastigotes:** IC_50_: 2.5 μM	
			*L. panamensis*		**Promastigotes:** IC_50_: 0.12 μM **Intracellular amastigotes:** IC_50_: 1.2 μM	
Quinones	Hydroxynaphthoquinones	**BPQ** **BPQ-3-PHOS** **3-POM-BPQ**	*L. major*	*In vivo*	ND	Garnier et al., 2007
			*L. donovani*			
		**BPQ-SNEDDS^a^**	*L. infantum*	*In vitro*	**Promastigotes:** IC_50_: 3.3 μg/mL **Intracellular amastigotes:** IC_50_: 0.09 μg/mL	Smith et al., 2018
				*In vivo*		
		**BPQ solid SNEDDS^b^**	*L. infantum*	*In vitro*	**Promastigotes:** IC_50_: < 0.012 μg/mL **Intracellular amastigotes:** IC_50_: < 0.005 μg/mL	Smith et al., 2018
				*In vivo*		
		**BPQ-NLC^c^**	*L. infantum*	*In vitro*	**Intracellular amastigotes:** IC_50_: 229.0 nM	Monteiro et al., 2019
		**BPQ-NLC-PB-[A**^**−**^**]^d^**			**Intracellular amastigotes:** IC_50_: 145.7 nM	
		**BPQ-NLC-PB-[C**^**+**^**]^e^**			**Intracellular amastigotes:** IC_50_: 150.5 nM	
Quinones	Naphthoquinones	**Plumbagin** 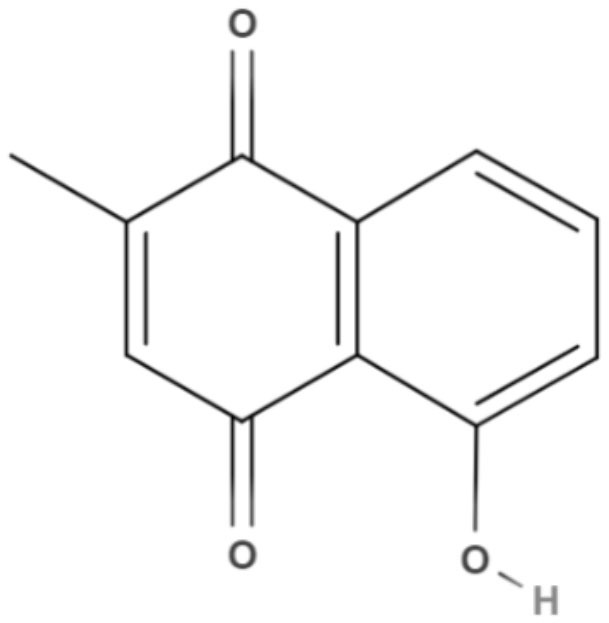	*L. donovani*	*In vitro*	**Promastigotes:** IC_50_: 0.34 μM **Axenic amastigotes:** IC_50_: 0.21 μM	Sharma et al., 2012
		**2-phenoxy-naphthoquinone** 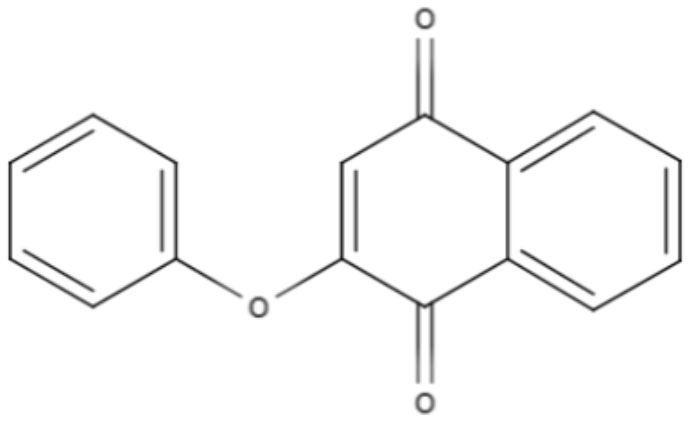	*L. donovani*	*In vitro*	**Promastigotes:** IC_50_: 0.74 μM **Axenic amastigotes:** IC_50_: 1.26 μM	Lizzi et al., 2012
	Anthraquinones	**2-phenoxy-anthraquinone** 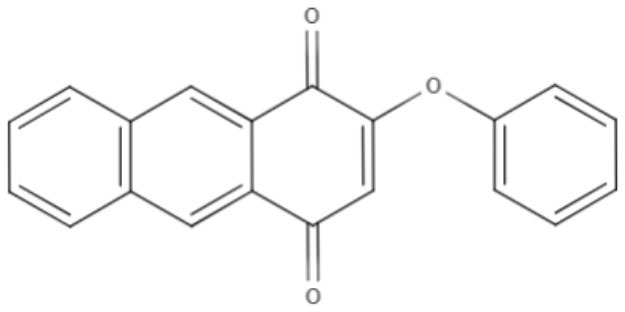	*L. donovani*	*In vitro*	**Promastigotes:** IC_50_: 2.8 μM **Axenic amastigotes:** IC_50_: 0.34 μM	Lizzi et al., 2012
Quinones	Anthraquinones	**Soranjidiol (Sor)** 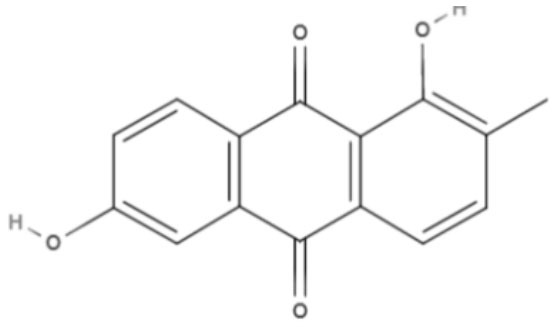	*L. amazonensis*	*In vitro*	**Promastigotes:** LD_50_: 16.3 J/cm^2^ LD_90_: 22.1 J/cm^2^	Dimmer et al., 2019
		**5-Chlorosoranjidiol (5-ClSor)** 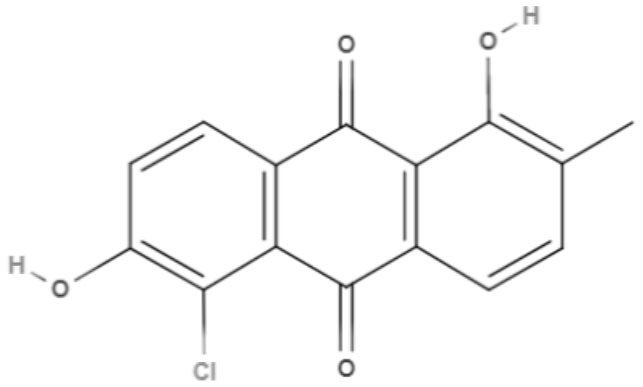	*L. amazonensis*	*In vitro*	**Promastigotes:** LD_50_: 13.8 J/cm^2^ LD_90_: 22.2 J/cm^2^	Dimmer et al., 2019
		**Bisoranjidiol (Bisor)** 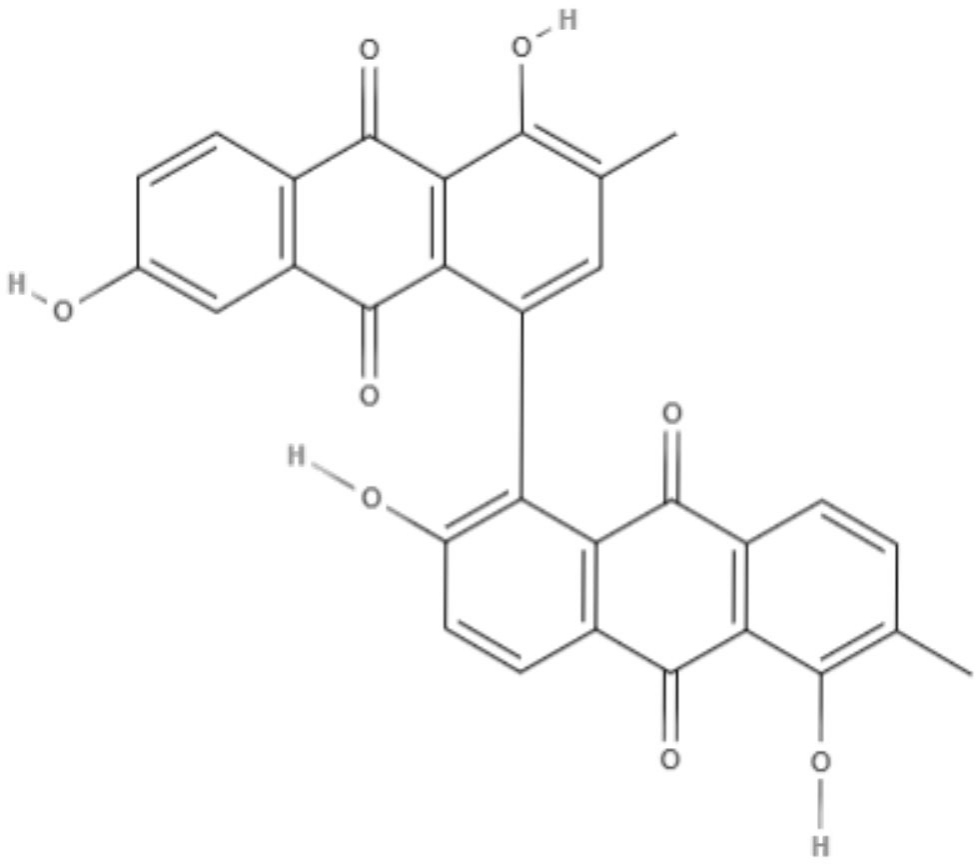	*L. amazonensis*	*In vitro*	**Promastigotes:** LD_50_: 15.2 J/cm^2^ LD_90_: 19.3 J/cm^2^	Dimmer et al., 2019
Quinones	Anthraquinones	**7-chlorobisoranjidiol (7-ClBisor)** 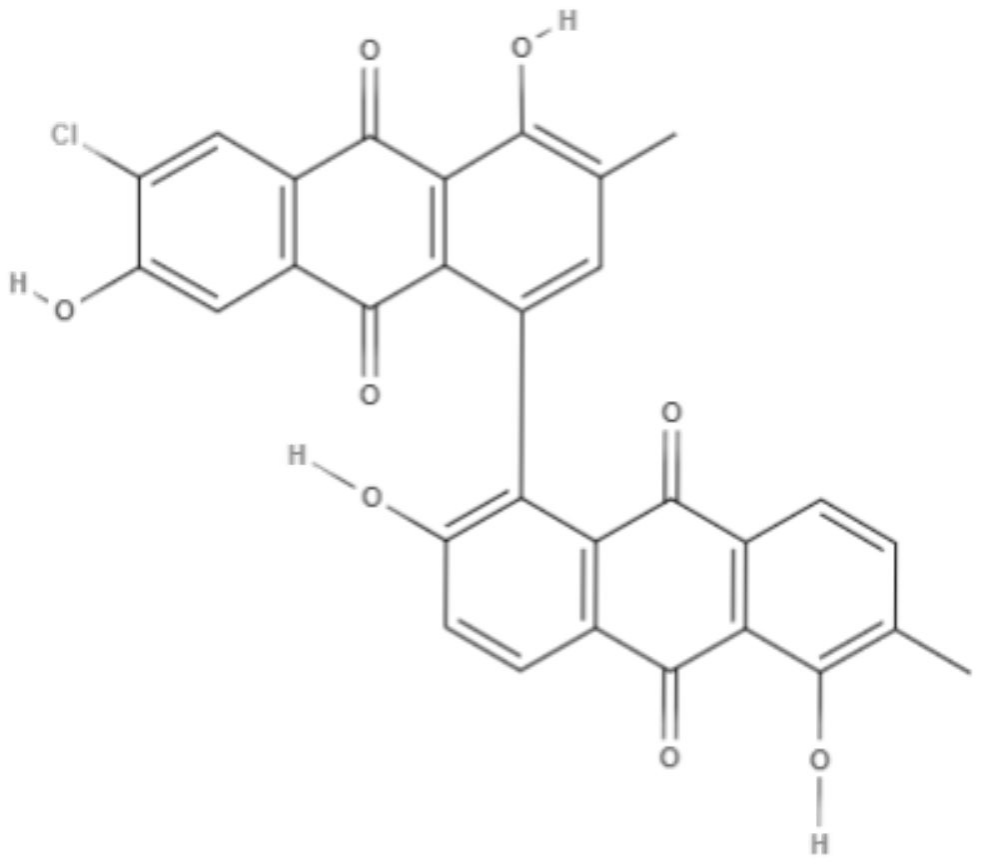	*L. amazonensis*	*In vitro*	ND	Dimmer et al., 2019
		**Lycionine (Lyc)** 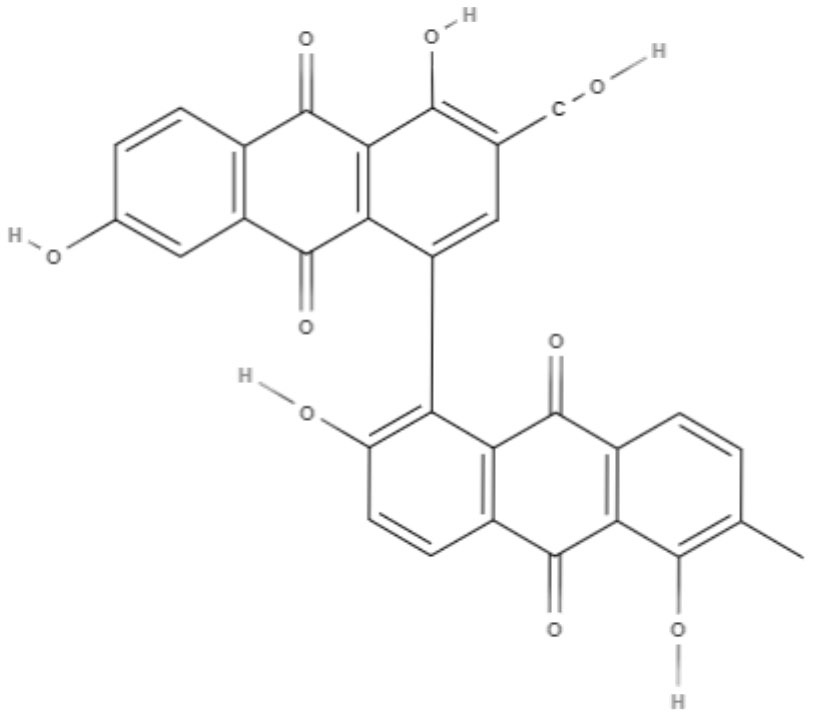	*L. amazonensis*	*In vitro*	ND	Dimmer et al., 2019

a *Buparvaquone (BPQ) loaded self-nanoemulsifying drug delivery system (SNEDDS)*.

b *BPQ loaded self-nanoemulsifying drug delivery system compressed into tablet*.

c *BPQ delivered by nanostructured lipid carrier (NLC)*.

d *BPQ co-delivered by nanostructured lipid carrier (NLC) and polymyxin B (PB)–anionic formulation*.

e *BPQ co-delivered by nanostructured lipid carrier and polymyxin B–cationic formulation*.

*ND, Not demonstrated; LD_50_, Light Dose that cause 50% of promastigote lethality; LD_90_, Light Dose that cause 90% of promastigote lethality*.

BPQ, BPQ-3-PHOS, and 3-POM-BPQ were also tested *in vivo* with different topical formulations as a hydrous gel, an anhydrous gel and an emulsion, targeting *Leishmania major*-infected BALB/c mice (Garnier et al., 2007). The hydrous gel formulation produced the best results. This formulation inhibited the infiltration of infected cells and decreased parasitic load by approximately 50%. These formulations were additionally tested against *L. donovani*-infected BALB/c mice. BPQ-3-PHOS was demonstrated to be the most active compound, with a decrease of approximately two-thirds in the liver parasite burden compared to the untreated control (Garnier et al., 2007).

It has previously been described that BPQ is poorly soluble in water with a lower *in vivo* activity (Croft et al., 1992). To overcome this limitation, a novel BPQ-loaded self-nanoemulsifying drug delivery system (BPQ-SNEDDS and BPQ solid SNEDDS) was developed. These formulations demonstrated activity against *L. infantum* promastigote and intracellular amastigote forms that was superior to miltefosine (Smith et al., 2018). An oral pharmacokinetic assay in mice was performed, and the BPQ-SNEDDS showed good bioavailability, increasing the AUC_0−24_ by 55%. During *in vivo* infection, BPQ-SNEDDS and BPQ solid SNEDDS were able to inhibit parasite replication in the spleen and liver of infected mice. These formulations are promising and may be able to overcome the limitations found in the use of BPQ, and further studies are warranted to provide more information regarding their effects (Smith et al., 2018).

In a similar approach, three different nanostructured formulations (BPQ-NLC, BPQ-NLC-PB-[A^−^] and BPQ-NLC-PB-[C^+^]) were developed and employed against *L. infantum*. All formulations showed lower IC_50_ values (229.0 nM, 145.7 nM, 150.5 nM for BPQ-NLC, BPQ-NLC-PB-[A^−^] and BPQ-NLC-PB-[C^+^], respectively) than did free BPQ, thereby improving the antiamastigote activity of this compound (Monteiro et al., 2019).

Over the years, natural product libraries and collections have been successfully established, enabling investigators to link chemical classes to biological activities. In recent years, quinone activity has been exploited, and a library of quinone-polyamine conjugates has been constructed. These conjugates were tested against three species known to cause human parasitic diseases, including *L. donovani*. Some derivatives were determined to inhibit the activity of trypanothione reductase. All compounds presented good IC_50_ against *L. donovani*, with emphasis being placed on compounds 2-phenoxy-anthraquinone and 2-phenoxy-naphthoquinone, which demonstrated the best IC_50_ values against the axenic amastigotes (0.34 and 1.26 μM, respectively) and promastigotes (2.8 and 0.74 μM, respectively) of *L. donovani* (Lizzi et al., 2012).

Using a different approach to topical treatment of cutaneous leishmaniasis, Dimmer et al. (2019) tested the antiparasitic photodynamic inactivation of soranjidiol (Sor) and its derivatives 5-chlorosoranjidiol (5-ClSor), bisoranjidiol (Bisor), 7-chlorobisoranjidiol (7-ClBisor), and lycionine (Lyc). Sor and its derivatives are anthraquinones isolated from *Heterophyllaea pustulata* Hook f. Photodynamic inactivation (PDI) is a methodology that combines photosensitive drugs with light to kill parasites. Light excites photosensitive molecules that generate ROS in the presence of oxygen. Soranjidiol, 5-chlorosoranjidiol and bisoranjidiol combined with violet-blue light caused a decrease in parasite viability of *L. amazonesis* promastigotes. Bisoranjidiol-mediated PDI induced significant alterations in the size and shape of promastigotes. Furthermore, soranjidiol is the most efficient anthraquinone to combat leishmaniasis, causing fewer toxic effects in fibroblast cells (Dimmer et al., 2019).

The quinones that were presented along with their leishmanicidal activities (IC_50_) are summarized in Table 5.

## Compounds From Amino Acid Pathways

### Alkaloids

Alkaloids are nitrogenous compounds with alkaline character. However, there are some exceptions, with certain compounds containing amino or amido atoms. Alkaloids are classified based on the presence and activity of specific amino acids, which form a fundamental component of the alkaloid skeleton. For example, the amino acid lysine produces piperidine, quinolizidine and indolizidine alkaloids, and the amino acid ornithine produces pyrrolidine and tropane alkaloids. The amino acid tyrosine gives rise to phenylethylamines and tetrahydroidoquinoline alkaloids. Tyrosine also produces other alkaloids in which phenolic oxidative links play a fundamental role (Kurek, 2019).

All alkaloids possessing amino acid precursors are true alkaloids or protoalkaloids. True alkaloids share a common heterocyclic ring with one nitrogen atom, while the main characteristic of protoalkaloids is a nitrogen atom that does not belong to the heterocyclic ring, such as cocaine. However, many alkaloids do not originate from amino acids but from the amination of other substrates, such as steroids, terpenoids, acetates and phenylalanine. Some authors have classified alkaloids that do not originate from amino acids as pseudoalkaloids. Finally, alkaloids that are produced via pathways resembling those by which purine nucleic acids are produced are classified as purine alkaloids (Dewick, 2009).

Many types of alkaloids have been described as having biological activities against trypanosomatids, such as *Leishmania* spp. Two heterocyclic steroids were isolated from *Solanum lycocarpum*, and their *in vitro* and *in vivo* activities were tested. Against *L. mexicana*, intracellular amastigote forms, solamargine, and solasonine showed IC_50_ values of 6.03 and 5.9 μM, respectively. These IC_50_ values were superior to the IC_50_ observed with sodium stibogluconate (Lezama-Dávila et al., 2016).

Interestingly, solamargine and solasonine induced different immunochemical pathways in macrophages and dendritic cells. *L. mexicana* was eliminated more efficiently by dendritic cells when incubated with solamargine and solasonine at a concentration of 10 μM. Additionally, both compounds were capable of enhancing the expression levels of transcription factors, such as NFκB/AP-1, also at a concentration of 10 μM. Nitric oxide levels decreased in both macrophages and dendritic cells only after treatment with solamargine, indicating that its mechanism of action is dependent on nitric oxide (Lezama-Dávila et al., 2016).

The *in vivo* study was performed using C57BL/6 mice infected with *L. mexicana.* Treatment with topical formulations of 10 μM solamargine and solasonine significantly reduced parasite loads and lesion sizes in the ear (Lezama-Dávila et al., 2016).

Combination therapy has been employed as a strategy for improving the treatment of leishmaniasis. The combination of piperine and its analog capsaicin with meglumine antimoniate has been tested. Against *L. infantum*, both alkaloids alone showed better antipromastigote activity than meglumine antimoniate with IC_50_ values of 5.01 μg/mL for capsaicin and 3.03 μg/mL for piperine. The combinations of piperine or capsaicin with meglumine antimoniate (50% + 50%) were the most effective against promastigotes, exhibiting IC_50_ values of 2.1 and 2.9 μg/mL, respectively. The best antiamastigote activity occurred in the combination of piperine with meglumine antimoniate (25% + 75%), presenting a synergistic effect with an IC_50_ value of 7.3 μg/mL (Vieira-Araújo et al., 2018).

It has already been described that piperine has a bioavailability enhancing effect (Randhawa et al., 2011). To explore this property and improve the bioavailability of amphotericin B, nanoformulations of piperine (PIP), and amphotericin B (AmB) coated with nanoparticles (HDGG-AmB-Pip-NPs and Eu-HDGG-AmB-Pip-NPs) were developed, and their leishmanicidal effects were evaluated. Both formulations showed good IC_50_ against promastigotes (21.9 and 24 ng/mL, respectively) and intracellular amastigotes (4.9 and 18.3 ng/mL, respectively) of *L. donovani*. When formulations were administered in *L. donovani*-infected golden hamsters by the intraperitoneal route, HDGG-AmB-Pip-NPs and Eu-HDGG-AmB-Pip-NPs reduced parasite load by 95 and 96%, respectively, compared to the untreated control and were more effective than amphotericin B treatment (Ray et al., 2020).

Pharmacokinetics analysis showed that Eu-HDGG-AmB-Pip-NPs improved the plasma concentration-time profile of amphotericin B compared to amphotericin B treatment. The tissue distribution was evaluated, and Eu-HDGG-AmB-Pip-NPs showed the highest amphotericin B accumulation in the liver and spleen. This compound was also detected in the kidney, but at the lowest concentration, it was detected in the liver and spleen. Furthermore, Eu-HDGG-AmB-Pip-NPs cause changes in serum levels. The use of piperine in association with reference drugs for the treatment of leishmaniasis in nanoformulations shows promising results that should be further explored (Ray et al., 2020).

*Senna spectabilis* is a tree of the family Fabaceae, and piperidine alkaloids, such as (-)-cassine, (-)-spectaline, (-)-3-o-acetylcassine and (-)-3-O-acetylspectaline, can be extracted from it. These alkaloids were tested against *L. amazonensis* promastigotes, and all of them presented leishmanicidal effects, with compound (-)-spectaline being more effective (IC_50_ = 15.8 μg/mL). However, the IC_50_ value of this compound was higher than that of amphotericin B. In addition, all piperidine alkaloids showed less toxicity than amphotericin B. In a more modern approach, *in silico* analysis using molecular docking was performed to evaluate how these piperidine alkaloids bound to the enzyme arginase. The alkaloid (-)-spectaline showed a stronger interaction with arginase than other alkaloids, suggesting arginase as a possible target for the (-)-spectaline (Lacerda et al., 2018).

Berberine is an isoquinoline alkaloid and is extracted from *Berberis vulgaris*. Previous studies have shown that berberine has leishmanicidal activity and can induce a redox imbalance following the enhanced generation of ROS (Saha et al., 2009). Since *Leishmania* has only one mitochondrion, which is the major ROS productor, the effects of berberine on the mitochondria of non-pathogenic *Leishmania donovani* UR6 were tested. This alkaloid showed a reduction in the viability of promastigotes in a concentration-dependent manner with an IC_50_ value of 4.8 μM and stimulated the generation of ROS in these cells. Berberine was also able to increase the levels of mitochondrial superoxide of promastigotes and induced depolarization of mitochondrial transmembrane potential. Concentration-dependent inhibition of complex I-III and II-III activities was observed in promastigotes, as well as a decrease in ATP levels. Although berberine has been tested on non-pathogenic parasites, these data provide support for future studies to search for a possible mechanism of action for this promising alkaloid (De Sarkar et al., 2018).

In the search for a new approach to improve the treatment of cutaneous leishmaniasis, the leishmanicidal effects of chitosan (CS)-polyethylene oxide (PEO) nanofibers containing berberine were tested. CS-PEO-Berberine showed an IC_50_ of 0.2 and 0.9 μg/mL against promastigotes and in intracellular amastigotes of *L. major*, respectively, indicating that this formulation can be provided as a good alternative topical treatment for cutaneous leishmaniasis (Rahimi et al., 2020).

Colchicine, demecolcine, and thiocolchicoside are tropolone alkaloids extracted from *Colchicum kuurdicum* (Bornm.) Stef., a perennial monocotyledon plant. Eight tropolones (colchicoside, 2-demethyl colchicine, 3-demethyl colchicine, demecolcine, colchifoline, N-deacetyl-N-formyl colchicine and cornigerine) were isolated from *Colchicum kuurdicum* and tested against *L. major*. All tropolones showed good IC_50_ and leishmanicidal effects. Colchicoside and colchicine were the most effective, exhibiting IC_50_ values of 4.0 and 8.7 μg/mL against intracellular amastigote forms. All tropolones presented *in vitro* iron chelating activity between 19 and 25%, with colchicine showing the highest activity. Tropolones also demonstrated significant anti-inflammatory effects, with anti-denaturation effects of between 50 and 80%. Additionally, these compounds caused only 5% hemolysis, demonstrating safety for systemic usages. To analyze toxicity, a brine shrimp toxicity test and an acute toxicity test in mice were performed. All tropolones showed higher LD_50_ and LD_90_, and the median lethal dose of these compounds was between 6 and 10 mg/kg. To evaluate a possible mechanism of action of these tropolones, molecular docking was performed targeting tubulin protein. Based on dock scores, colchicoside and demecolcine presented the highest and the lowest affinity to tubulin, respectively. The best isomers of antitubulin were colchicoside, colchicine and N-deacetyl-N-formyl colchicine. These findings demonstrate that these tropolone alkaloids are promising compounds, especially colchicoside alkaloids, which showed strong results in leishmanicidal effects and docking studies (Azadbakht et al., 2020).

Staurosporine (STS) is an indolocarbazole isolated from *Streptomyces sanyensis*. To evaluate the antileishmania activity and to elucidate a possible mechanism of induced cell death, natural staurosporines (STS, 7OSTS, 4′D4′OSTS, and SCZ B) and its commercial analogs rebeccamycin, K252a, K252b, K252c, and arcyriaflavin A were tested against *L. amazonensis, L. donovani* and *T. cruzi*. The compounds STS and 7OSTS showed the lowest IC_50_ values against promastigotes of *L. amazonensis* (0.08 and 3.6 μM, respectively) and *L. donovani* (2.1 and 0.1 μM, respectively), while 4′D4′OSTS and SCZ B were not active against *L. donovani*, and rebeccamycin K252c and arcyriaflavin were inactive in both parasites. Compound 7OSTS was more active against *L. amazonensis* amastigotes (IC_50_ of 0.1 μM) than was miltefosine. This indolocarbazole induced mitochondrial damage in *L. amazonensis* but not in *L. donovani*, as determined using the IC_90_. However, cytoplasmic membrane permeability in *L. donovani* was induced by 7OSTS but not in *L. amazonensis.* Despite the differences in activity observed during the tests against *L. amazonesis* and *L. donovani*, the natural indolocarbazole 7OSTS exhibited promise (Cartuche et al., 2020).

*Aspidosperma spruceanum* Benth. ex Müll. Arg is a tree of the Apocynaceae family that has medicinal properties and has been used for leishmaniasis treatment in Amazonian regions (Morales-Jadán et al., 2020). Using bioinformatic tools, a new approach to predict new compounds capable of fighting diseases, three indole alkaloids (from *Aspidosperma spruceanum*) were investigated against *Leishmania* targets. The chosen indole alkaloids were aspidocarpine (APC), aspidoalbine (APA) and tubotaiwine (TBT), and *in silico* tests showed that all of them fulfilled Lipinski's rule. Four *Leishmania* species cause leishmaniasis in the region where *Aspidosperma spruceanum* is used: *L. braziliensis*, *L. panamensis*, *L. amazonensis*, and *L. mexicana*. Five targets of *Leishmania* common to all these species were modeled, and 3D structures were determined for the targets, which were dihydrofolate reductase-thymidylate synthase (DHFR-TS), pteridine reductase 1 (PTR1), pyruvate kinase (PK), hypoxanthine-guanine phosphoribosyltransferase (HGPRT), and squalene synthase (SQS) (Morales-Jadán et al., 2020).

Docking simulations showed that all three indole alkaloids can interact strongly with the *Leishmania* targets. As pidoalbine had more affinity for the active site of PTR1 and against *L. panamensis*, it is able to inhibit some of the functional aspects. The alkaloids demonstrated more affinity to *Leishmania* proteins than to human homologs. These results may be useful for guiding future analyses of the leishmanicidal effects of these compounds *in vivo* and elucidating their possible mechanisms of action (Morales-Jadán et al., 2020).

Alkaloids that were presented along with their leishmanicidal activities (IC_50_) are summarized in (Table 6).

**Table 6:** Chemical structure and leishmanicidal activities of alkaloids.

**Class**	**Subclass**	**Compound name and chemical structure**	***Leishmania* species**	**Assay**	**Values**	**References**
Alkaloids	Glicoalkaloids	Solamargine 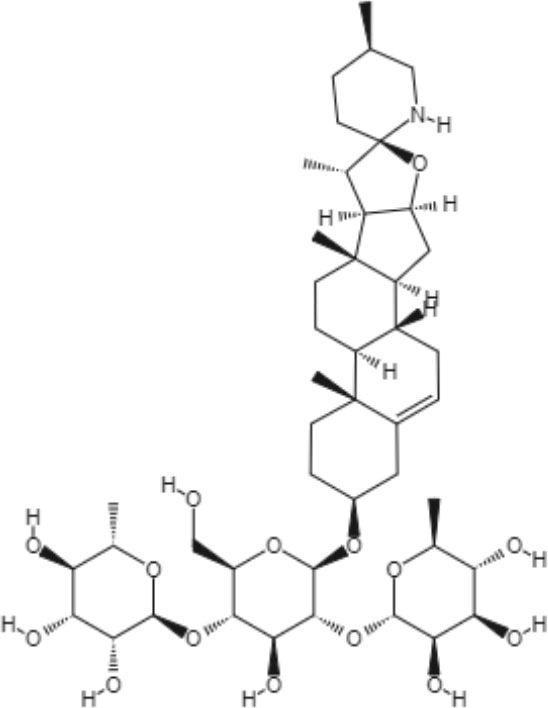	*L. mexicana*	*In vitro*	•**Promastigotes:** IC_50_: 35.1 μM •**Amastigotes (inside BMDM):** IC_50_: 13.4 μM •**Amastigotes (inside BMDDC):** IC_50_: 6.03 μM	Lezama-Dávila et al., 2016
				*In vivo*		
		Solasonine 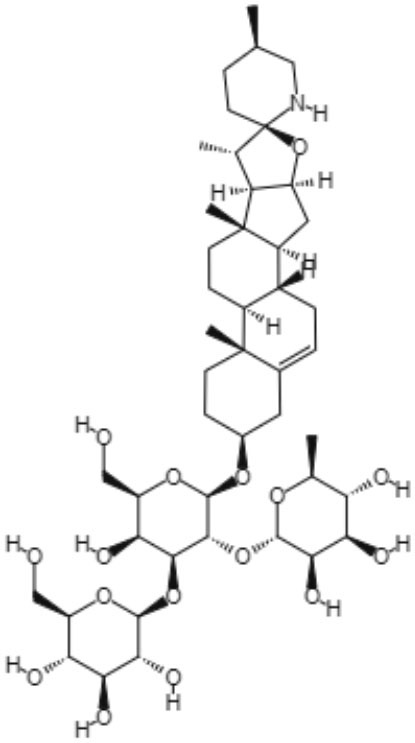		*In vitro*	•**Promastigotes:** IC_50_: 36.5 μM •**Amastigotes (inside BMDM):** IC_50_: 9.3 μM •**Amastigotes (inside BMDDC):** IC_50_: 5.9 μM	Lezama-Dávila et al., 2016
				*In vivo*		
Alkaloids	Piperidines	Piperine (PIP) 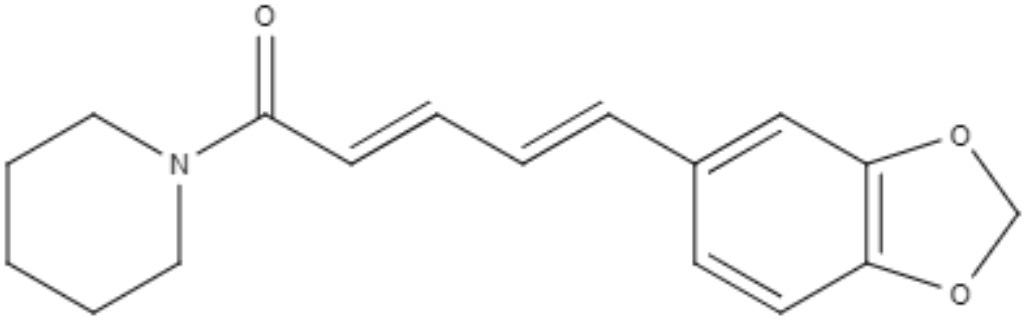	*L. infantum*	*In vitro*	•**Promastigotes:** IC_50_: 3.03 μg/mL •**Axenic amastigotes:** IC_50_: 23.98 μg/mL	Vieira-Araújo et al., 2018
					•**Combination with meglumine antimoniate (compound : meglumine antimoniate):** •**Promastigotes:** IC_50_: 2.1 μg/mL (50% : 50%) •IC_50_: 4.3 μg/mL (25% : 75%) •IC_50_: 4.5 μg/mL (12.5% : 87.5%) •IC_50_: 4.7 μg/mL (6.25% : 93.75%)	
					**Axenic amastigotes:** IC_50_: 18.7 μg/mL (50% : 50%) •IC_50_: 7.3 μg/mL (25% : 75%) •IC_50_: 16.6 μg/mL (12.5% : 87.5%) IC_50_: 19.1 μg/mL (6.25% : 93.75%)	
Alkaloids	Piperidines	Capsaicin (CAP)	*L. infantum*	*In vitro*	•**Promastigotes:** IC_50_: 5.01 μg/mL •**Axenic amastigotes:** IC_50_: 24.2 μg/mL	Vieira-Araújo et al., 2018
		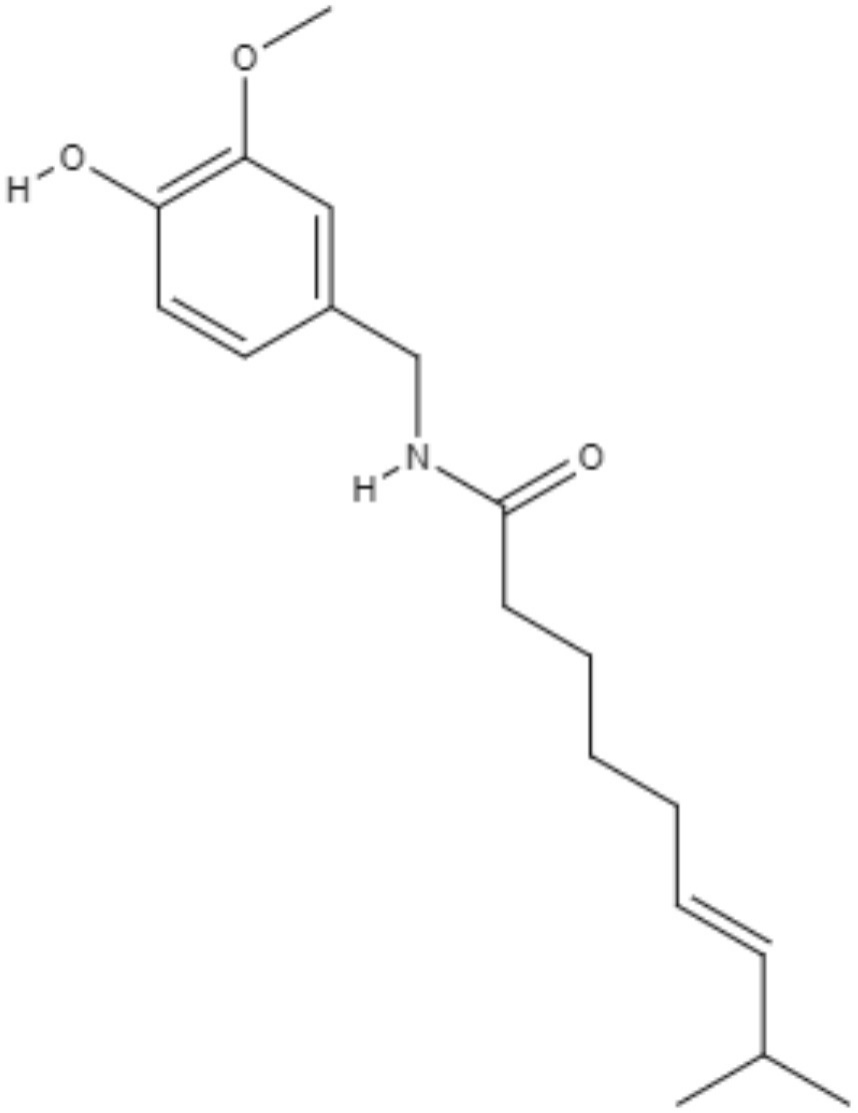				
					**Combination with meglumine antimoniate (compound : meglumine antimoniate):** •**Promastigotes:** IC_50_: 2.9 μg/mL (50% : 50%) •IC_50_: 5.6 μg/mL (25% : 75%) •IC_50_: 5.5 μg/mL (12.5% : 87.5%) •IC_50_: 18.4 μg/mL (6.25% : 93.75%) •**Axenic amastigotes:** IC_50_: 22.3 μg/mL (50% : 50%) •IC_50_: 23.9 μg/mL (25% : 75%) •IC_50_: 28.3 μg/mL (12.5% : 87.5%) •IC_50_: 27.5 μg/mL (6.25% : 93.75)%	
Alkaloids	Piperidines	HDGG-AmB-Pip-NPs^1^	*L. donovani*	*In vitro*	•**Promastigotes:** IC_50_: 21.9 ng/mL •**Intracellular amastigotes:** IC_50_: 4.9 ng/mL	Ray et al., 2020
				*In vivo*		
		Eu-HDGG-AmB-Pip-NPs^2^	*L. donovani*	*In vitro*	•**Promastigotes:** IC_50_: 24.0 ng/mL •**Intracellular amastigotes:** IC_50_: 18.3 ng/mL	Ray et al., 2020
				*In vivo*		
		(-)-Cassine 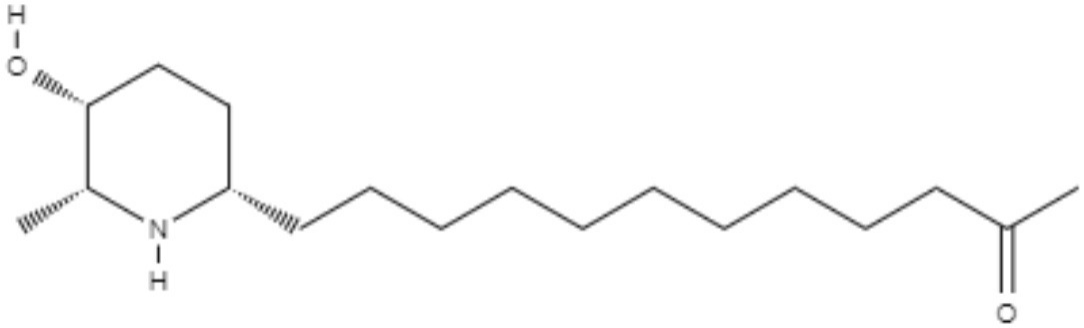	*L. amazonensis*	*In vitro*	**Promastigotes:** IC_50_: 25.2 μg/mL	Lacerda et al., 2018
		(-)-Spectaline 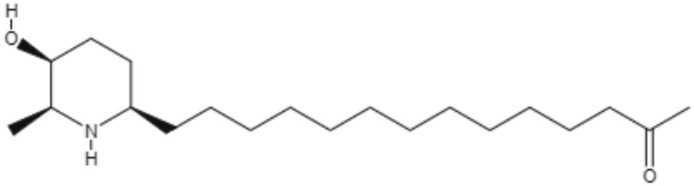			**Promastigotes:** IC_50_: 15.8 μg/mL	
Alkaloids	Piperidines	(-)-3-O-acetylcassine 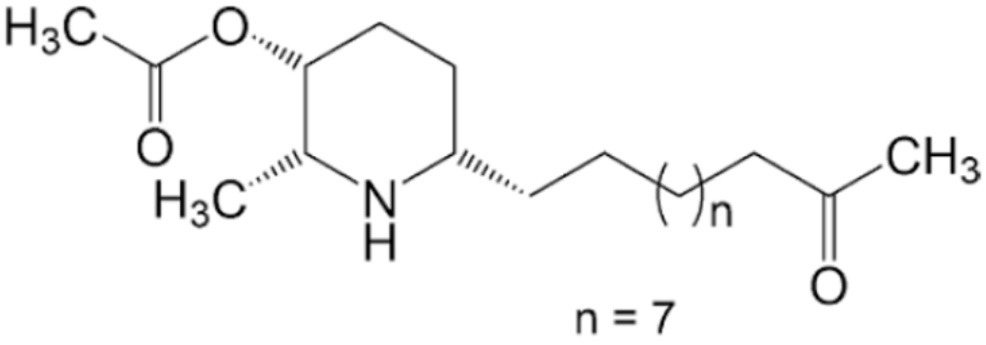	*L. amazonensis*	*In vitro*	**Promastigotes:** IC_50_: 30.3 μg/mL	Lacerda et al., 2018
		(-)-3-O-acetylspectaline 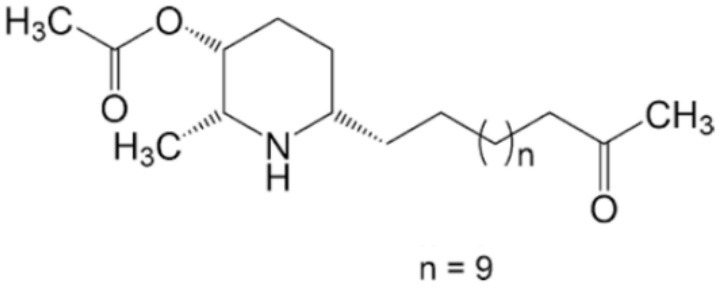			**Promastigotes:** IC_50_: 25.9 μg/mL	
	Isoquinolines	Berberine 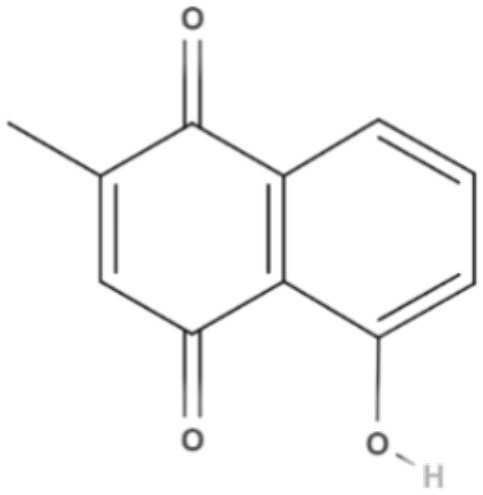	*Leishmania donovani* UR6	*In vitro*	•**Promastigotes:** IC_50_: 4.8 μM •IC_90_: 50.0 μM	De Sarkar et al., 2018
		CS-PEO-Berberine^3^	*L. major*	*In vitro*	•**Promastigotes:** IC_50_: 0.2 μg/mL •**Amastigotes:** IC_50_: 0.9 μg/mL	Rahimi et al., 2020
Alkaloids	Tropolones	Colchicoside 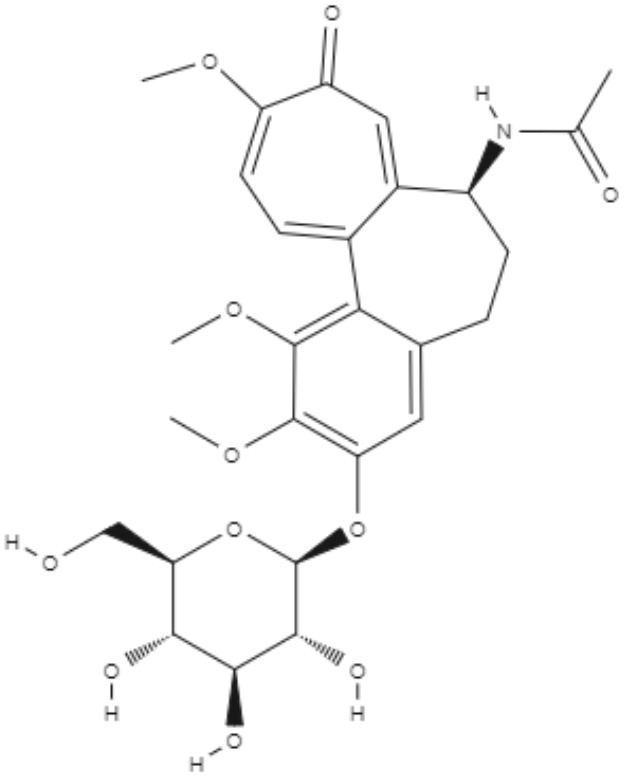	*L. major*	*In vitro*	•**Promastigotes:** IC_50_: 0.2 μg/mL •**Amastigote:** IC_50_: 4.0 μg/mL	Azadbakht et al., 2020
				*In vivo* toxicity assay	•**Brine shrimp test:** LD_50_: 452.8 μg/mL •LD_90_: 1782.7 μg/mL •**Acute toxicity in mice:** LD_50_: 9.1 μg/mL	
		2-Demethyl colchicine 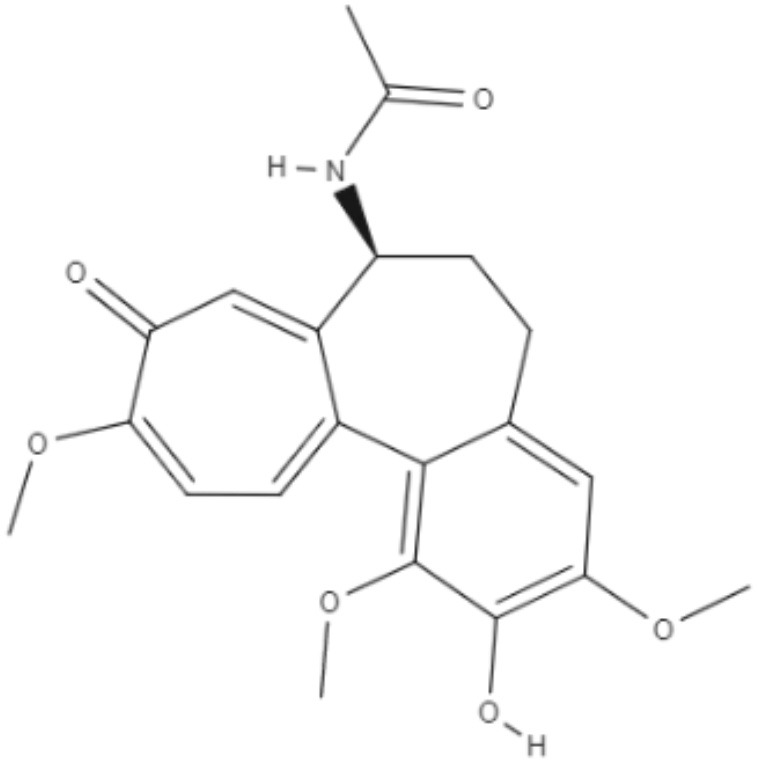		*In vitro*	•**Promastigotes:** IC_50_: 0.5 μg/mL •**Amastigotes:** IC_50_: 10.2 μg/mL	
				*In vivo* toxicity assay	•**Brine shrimp test:** LD_50_: 518.9 μg/mL •LD_90_: 1852.5 μg/mL •**Acute toxicity in mice:** LD_50_: 8.3 μg/mL	
Alkaloids	Tropolones	3-Demethyl colchicine 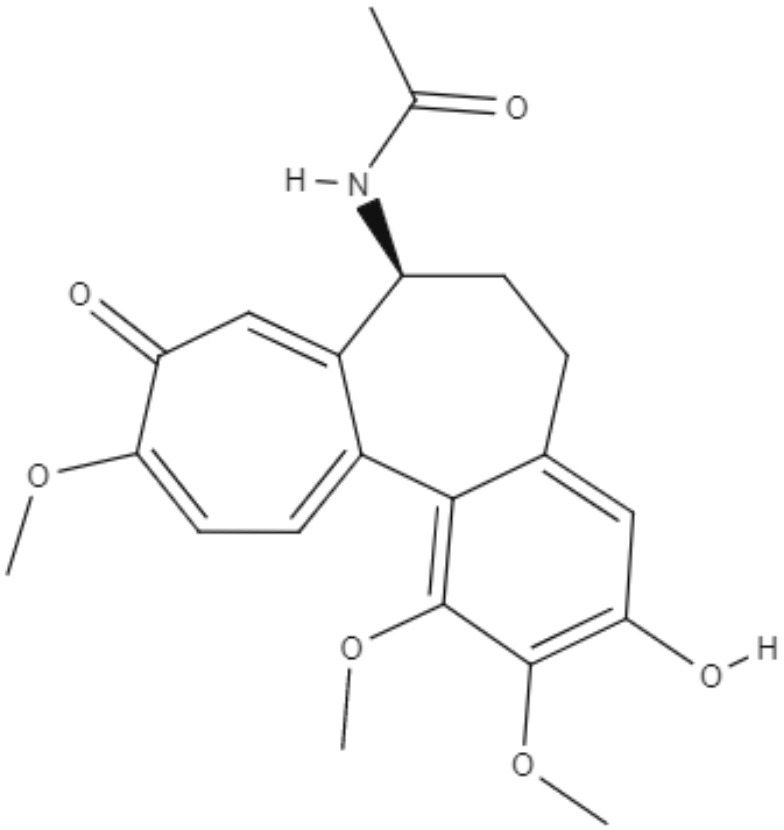	*L. major*	*In vitro*	•**Promastigotes:** IC_50_: 0.4 μg/mL •**Amastigotes:** IC_50_: 11.1 μg/mL	Azadbakht et al., 2020
				*In vivo* toxicity assay	•**Brine shrimp test:** LD_50_: 568.5 μg/mL •LD_90_: 1982.8 μg/mL •**Acute toxicity in mice:** LD_50_: 9.0 μg/mL	
		Demecolcine 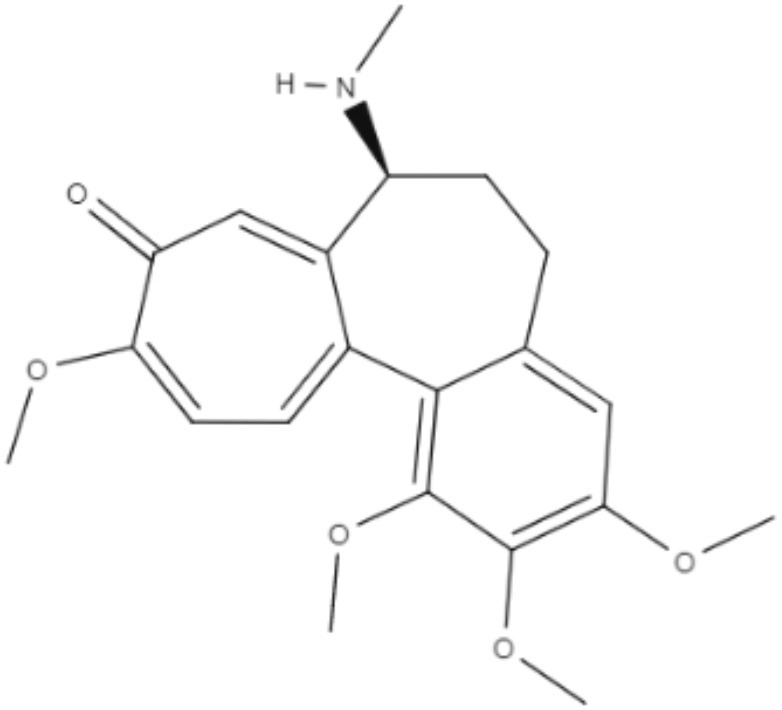		*In vitro*	•**Promastigotes:** IC_50_: 0.7 μg/mL •**Amastigotes:** IC_50_: 14.8 μg/mL	
				*In vivo* toxicity assay	•**Brine shrimp test:** LD_50_: 542.4 μg/mL •LD_90_: 1693.0 μg/mL •**Acute toxicity in mice:** LD_50_: 9.7 μg/mL	
Alkaloids	Tropolones	Colchifoline 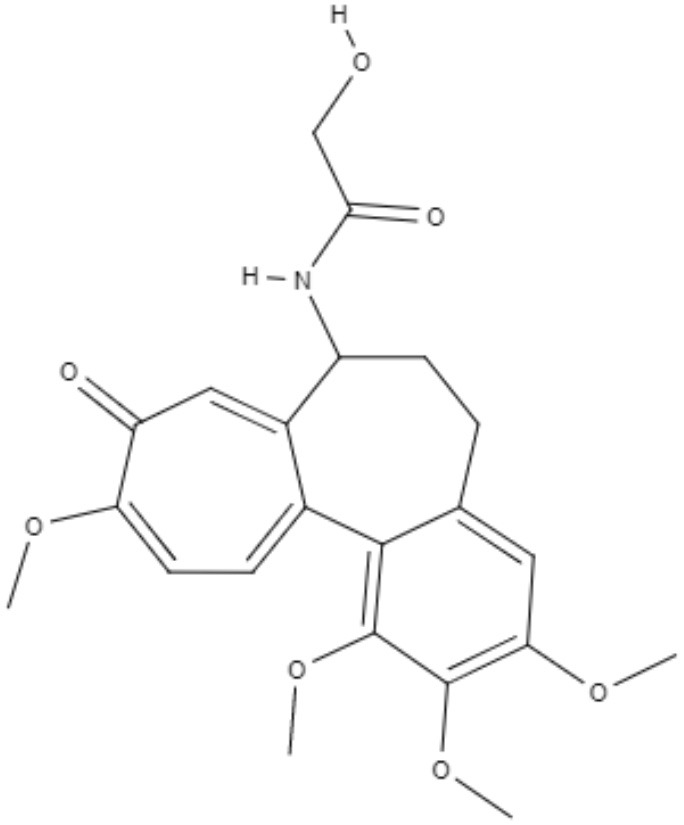	*L. major*	*In vitro*	•**Promastigotes:** IC_50_: 0.7 μg/mL •**Amastigotes:** IC_50_: 14.0 μg/mL	Azadbakht et al., 2020
				*In vivo* toxicity assay	•**Brine shrimp test:** LD_50_: 528.5 μg/mL •LD_90_: 1734.5 μg/mL •**Acute toxicity in mice:** LD_50_: 9.1 μg/mL	
		N-deacetyl-N-formyl colchicine 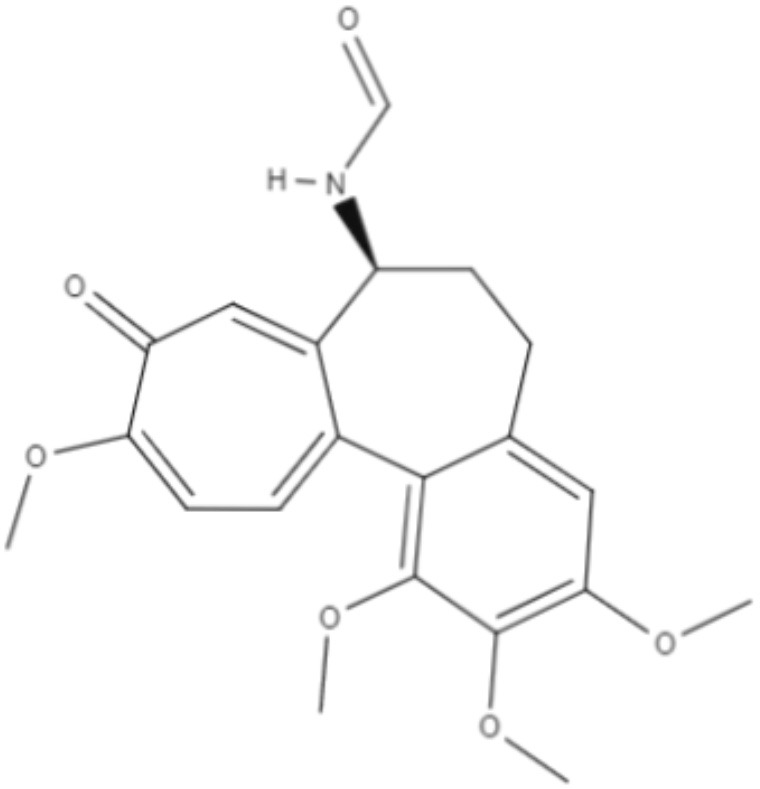		*In vitro*	•**Promastigotes:** IC_50_: 0.5 μg/mL •**Amastigotes:** IC_50_: 10.2 μg/mL	
				*In vivo* toxicity assay	•**Brine shrimp test:** LD_50_: 542.8 μg/mL •LD_90_: 1846.9 μg/mL •**Acute toxicity in mice:** LD_50_: 7.9 μg/mL	
Alkaloids	Tropolones	Colchicine 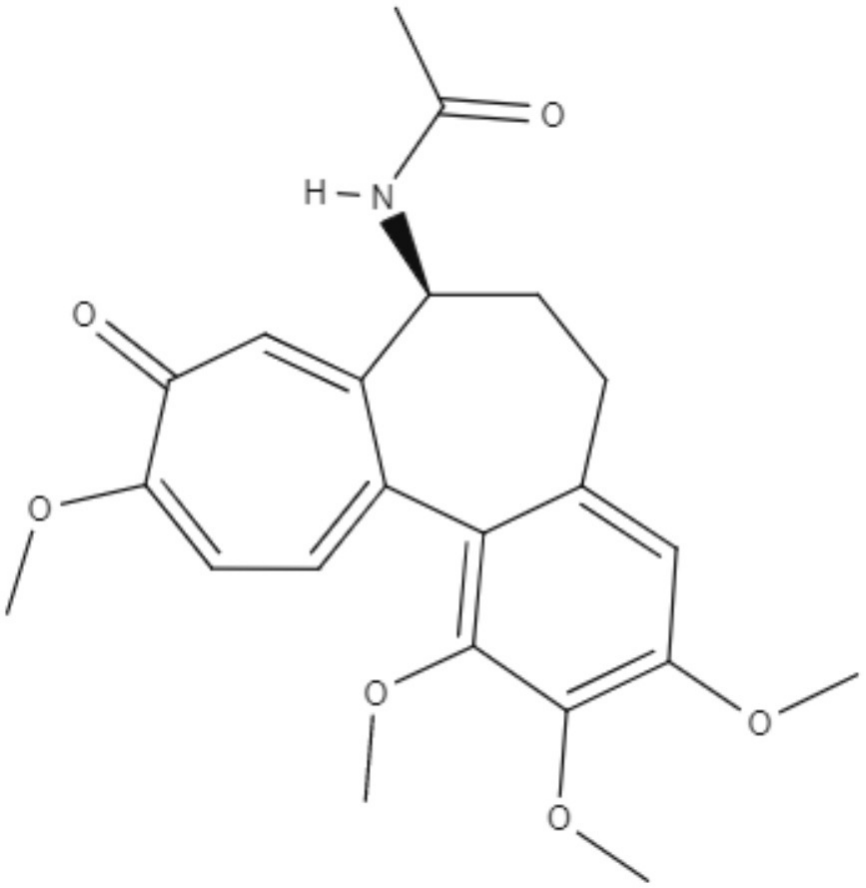	*L. major*	*In vitro*	•**Promastigotes:** IC_50_: 0.4 μg/mL •**Amastigotes:** IC_50_: 8.7 μg/mL	Azadbakht et al., 2020
				*In vivo* toxicity assay	•**Brine shrimp test:** LD_50_: 585.2 μg/mL •LD_90_: 1952.5 μg/mL •**Acute toxicity in mice:** LD_50_: 6.1 μg/mL	
		Cornigerine 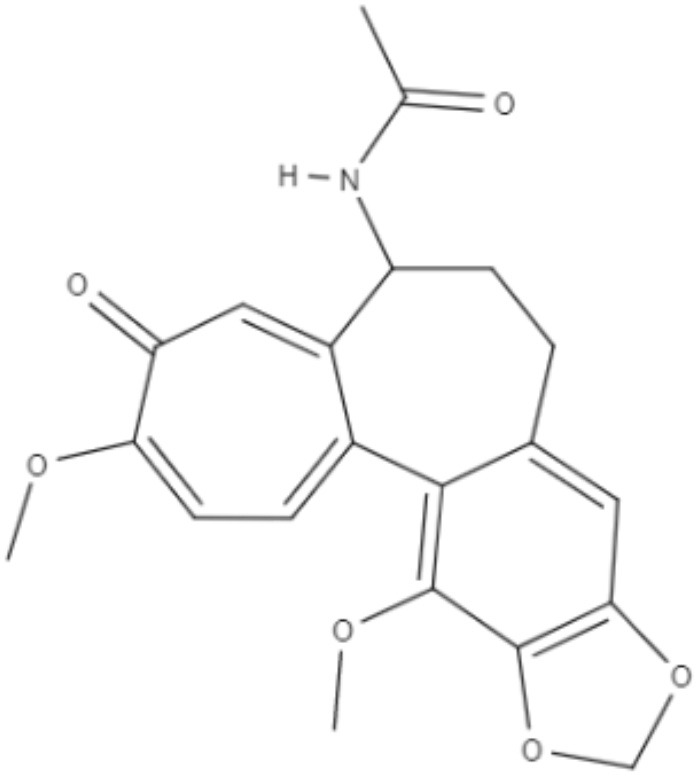		*In vitro*	•**Promastigotes:** IC_50_: 0.8 μg/mL •**Amastigotes:** IC_50_: 11.9 μg/mL	
				*In vivo* toxicity assay	•**Brine shrimp test:** LD_50_: 538.8 μg/mL •LD_90_: 1889.0 μg/mL •**Acute toxicity in mice:** LD_50_: 7.8 μg/mL	
Alkaloids	Indolocarbazoles	Staurosporine (STS) 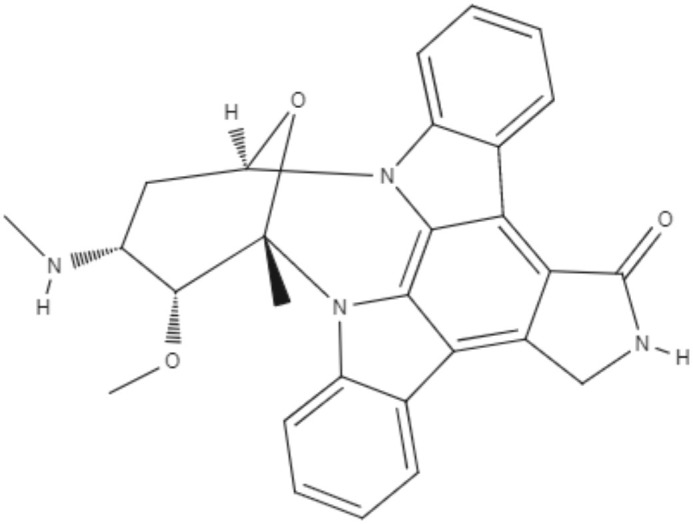	*L. amazonensis*	∙*In vitro*	•**Promastigotes:** IC_50_: 0.08 μM •**Intracelular amastigotes:** IC_50_: 10.0 μM	Cartuche et al., 2020
			*L. donovani*		**Promastigotes:** IC_50_: 2.1 μM	
		7-Oxostaurosporine (7OSTS) 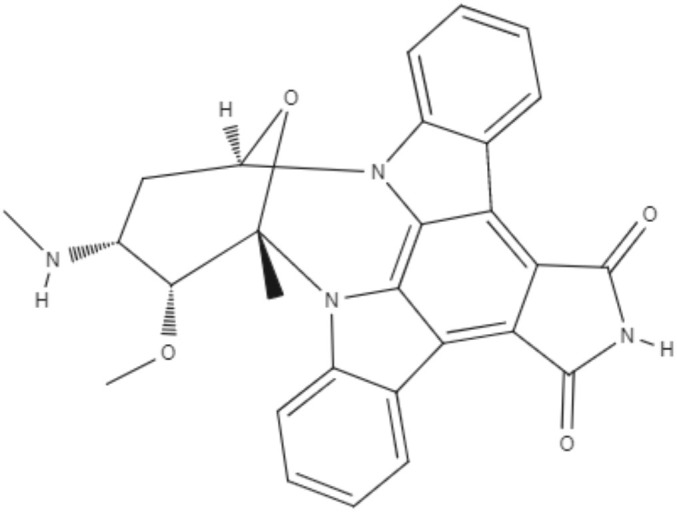	*L. amazonensis*		•**Promastigotes:** IC_50_: 3.6 μM •**Intracelular amastigotes:** IC_50_: 0.1 μM	
			*L. donovani*		**Promastigotes:** IC_50_: 0.6 μM	
Alkaloids	Indolocarbazoles	4'-Demethylamine-4'-oxostaurosporine (4'D4'OSTS) 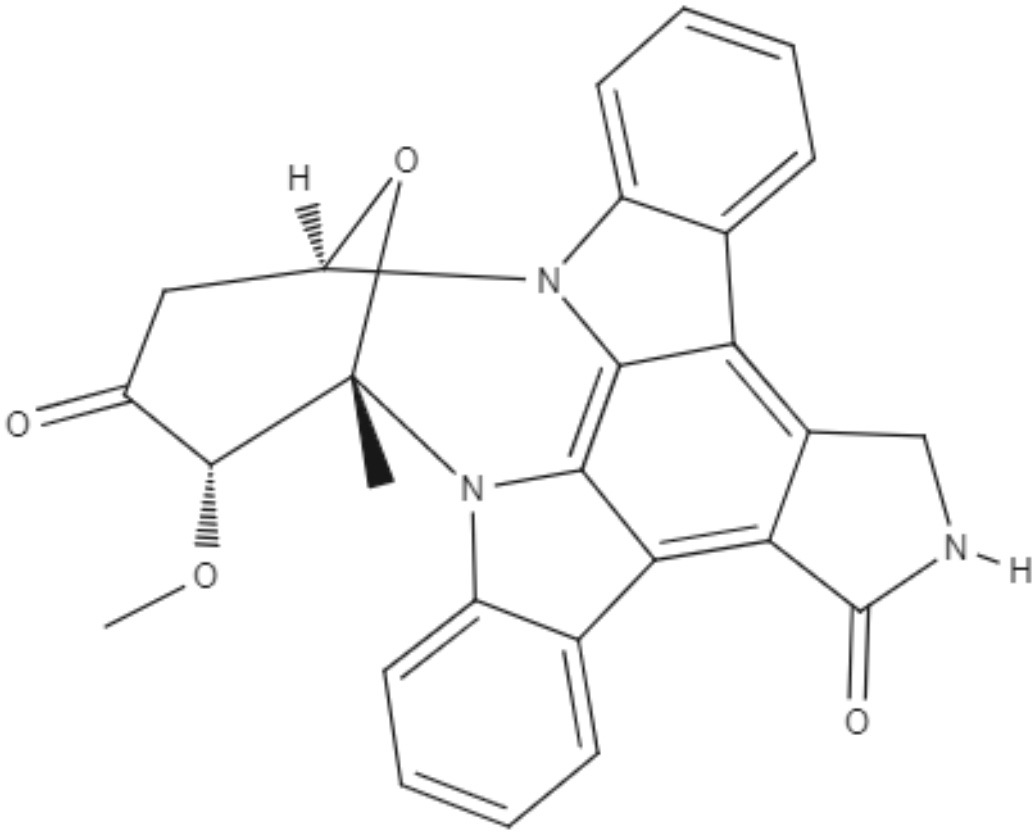	*L. amazonensis*	*In vitro*	•**Promastigotes:** IC_50_: 17.1 μM •**Intracelular amastigotes:** IC_50_: 2.0 μM	Cartuche et al., 2020
			*L. donovani*		**Promastigotes:** IC_50_: > 40 μM	
		Streptocarbazole B (SCZ B) 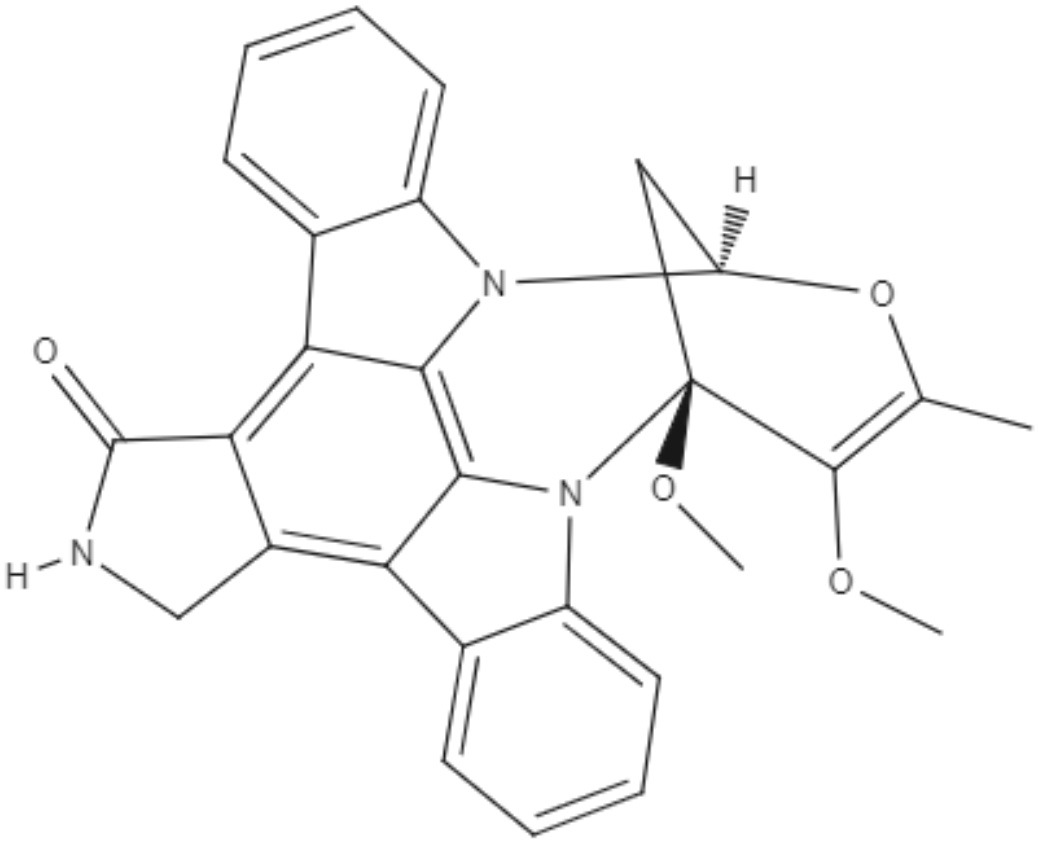	*L. amazonensis*		•**Promastigotes:** IC_50_: 10.4 μM •**Intracelular amastigotes:** IC_50_: 2.5 μM	
			*L. donovani*		**Promastigotes:** IC_50_: > 40 μM	
Alkaloids	Indoles	Aspidocarpine (APC) 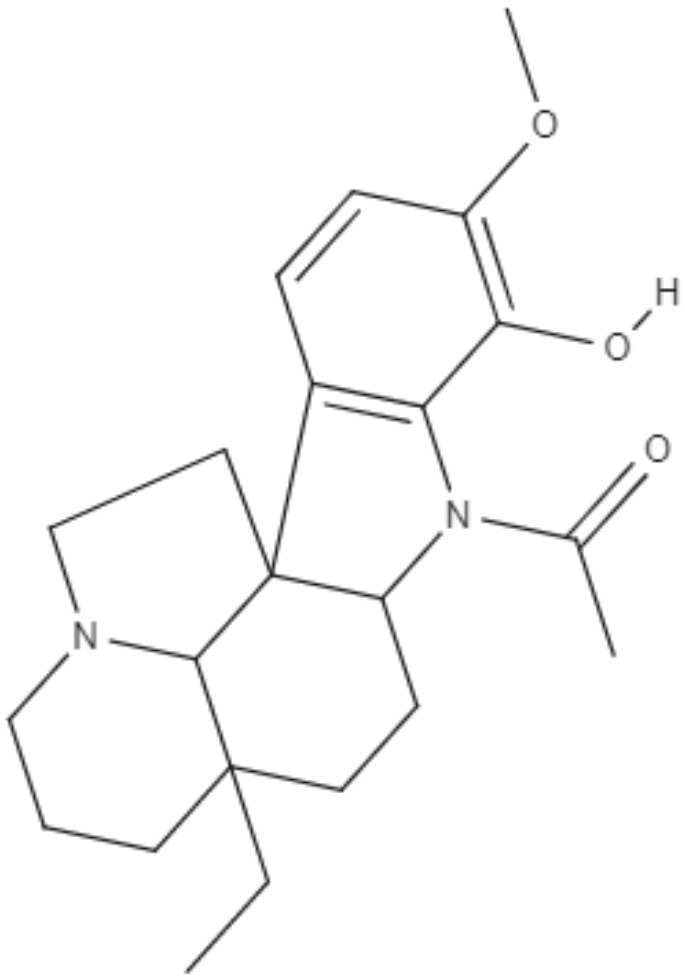	•*L. amazonensis* •*L. braziliensis* •*L. panamensis* •*L. mexicana*	•*In silico* •Docking studies	ND	Morales-Jadán et al., 2020
		Aspidoalbine (APA) 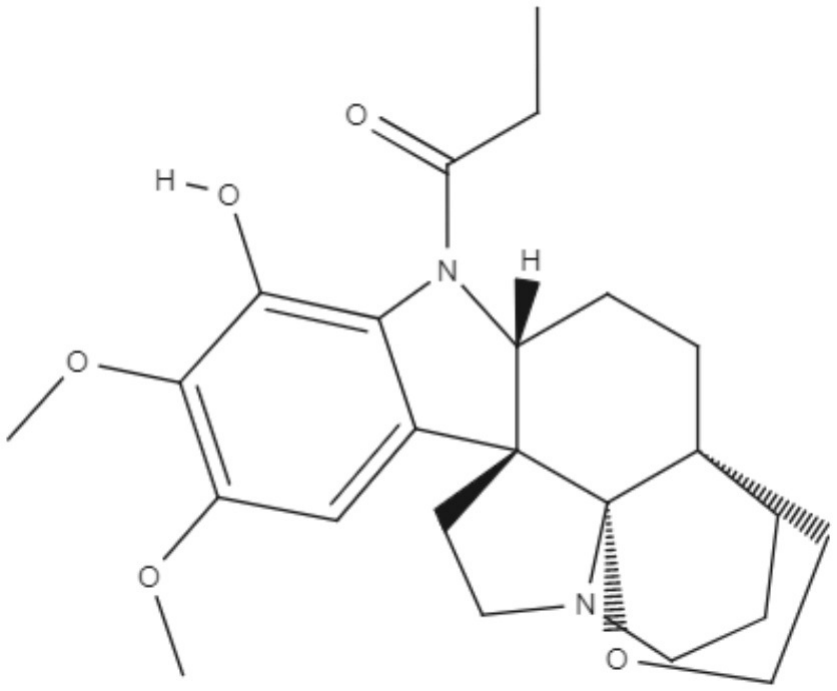			ND	
Alkaloids	Indoles	Tubotaiwine (TBT) 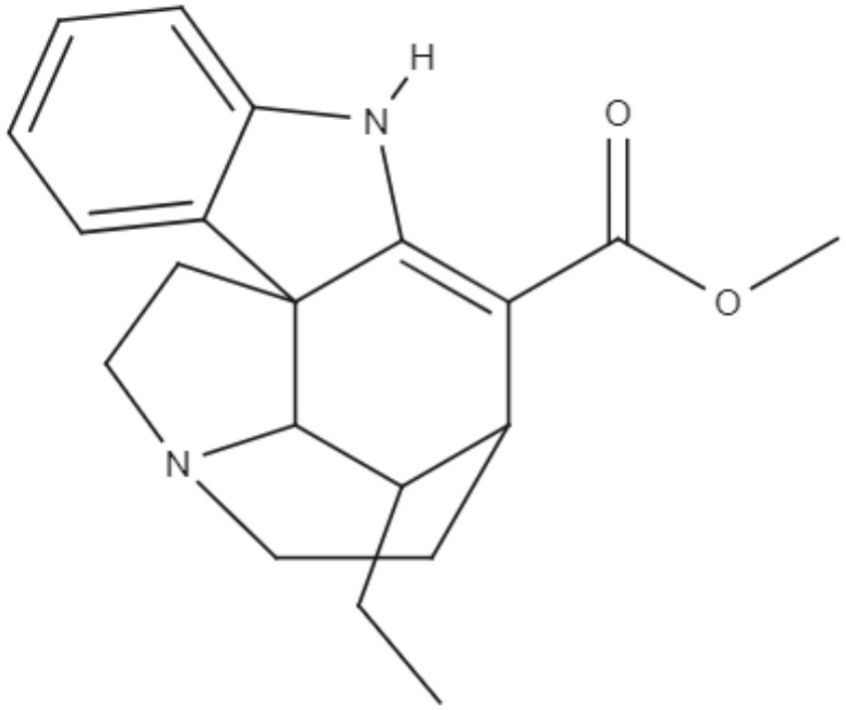	•*L. amazonensis* •*L. braziliensis* •*L. panamensis* •*L. mexicana*	•*In silico* •Docking studies	ND	Morales-Jadán et al., 2020

*^a^Amphotericin B (AmB) and piperine (Pip) entrapped in guar gum (HDGG) nanoparticles (NPs)*.

*^b^Eudragit L30D-coated AmB and Pip-loaded guar gum nanoparticles*.

*^c^Chitosan (CS)-polyethylene oxide (PEO) nanofibers containing berberine*.

*ND, Not demonstrated; BMDM, Bone marrow derived macrophage; BMDDC, Bone marrow derived dendritic cell*.

### Flavonoids

Flavonoids can be defined as a group of metabolites originating from the combination of the skikimate and acetate pathways biosynthesized from cinnamic acid, which has a C6-C3-C6 basic structure and phenylbenzopyran functionality. The product of the first cyclization is chalcone, a precursor of most groups of flavonoids. These natural compounds are divided according to the linkage between the aromatic rings to the benzopyran portion in phenylbenzopyran flavonoids, isoflavonoids and neoflavonoids. Phenylbenzopyrans flavonoids (2-phenylbenzopyrans) are classified as flavan, flavanone, flavone, flavonol, dihydroflavonol, flavan-3-ol, flavan-4-ol and flavan-3,4-diol according to both oxidation and saturation of the heterocyclic C-ring. Isoflavonoids (3-benzopyrans) have a 3-phenylchroman structure with a wide range that can be classified as isoflavan, isoflavone, isoflavanone, isoflav-3-ene, isoflavanol, rotenoid, coumestane, 3-arylcoumarin, coumaronochromene, and pterocarpan. Neoflavonoids (4-benzopyrans) have a structure similar to flavonoids and isoflavonoids and are divided into 4-arylcoumarins, 3,4-dihydro-4-arylcoumarins and neoflavenes. These groups have a variety of biological activities, including against trypanosomes (Winkel, 2006; Schmidt et al., 2012b).

Quercetin, a flavonol extracted from *Kalanchoe pinnata*, demonstrated activity against promastigotes of *L. amazonensis* (IC_50_ value of 31.4 μM after 48 h). This compound increased reactive oxygen species levels, causing mitochondrial damage and leading to the death of the parasite (Fonseca-Silva et al., 2013). Against *L. amazonensis* intracellular amastigotes, quercetin exhibited an IC_50_ value of 3.4 μM and a selectivity index of 16.8 (Fonseca-Silva et al., 2013).

Quercetin was also capable of inhibiting arginase, an important enzyme in leishmanial infections, as a possible target for leishmaniasis chemotherapy (Manjolin et al., 2013). Despite these studies, the mechanism of action of quercetin is still unknown. This compound was also tested *in vivo* in *L. amazonensis*-infected mice, a murine model of cutaneous leishmaniasis. Quercetin was orally administered and reduced lesion size and parasite burden in the infected ear at a dose of 16 mg/kg/day (Muzitano et al., 2009).

Apigenin, an important flavone tested in the last decade (Kashyap et al., 2018), showed IC_50_ values of 23.7 μM and 4.3 μM for promastigotes and the intracellular amastigote form of *L. amazonensis*, respectively. The inhibition of intracellular amastigote growth reached 71% after 72 h at the highest dose tested (12 μM) (Fonseca-Silva et al., 2015).

Apigenin was also tested *in vivo* in the cutaneous form of leishmaniasis. This compound was able to reduce the lesion size and parasitic load compared to the control and the reference (meglumine antimoniate), presenting ED_50_ and ED_90_ values of 0.73 and 1.2 mg/kg, respectively (Fonseca-Silva et al., 2016).

As a possible mechanism of action, it was demonstrated that *L. amazonensis*-infected macrophages treated with apigenin showed an increase in the intracellular reactive oxygen species (ROS) and in the number of double-membrane vesicles and myelin-like membrane inclusions, which are characteristics of the autophagic pathway. Furthermore, fusion between autophagosome-like structures and parasitophorous vacuoles was observed (Fonseca-Silva et al., 2016).

Following new chemotherapy perspectives for leishmaniasis, Emiliano and Almeida-Amaral (2018) tested apigenin in a combination model with miltefosine, which is the first oral drug for leishmaniases. This association was first tested *in vitro* in THP-1-derived macrophages infected with *L. amazonensis* promastigotes. The ∑FIC (fractional inhibitory concentration sum) was 1.61, showing an additive effect. The *in vivo* efficacy of this combination was assessed in a cutaneous murine model with BALB/c mice infected with *L. amazonensis*. Apigenin and miltefosine were tested alone (2 and 8 mg/kg/day, respectively) or in combination, using half of the original doses (1 mg/kg/day + 4 mg/kg/day, respectively). Both compounds alone exhibited their expected effects in reducing lesion size and parasite load. The combination scheme was also able to significantly reduce the lesion size and parasite load compared to the control, achieving reductions of 75 and 95%, respectively. Serological toxicological markers were measured, indicating possible hepatoxicity of miltefosine alone (8 mg/kg/day). However, the combination of these compounds did not show any renal or hepatic toxicity, indicating the combination scheme as a new favorable model for leishmaniasis treatment since it is effective and less toxic (Emiliano and Almeida-Amaral, 2018).

The effects of (–)-epigallocatechin 3-*O*-gallate (EGCG), the most abundant flavanol constituent of green tea (*Camellia sinensis* (L.) Kuntze; Theaceae) has been tested in a murine model of cutaneous leishmaniasis against promastigotes and intracellular amastigotes of *L. amazonensis* and *L. braziliensis*. Against *L. amazonensis* intracellular amastigotes, EGCG demonstrated an IC_50_ value of 1.6 μM with a selectivity index of 129.4 (Inacio et al., 2013). When tested against *L. braziliensis*, EGCG demonstrated an IC_50_ value of 278.8 μM for promastigotes and 3.4 μM for amastigotes with a selectivity index of 149.5 (Inacio et al., 2014). As a possible mechanism of action, EGCG increased the ROS levels, which led to a decrease in the mitochondrial membrane potential and a decrease in the ATP levels. EGCG was also tested against *L. infantum*-infected macrophages that exhibited an EC_50_ of 2.6 μM (Inacio et al., 2019).

*In vivo*, EGCG was tested against a murine model of cutaneous leishmaniasis using *L. amazonensis* (Inacio et al., 2013) and *L. braziliensis* (Inacio et al., 2014) and a murine model of visceral leishmaniasis using *L. infantum* (Inacio et al., 2019). In cutaneous leishmaniasis, EGCG was able to reduce the lesion size and the parasitic load without serological toxicology. A similar effect was shown in visceral leishmaniasis; EGCG was capable of reducing the liver parasite load, presenting ED_50_ and ED_90_ values of 12.4 and 21.5 mg/kg/day, respectively.

It is well-known that antimonial resistance is a current problem in leishmaniasis chemotherapy. In an attempt to promote a new strategy for an old problem, flavonoids have been tested as a possible alternative for the treatment of antimonial-resistant leishmaniasis. 2′-Hydroxyflavanone (2HF), a flavanone commonly found in citric fruits, was able to reduce the infection index in BALB/c macrophages infected with wild-type or antimony-resistant *L. amazonensis* promastigotes with IC_50_ values of 3.09 and 3.36 μM, respectively. After *in silico* analysis suggested that 2HF was a safe oral compound, the *in vivo* assay was performed. BALB/c mice were infected with wild-type or antimony-resistant *L. amazonensis* promastigotes and treated with 2HF (50 mg/kg/day). 2HF was capable of reducing the lesion size and parasite load compared to untreated and meglumine antimoniate-treated groups in both wild-type and antimony-resistant infections with no hematological or toxicological alterations (Gervazoni et al., 2018).

Several compounds isolated from leaves of *Piper rusbyi* were tested against three species of *Leishmania*. Among all the compounds tested, Flavokavain B, a chalcone, demonstrated good results against *Leishmania.* The IC_50_ value was 11.2 μM against *L. amazonensis*, *L. donovani*, and *L. braziliensis*, which was more effective than pentamidine. Flavokavain B was also tested *in vivo* against *L. amazonensis* infection in the footpads of BALB/c mice. With a 5 mg/kg/day subcutaneous dose, flavokavain B exhibited the best results among those tested, reducing the lesion size and being effective *in vivo* (Flores et al., 2007).

The potential activity of two biflavonoids isolated from *Selaginella sellowii*, amentoflavone and robustoflavone, was investigated against the intracellular amastigote of *L. amazonensis*. The IC_50_ values of amentoflavone and robustoflavone were 0.2 and 5.3 μM, respectively. In addition, the production of NO decreased in the *L. amazonensis*-infected peritoneal macrophages treated with amentoflavone, while treatment with robustaflavone increased the production of NO (Rizk et al., 2014).

The genus *Mimulus* is native to California in North America. Four C-geranyl flavones (diplacone, 3′-O-methyldiplacone, yellow oil, and 3′-O-methyldiplacol) and one geranylated flavone (cannflavin A) were isolated from *Mimulus bigelovii*. The IC_50_ value was determined to be 7.5 μg/mL for both 3′-O-methyldiplacone and yellow oil against axenic amastigotes. 3′-O-Methyldiplacol obtained an IC_50_ value of 7.2 μg/mL, and cannflavin A obtained an IC_50_ value of 14.6 μg/mL, both of which were also tested against axenic amastigotes (Salem et al., 2011).

*Maclura tiinctoria* from the *Moraceae* family is a plant found in tropical countries worldwide, and its extracts are rich in flavonoids. 5,7,3′,4′-Tetrahydroxy-6,8-diprenylisoflavone (CMt), an isolated flavonoid from *M. tiinctoria* leaf extract, was tested against promastigotes and axenic amastigotes from *L. amazonensis* and *L. infantum*. CMt demonstrated IC_50_ values of 2.7, 6.4, 1.1, and 2.5 μg/mL for promastigote and axenic amastigotes of *L. amazonensis* and *L. infantum*, respectively. For both *Leishmania* species, 5,7,3′,4′-tetrahydroxy-6,8-diprenylisoflavone presented a selectivity index over 180. As a possible mechanism of action, CMt caused disturbances in membrane integrity and membrane potential, including increases in ROS production. In the *in vivo* model for visceral leishmaniasis, 5,7,3′,4′-tetrahydroxy-6,8-diprenylisoflavone and a new formulation, such as 5,7,3′,4′-tetrahydroxy-6,8-diprenylisoflavone-containing micelles (CMt/Mic), were able to reduce the parasite load in selected organs (liver, spleen, lymph node, and bone marrow) compared to the control groups and miltefosine-treated group. Cytokine analysis indicated a Th1-type response for 5,7,3′,4′-tetrahydroxy-6,8-diprenylisoflavone and CMt/Mic treatment as most promising (Pereira et al., 2020).

Brachydin A, brachydin B, and brachydin C, three dimeric flavonoids from *Arrabidaea brachypoda*, were evaluated against *L. amazonensis, L. braziliensis*, and *L. infantum* promastigotes. The most promising results were obtained for brachydin B and brachydin C, showing IC_50_ values of 7.05 and 8.8 μM for *L. braziliensis* and IC_50_ values of 9.16 and 10 μM for *L. amazonensis*. *L. amazonensis* was chosen for the antiamastigote assay, and only brachydin B and brachydin C were tested, exhibiting IC_50_ values of 2.2 and 6.25 μM, respectively It is interesting to highlight the only structural difference between brachydin A, brachydin B, and brachydin C as substitutes for the C-ring as a methoxyl group for brachydin B and a hydroxyl group for brachydin A. Methoxyl is known to improve membrane permeability, which can explain the best results observed with brachydin B (Rocha et al., 2018).

To investigate possible macrophage and amastigote alterations induced by brachydin B, transmission electron microscopy was performed. Brachydin B did not generate macrophage toxicity, even at higher concentrations (20 and 50 μM). However, for amastigotes, brachydin B induced enlargement of the Golgi apparatus, vesicle accumulation and cytoplasmic disorganization with consequent cell death (Rocha et al., 2018).

Brachydin B was administered *in vivo* in *L. amazonensis*-infected mice using two different routes: topical treatment (1% brachydin B) and oral treatment (25 and 50 mg/kg/day). The topical treatment, as well as oral treatment at 25 mg/kg/day, were not able to reduce lesion size compared to the control group. Oral treatment with 50 mg/kg/day reduced the lesion size 1 week after the start of treatment but showed no significant difference between the control group at the end of the treatment (Rocha et al., 2018).

Tryparedoxin peroxidase (Txnpx) and Trypanothione reductase (TryR) are two essential proteins for leishmania survival for their role in parasite redox metabolism. Therefore, these proteins have been considered good targets for the development of new leishmaniasis treatments (Kumar et al., 2017).

Molecular docking analysis was performed for several compounds, including such flavonoids as taxifolin, kaempeferol, quercetin, and epigalloctechin-3-gallate. Quercetin and taxifolin demonstrated the highest binding energy with Txnpx. The molecular docking study also indicated that the Lys136 residue is an essential ligand that is critical for the interactions (Gundampati et al., 2014; Kumar et al., 2017).

Molecular docking was also performed using gp63, a metalloprotease found on both promastigote and amastigote surfaces, which is essential for parasite virulence and pathogenesis. This enzyme is considered a good target for leishmaniasis new treatment development. Lanaroflavone, podocarpusflavone A, amentoflavone, and podocarpusflavone B, which are known biflavonoids, had the most significant interactions in a molecular docking study performed for *L. major* and *L. panamensis* gp63. These four flavonoids demonstrated the same pattern of interactions for both *Leishmania* species. Lanaroflavone was the most promising compound of all considering binding affinity (Mercado-Camargo et al., 2020).

The flavonoids present in this section with a defined IC_50_ are summarized in the Table 7.

**Table 7:** Chemical structure and leishmanicidal activities of flavonoids.

**Class**	**Subclass**	**Compound name and chemical structure**	***Leishmania species***	**Assay**	**Values**	**References**
Flavonoids	**Flavonol**	**Quercetin** 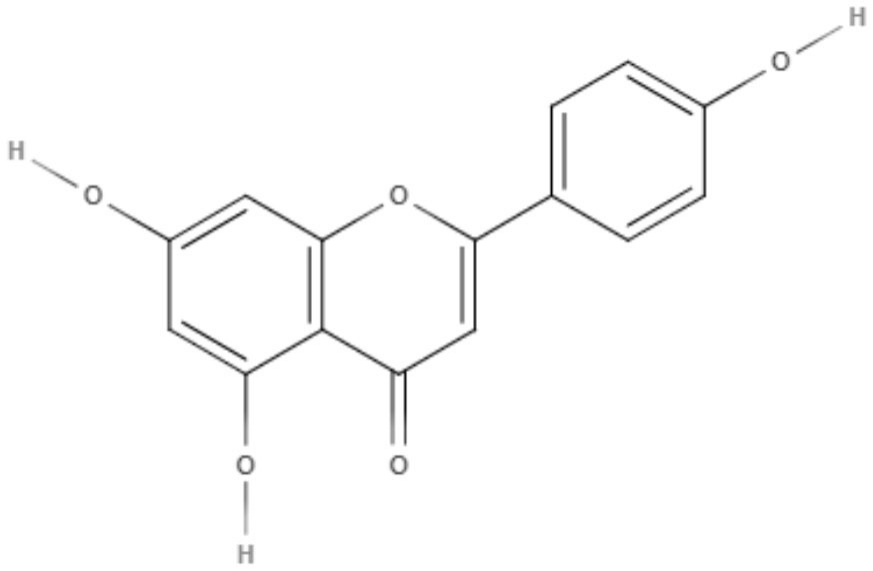	*L. amazonensis*	*In vitro*	**Promastigotes:** IC_50_: 31.4 μM **Intracellular amastigotes:** IC_50_: 3.4 μM	Fonseca-Silva et al., 2013
				*In vivo*	ND	Muzitano et al., 2009
			*L. tropica*	*In vitro*	**Promastigotes:** IC_50_: 182.3 μg/mL **Intracellular amastigotes:** IC_50_: 137.4 μg/mL	Mehwish et al., 2019
	Flavone	**Apigenin** 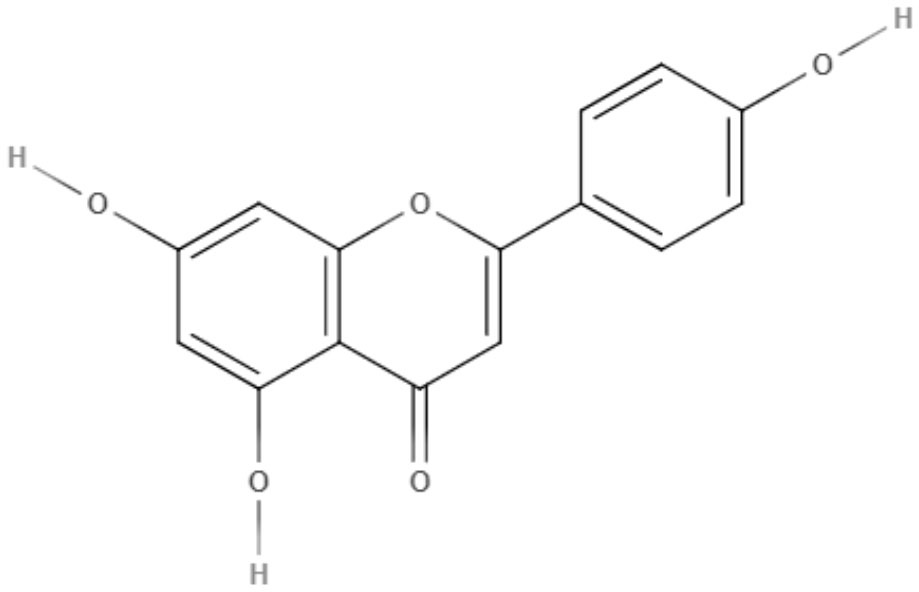	*L. amazonensis*	*In vitro*	**Promastigotes:** IC_50_: 23.7 μM **Intracellular amastigotes:** IC_50_: 4.3 μM	Fonseca-Silva et al., 2015
				*In vivo*	ND	Fonseca-Silva et al., 2016; Emiliano and Almeida-Amaral, 2018
Flavonoids	Flavone	**Amentoflavone** 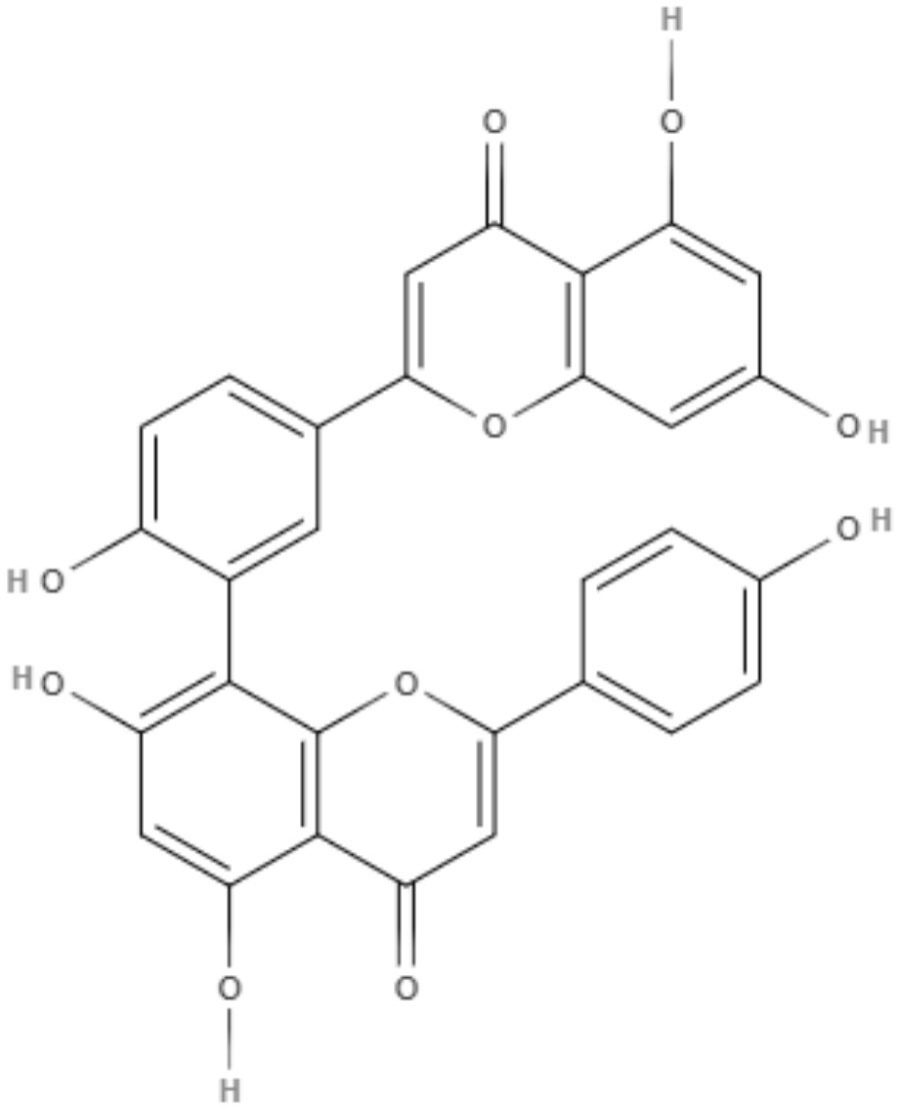	*L. amazonensis*	*In vitro*	**Intracellular amastigotes:** IC_50_: 0.2 μM	Rizk et al., 2014
		**Robustoflavone** 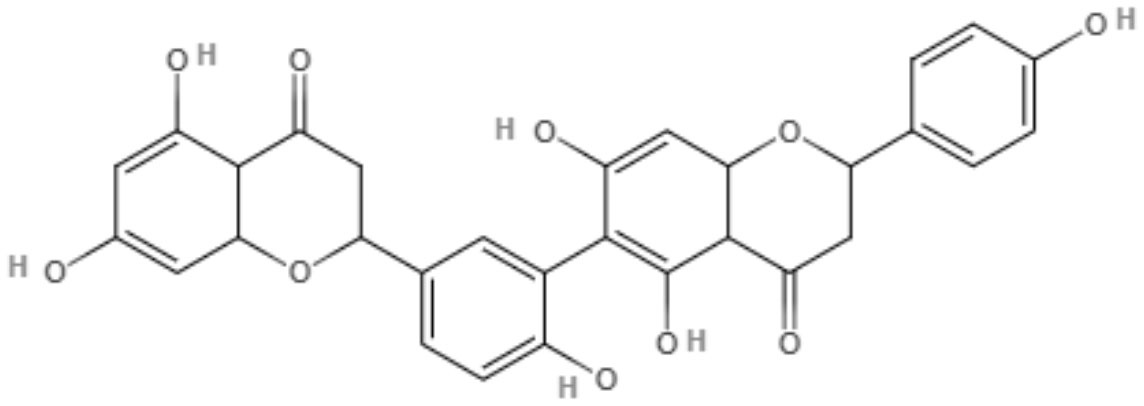			**Intracellular amastigotes:** IC_50_: 5.3 μM	
Flavonoids	Flavone	**Diplacone** 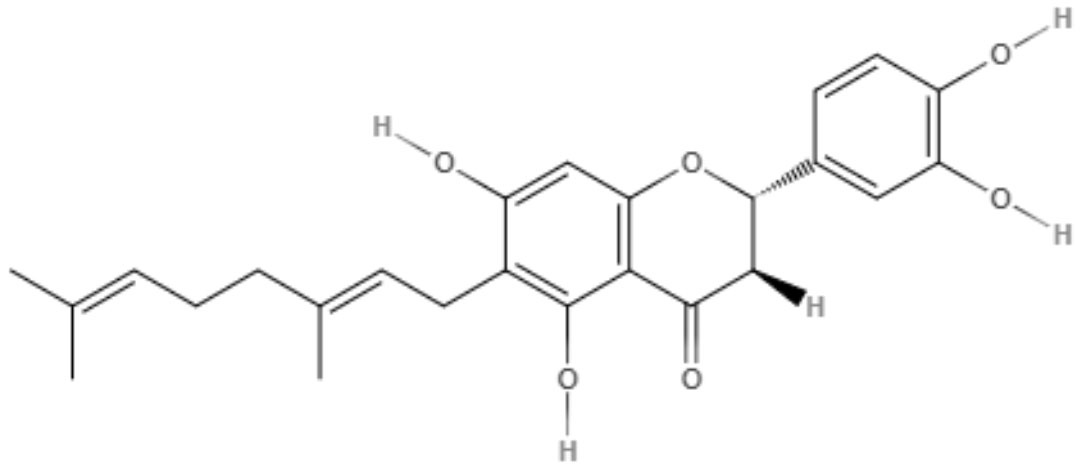	*L. donovani*	*In vitro*	**Axenic amastigotes:** IC_50_: 4.8 μg/mL	Salem et al., 2011
		**3′-O-methyldiplacone** 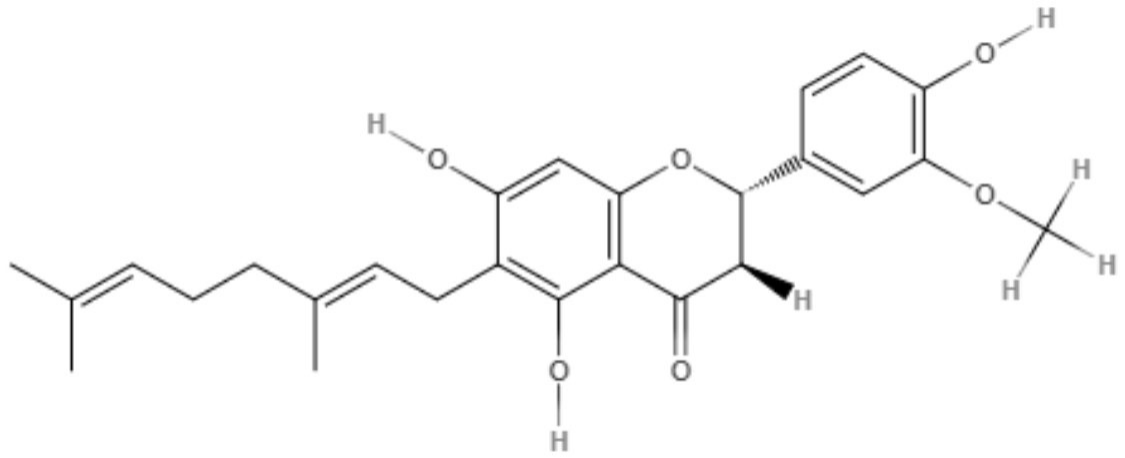			**Axenic amastigotes:** IC_50_: 7.5 μg/mL	
		**4′-O-methyldiplacona** 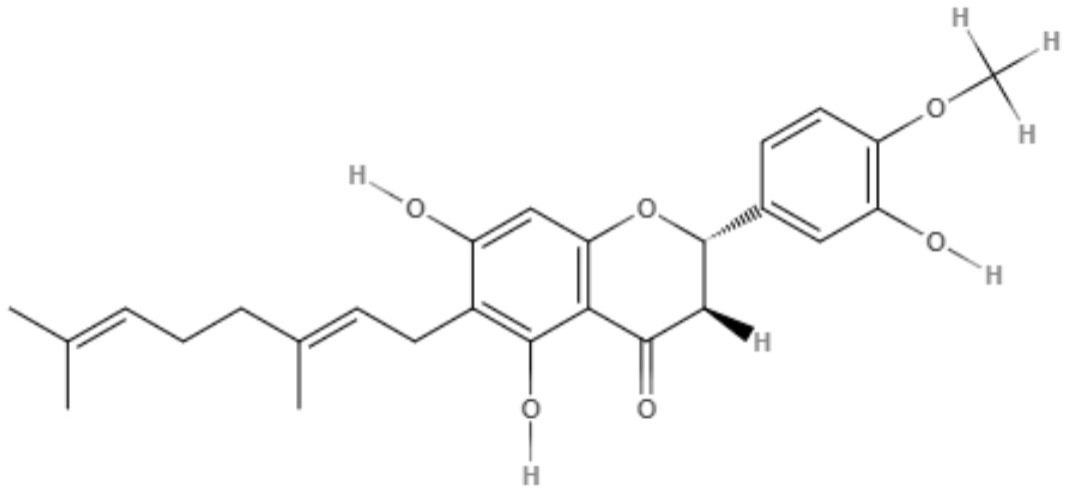			**Axenic amastigotes:** IC_50_: 7.5 μg/mL	
Flavonoids	Flavone	**3′-O-methyldiplacol** 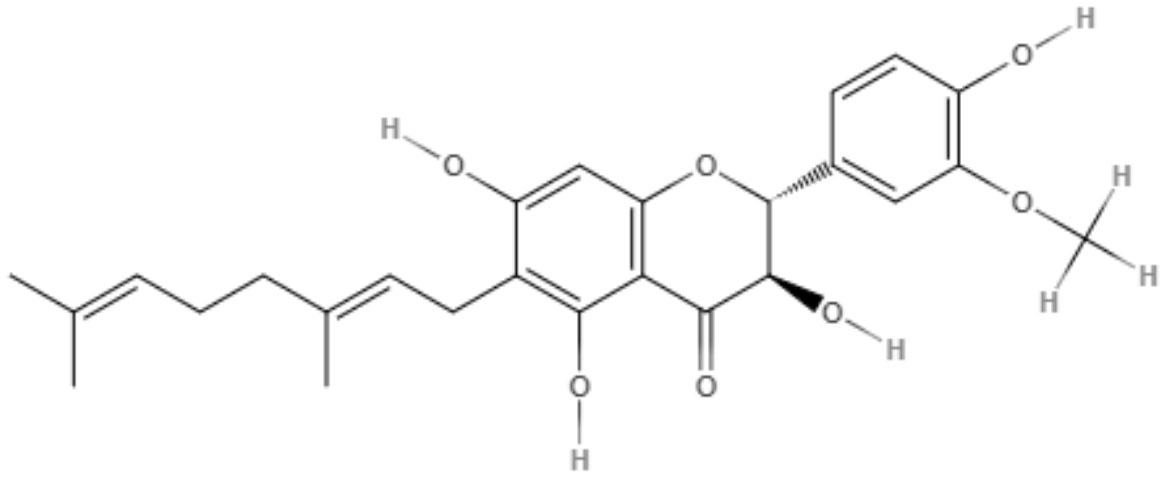	*L. donovani*	*In vitro*	**Axenic amastigotes:** IC_50_: 7.2 μg/mL	Salem et al., 2011
		**Cannflavin A** 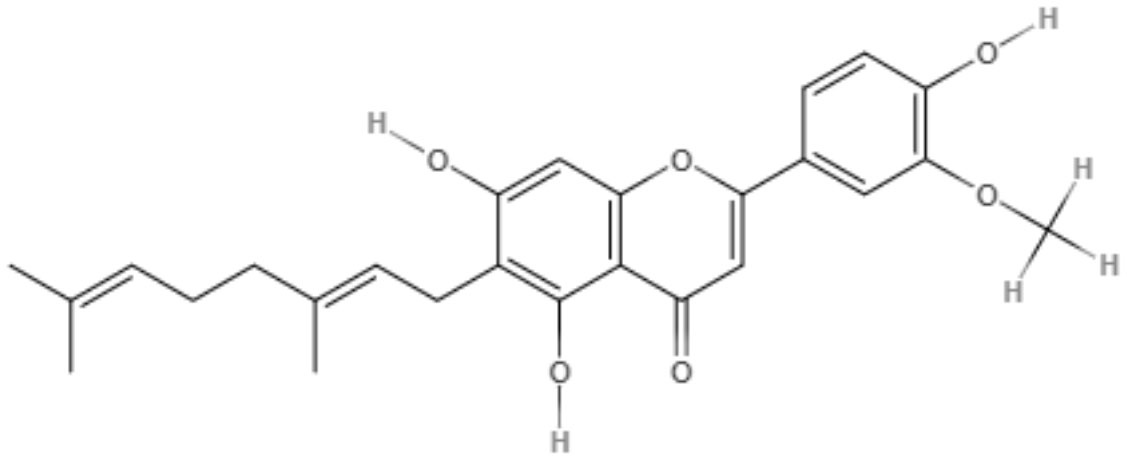			**Axenic amastigotes:** IC_50_: 14.6 μg/mL	
Flavonoids	Flavanol	**(–)-Epigallocatechin 3-O-gallate (EGCG)** 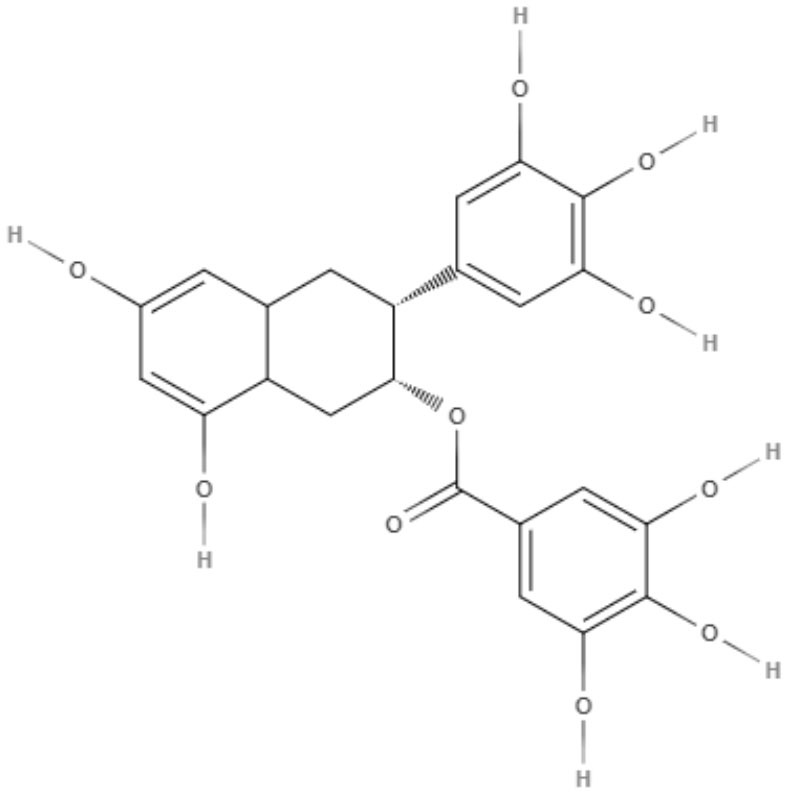	*L. amazonensis*	*In vitro*	**Intracellular amastigotes:** IC_50_: 1.6 μM	Inacio et al., 2013
				*In vivo*	ND	
			*L. braziliensis*	*In vitro*	**Promastigotes:** IC_50_: 278.8 μM **Intracellular amastigotes:** IC_50_: 3.4 μM	Inacio et al., 2014
				*In vivo*	ND	
			*L. infantum*	*In vitro*	**Intracellular amastigotes:** IC_50_: 2.6 μM	Inacio et al., 2019
				*In vivo*	ED_50_: 12.4 mg/kg ED_90_: 21.5 mg/kg	
	Flavanone	**2′-hydroxyflavanone** 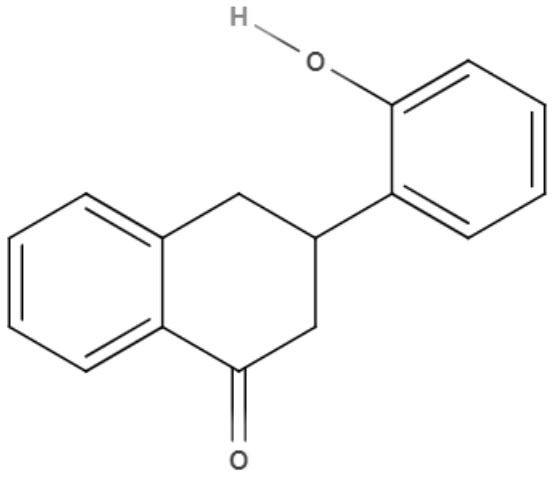	*L. amazonensis*	*In vitro*	**Promastigotes:** IC_50_: 20.51 μM **Intracellular amastigotes:** IC_50_: 3.09 μM **Antimony-resistant** **L. amazonensis** IC_50_: 3.36 μM	Gervazoni et al., 2018
				*In vivo*	ND	
Flavonoids	Chalcone	**Flavokavain B** 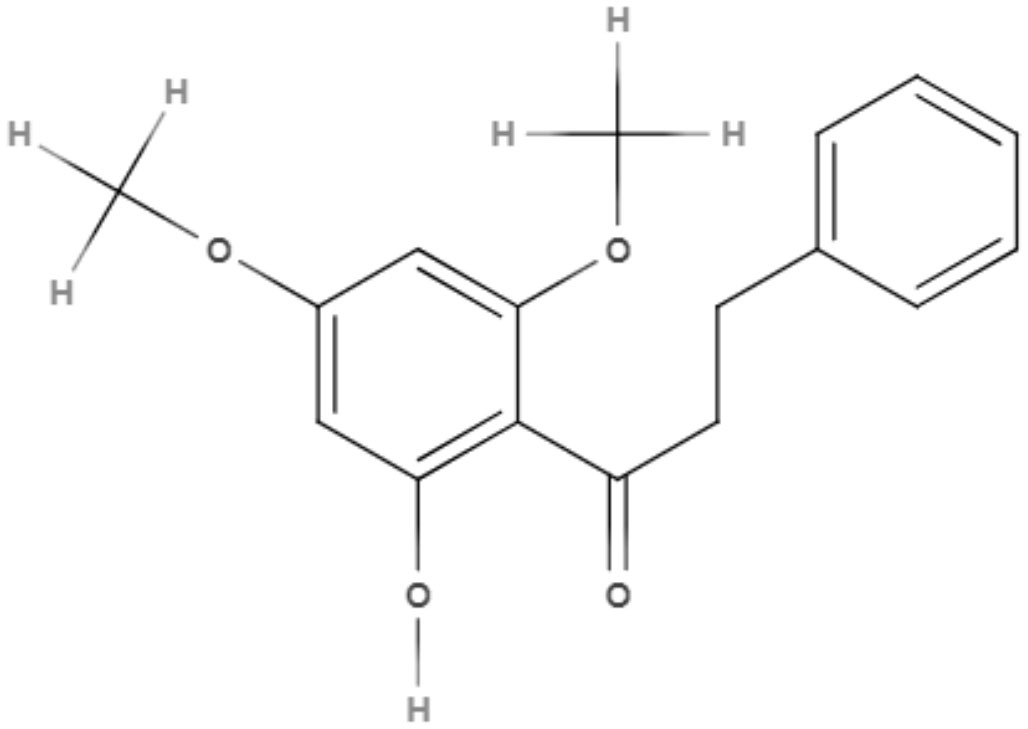	*L. amazonensis*	*In vitro*	**Promastigotes:** IC_50_: 11.2 μM	Flores et al., 2007
				*In vivo*	ND	
			*L. braziliensis*	*In vitro*	**Promastigotes:** IC_50_: 11.2 μM	
			*L. donovani*	*In vitro*	**Promastigotes:** IC_50_: 11.2 μM	
	Isoflavone	**5,7,3′,4′-tetrahydroxy-6,8-diprenylisoflavone (CMt)** 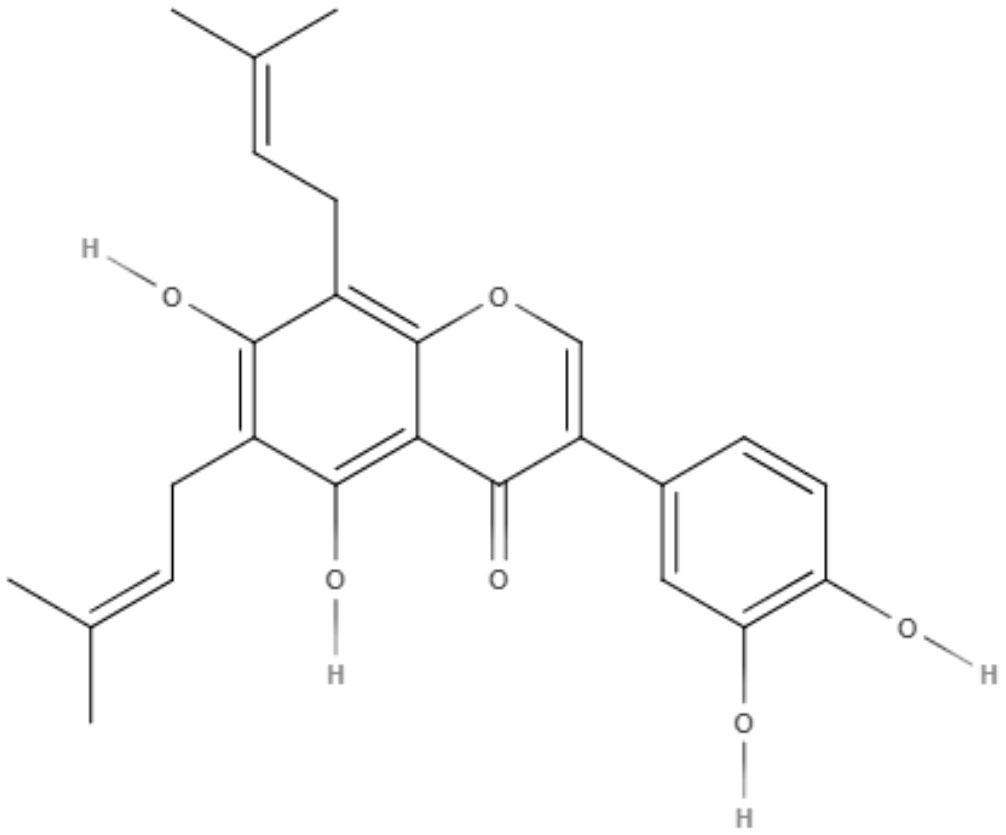	*L. amazonensis*	*In vitro*	**Promastigotes:** IC_50_: 2.7 μg/mL **Axenic amastigotes:** IC_50_: 1.1 μg/mL	Pereira et al., 2020
			*L. infantum*	*In vitro*	**Promastigotes:** IC_50_: 6.4 μg/mL **Axenic amastigotes:** IC_50_: 2.5 μg/mL	
				*In vivo*	ND	
		**CMt-Mic^a^**	*L. infantum*	*In vivo*	ND	
Flavonoids	Biflavonoid	**Brachydin B** 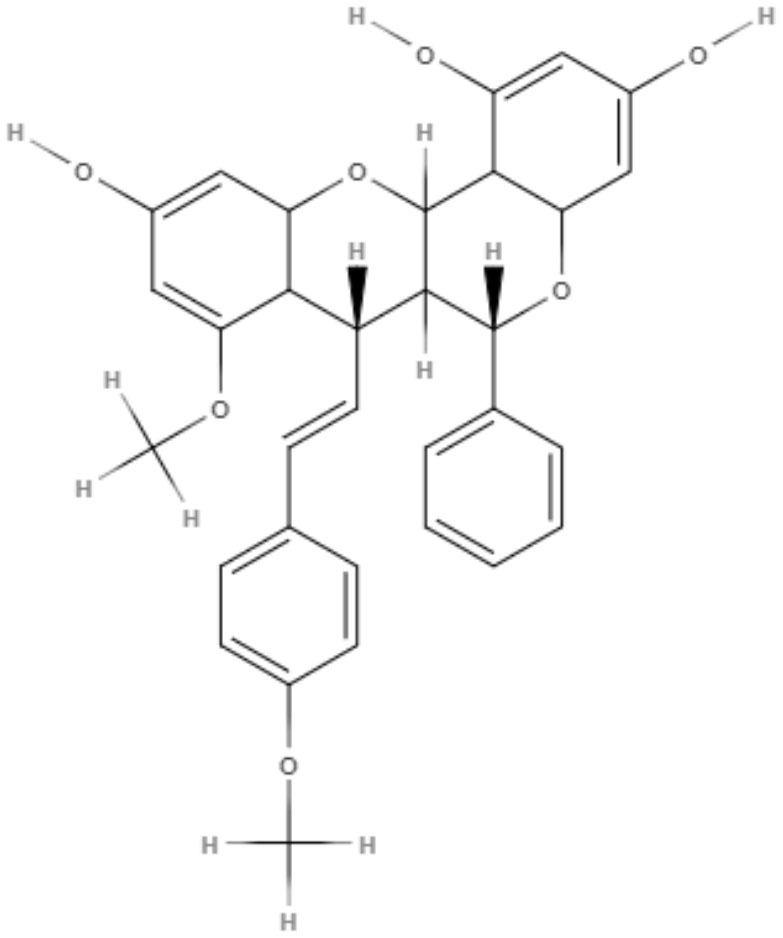	*L. braziliensis*	*In vitro*	**Promastigotes:** IC_50_: 7.05 μM	Rocha et al., 2018
			*L. amazonensis*	*In vitro*	**Promastigotes:** IC_50_: 9.16 μM **Intracellular amastigotes:** IC_50_: 2.2 μM	
				*In vivo*	ND	
		**Brachydin C** 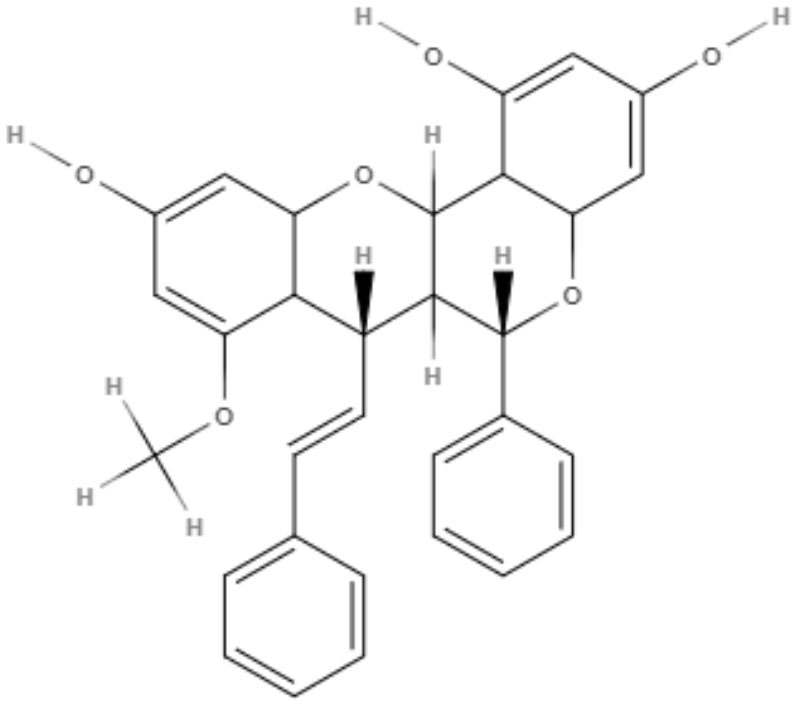	*L. brazilinesis*	*In vitro*	**Promastigotes:** IC_50_: 8.8 μM	
			*L. amazonensis*	*In vitro*	**Promastigotes:** IC_50_: 10.0 μM **Intracellular amastigotes:** IC_50_: 6.25 μM	

*ND, Not determined*.

a *5,7,3′,4′-tetrahydroxy-6,8-diprenylisoflavone–containing micelles*.

## Compounds From the Mevalonate Pathway

### Terpenoids

Terpenoids are natural compounds derived from C5 isoprene (or isoprenoid) units. The characteristic chemical structure of these compounds contains a skeleton with 5 carbons in a head-to-tail linkage. These compounds are classified as hemiterpenes (C5), monoterpenes (C10), sesquiterpenes (C15), diterpenes (C20), sesterterpenes (C25), triterpenes (C30), and tetraterpenes (C40). The diversity of terpenoids increases their biological activity spectrum, including several species of *Leishmania*, such as *L. major*, *L. donovani*, *L. infantum*, *L. amazonensis*, *L. braziliensis*, *L. Mexicana*, and *L. panamensis* (Yamamoto et al., 2015).

Artemisinin extracted from *Artemisia annua* and its derivatives were tested against promastigotes of *L. major*, demonstrating an IC_50_ value of 0.75 μM. Against intracellular amastigotes, artemisinin presented an IC_50_ value of 3 μM and was not toxic to the macrophages (Yang and Liew, 1993).

Artemisinin was also evaluated against *L. donovani*. The IC_50_ value was 160 μM against promastigotes and 22 μM against intracellular amastigotes. Artemisinin induced apoptosis, depolarization of the mitochondrial membrane potential and DNA fragmentation. *In vivo*, using BALB/c mice infected with *L. donovani*, artemisinin was administered at 5 and 10 mg/kg/day to reduce the parasite burden in the spleen (Sen et al., 2007, 2010).

The activity of the monocyclic sesquiterpene alcohol (-)-α-bisabolol, utilized in fragrances and extracted from *Matricaria chamomilla* L., was tested against intracellular amastigotes of *L. infantum* and *L. donovani*, presenting IC_50_ values of 56.9 and 39.4 μM, respectively. In addition to the *in vitro* investigation, this compound was also evaluated in a visceral leishmaniasis model, and it was determined to be non-toxic when administered orally, showed no mutagenic activity, was equally distributed across the tissues and reduced the parasite load in the spleen (71.6%) and in the liver (89.2%) (Corpas-López et al., 2015).

The effect of (-)-α-bisabolol was also analyzed in *L. tropica* and *L. major*. Against intracellular amastigotes, the compound demonstrated IC_50_ values of 25.2 μM for *L. tropica* and 33.7 μM for *L. major* with selectivity indices of 46 and 34, respectively. As a mechanism of action, (-)-α-bisabolol was able to increase ROS levels and decrease the mitochondrial membrane potential and phosphatidylserine exposure. In addition, in an ultrastructural analysis, the compound was capable of inducing mitochondrial disruption and chromatin condensation, indicating apoptosis (Corpas-López et al., 2016a). This mechanism of action was also observed in *L. amazonensis* and *L. infantum*. (-)-α-Bisabolol induced phosphatidylserine externalization and caused plasmatic membrane damage, both of which are apoptosis indicators. The compound also decreased ATP levels and disrupted the mitochondrial membrane potential (Hajaji et al., 2018), supporting the hypothesis that the possible mechanism of action for (-)-α-bisabolol is inducing programmed cell death.

(-)-α-Bisabolol was also analyzed *in vivo* against a murine model of cutaneous leishmaniasis using *L. tropica.* Topical formulation was capable of reducing the lesion size and parasite burden (Corpas-López et al., 2016b). (-)-α-Bisabolol was employed in a different approach in a preclinical trial for a canine leishmaniasis model using naturally infected dogs. The dogs were divided into two groups, treated with meglumine antimoniate (100 mg/kg/day) subcutaneously or (-)-α-bisabolol (30 mg/kg/day) orally. The sesquiterpene reduced the parasite load in analyzed tissues, increasing INF-γ levels without any toxicity. An evaluation of cytokines and antibodies suggests a Th1 response induced by the compound, indicating an anti-inflammatory pathway (Corpas-López et al., 2018).

Oleanolic acid and its isomer, ursolic acid (triterpenoids), were studied in promastigotes and intracellular amastigotes of *L. amazonensis*. Oleanolic acid did not demonstrate activity against promastigotes, but when its isomer was tested against promastigotes, an IC_50_ value of 6.2 μg/mL was obtained. Ursolic acid in promastigotes of *L. amazonensis* induced programmed cell death independent of caspase 3/7 but dependent on mitochondria. When the *in vivo* assay was performed for cutaneous leishmaniasis, the compound reduced the lesion size and parasite load (Yamamoto et al., 2015).

To evaluate the effects of ursolic acid in the *in vivo* model of visceral leishmaniasis, female golden hamsters were infected with *Leishmania infantum* promastigotes. Two different doses of ursolic acid were employed, that is, 1 and 2 mg/kg/day, injected intraperitoneally for 15 days. Both doses were able to reduce the parasite load in the liver (over 96% reduction) and spleen (over 92% reduction). Histopathological analysis of the spleen indicated fewer parasites compared to the infected untreated control, and both white and red pulp were conserved by ursolic acid treatment, which was corroborated by INF-γ, IL-4, and IL-10 gene expression and splenic cell proliferation. Ursolic acid did not affect toxicological parameters (Jesus et al., 2017).

Toxicity, high cost, resistance, and reduced bioavailability are current challenges facing leishmaniasis chemotherapy. Nanotechnology has been reported as a promising alternative (Shah and Gupta, 2019). To improve ursolic acid (UA) use for leishmaniasis, a UA-loaded N-octyl-chitosan surface-decorated nanostructured lipid carrier system (UA-NLC) was tested against wild-type *L. donovani* and sodium stibogluconate (SSG-R) and paromomycin (PMM-R) *L. donovani*-resistant axenic amastigotes. UA-NLC exhibited IC_50_ values of 0.12, 1.07, and 3.51 μM for wild-type parasites, SSG-R and PMM-R, respectively, which were lower than those of regular ursolic acid (IC_50_ = 1.82, 16.15, and 36 μM, respectively). Against intracellular amastigotes, UA-NLC exhibited an IC_50_ of 0.09, 2.87, and 5.57 μM, and UA demonstrated an IC_50_ of 1.08, 11.54, and 24.46 μM for wild type, SSG-R and PMM-R, respectively (Das et al., 2017).

In the cytotoxicity evaluation, UA showed a selectivity index of 227.78, and UA-NLC showed an SI of 9111.11, almost 40 times higher. The effect of UA-NLC and regular UA *in vivo*, both administered orally at 10 mg/kg, was assessed. Spleen amastigote suppression was evaluated for wild-type, SSG-R- and PMM-R-infected mice. UA exhibited a percentage of suppression of 68.14, 64.69, and 59.55%, respectively, while UA-NLC achieved better results with 98.75, 88.4, and 90.37%, respectively. All these results suggest that UA-NLC and nanodelivery systems are a promising approach for leishmaniasis chemotherapy (Das et al., 2017).

The Antarctic sponge *Dendrilla membranosa* and other similar species have been a rich source for chemical studies. *Dendrilla antarctica* sponges, as named by Shilling et al. (2020), exhibit a variety of diterpenes in their composition, such as aplysulphurin (1), tetrahydroaplysulphurin-1 (2), membranolide (3), and darwinolide (4). These compounds were evaluated against *Leishmania donovani*-infected macrophages and J774 cells for cytotoxicity assays. Compounds 1 and 2 had the most promising IC_50_ values, 3.1 and 3.5 μM, respectively, while 3 and 4 exhibited values above 10 μM. However, the selective index for compound 1 was less than 10, while for compounds 2, 3, and 4, it was higher than 30 (Shilling et al., 2020).

*Plumarella delicatissima* is an octocoral specimen of the Southern Ocean known as a source of bioactive terpenoids. Seven terpenoids, (keikipukalide A-E, pukalide aldehyde, and ineleganolide), were isolated from *Plumarella* sp. and analyzed against *L. donovani* amastigote. Pukalide aldehyde was the most promising compound, exhibiting an IC_50_ value of 1.9 μM. X-ray crystallography of all isolated terpenoids indicates the differences between *Pulmarella* sp. terpenoid chemical structure, which is an important first step in structure-activity studies that should be conducted in further research (Thomas et al., 2018).

Using a novel and different approach, Ogungbe and Setzer (2013) studied potential *in silico* targets for terpenoids in *Leishmania*. Unlike *in vitro* and *in vivo* approaches, *in silico* approaches are able to predict different aspects of a potential bioactive compound, such as its possible targets, best radicals or chemical structure, for better antileishmanial activity. Molecular docking analysis was performed for several known antiparasitic plant-derived terpenoids. Each terpenoid class studied was docked with each chosen Leishmania species (*L. infantum, L. Mexicana, L. major*, and *L. donovani*). The most promising targets were nicotinamidase (*L. infantum*), uridine diphosphate-glucose pyrophosphorylase, methionyl t-RNA synthetase and dihydroorotate dehydrogenase (*L. major*), glycerol-3-phosphate dehydrogenase (*L. mexicana*) for the matching terpenoid classes. Furthermore, these results may help to guide new research on the development of new potent antileishmanial terpenoids (Ogungbe and Setzer, 2013).

The terpenoids present in this section with a defined IC_50_ are summarized in the Table 8.

**Table 8:** Chemical structure and leishmanicidal activities of terpenoids.

**Class**	**Subclass**	**Compound name and chemical structure**	***Leishmania* species**	**Assay**	**Values**	**Reference**
Terpenoids	Sesquiterpene	**Artemisinin** 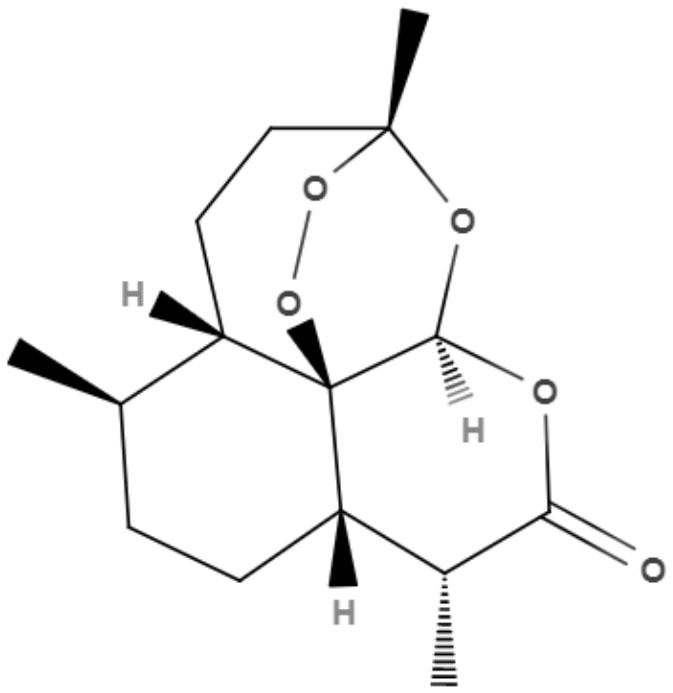	*L. major*	*In vitro*	**Promastigotes:** IC_50_: 0.75 μM **Intracellular amastigotes:** IC_50_: 3 μM	Yang and Liew, 1993
			*L. donovani*	*In vitro*	**Promastigotes:** IC_50_: 160.0 μM **Intracellular amastigotes:** IC_50_: 22.0 μM	Sen et al., 2007
				*In vivo*	ND	Sen et al., 2010
	Sesquiterpene	**(-)-α-Bisabolol** 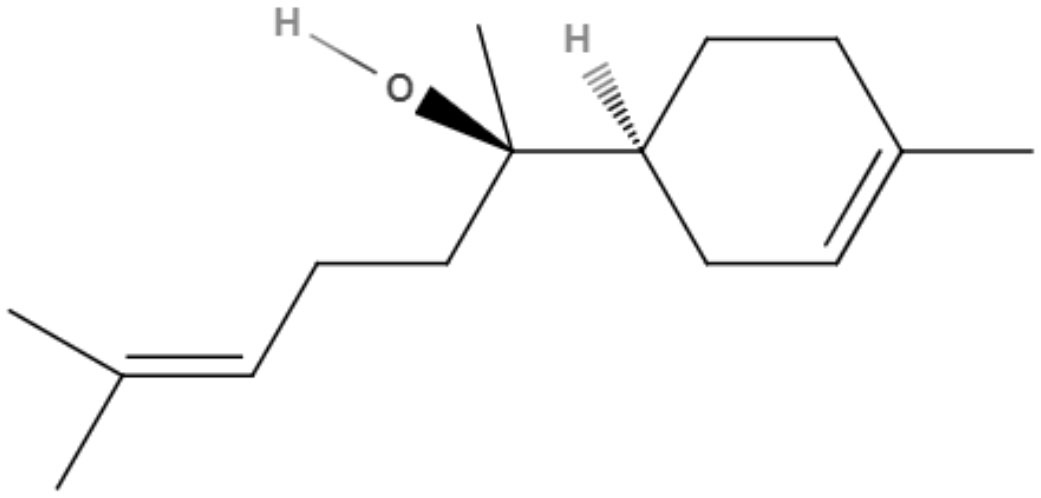	*L. donovani*	*In vitro*	**Intracellular amastigotes:** IC_50_: 39.4 μM	Corpas-López et al., 2015
			*L. infantum*	*In vitro*	**Intracellular amastigotes:** IC_50_: 56.9 μM	
				*In vivo*	ND	
			*L. major*	*In vitro*	**Intracellular amastigotes:** IC_50_: 33.7 μM	Corpas-López et al., 2016a
			*L. tropica*	*In vitro*	**Intracellular amastigotes:** IC_50_: 25.2 μM	
				*In vivo*	ND	Corpas-López et al., 2016b
Terpenoids	Triterpenoids	**Ursolic acid** 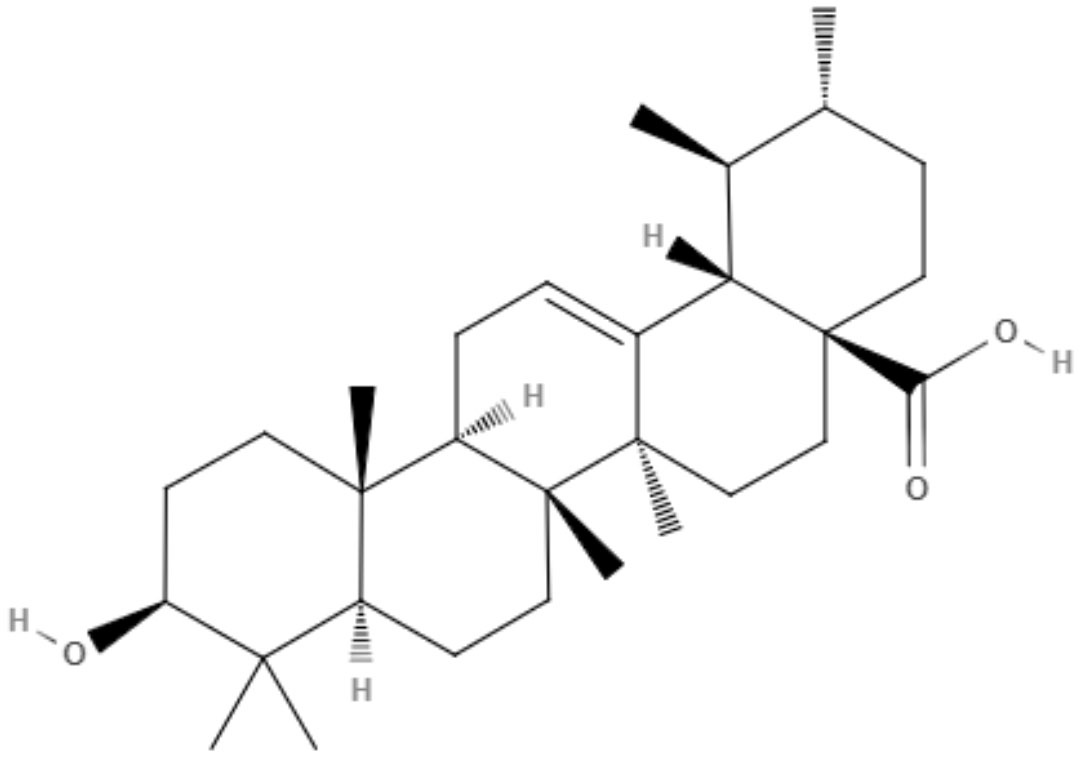	*L. amazonensis*	*In vitro*	**Promastigotes:** IC_50_: 6.2 μg/mL **Intracellular amastigotes:** ND	Yamamoto et al., 2015
				*In vivo*	ND	
			*L. infantum*	*In vivo*	ND	Jesus et al., 2017
			*L. donovani*	*In vitro*	**Axenic amastigotes:** IC_50_: 1.8 μM **Intracellular amastigotes:** IC_50_: 1.1 μM	Das et al., 2017
				*In vivo*	ND	
			*L. donovani - SSGR*	*In vitro*	**Axenic amastigotes:** IC_50_: 16.2 μM **Intracellular amastigotes:** IC_50_: 11.5 μM	
				*In vivo*	ND	
			*L. donovani - PMMR*	*In vitro*	**Axenic amastigotes:** IC_50_: 36 μM **Intracellular amastigotes:** IC_50_: 24.5 μM	
				*In vivo*	ND	
Terpenoids	Triterpenoids	**UA – NLC^a^**	*L. donovani*	*In vitro*	**Axenic amastigotes:** IC_50_: 0.12 μM **Intracellular amastigotes:** IC_50_: 0.09 μM	Das et al., 2017
				*In vivo*	ND	
			*L. donovani - SSGR*	*In vitro*	**Axenic amastigotes:** IC_50_: 1.1 μM **Intracellular amastigotes:** IC_50_: 2.9 μM	
				*In vivo*	ND	
			*L. donovani - PMMR*	*In vitro*	**Axenic amastigotes:** IC_50_: 3.5 μM **Intracellular amastigotes:** IC_50_: 5.6 μM	
				*In vivo*	ND	
Terpenoids	Diterpenes	**Aplysulphurin** 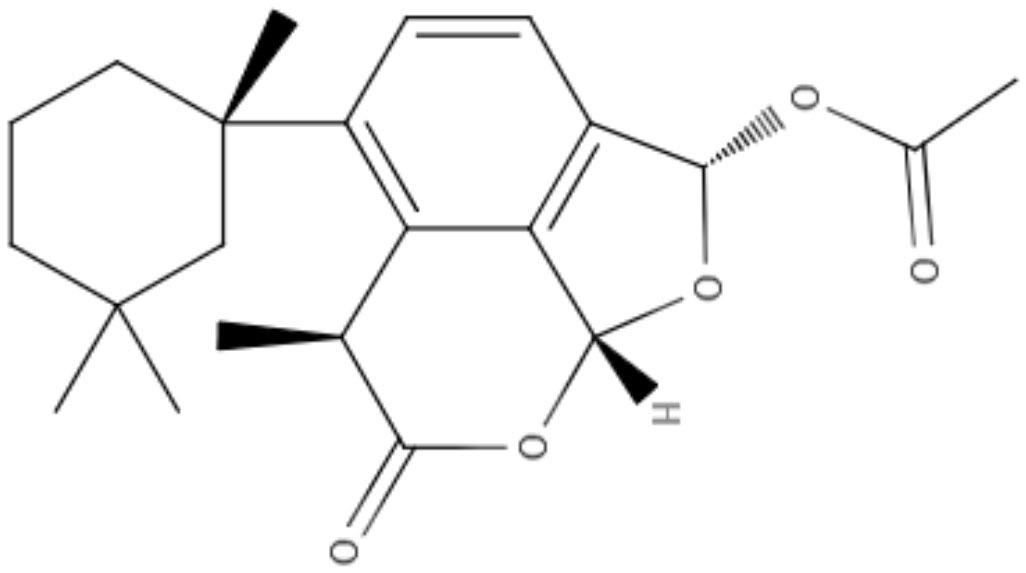	*L. donovani*	*In vitro*	**Intracellular amastigotes:** IC_50_: 3.1 μM	Shilling et al., 2020
		**Tetrahydroaplysulphurin-1** 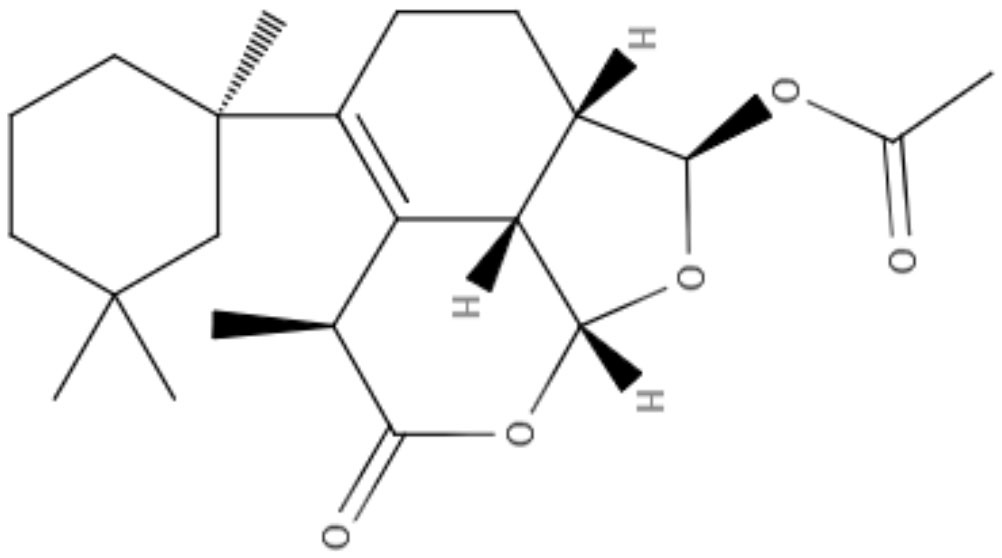			**Intracellular amastigotes:** IC_50_: 3.5 μM	
Terpenoids	Diterpenes	**Membranolide** 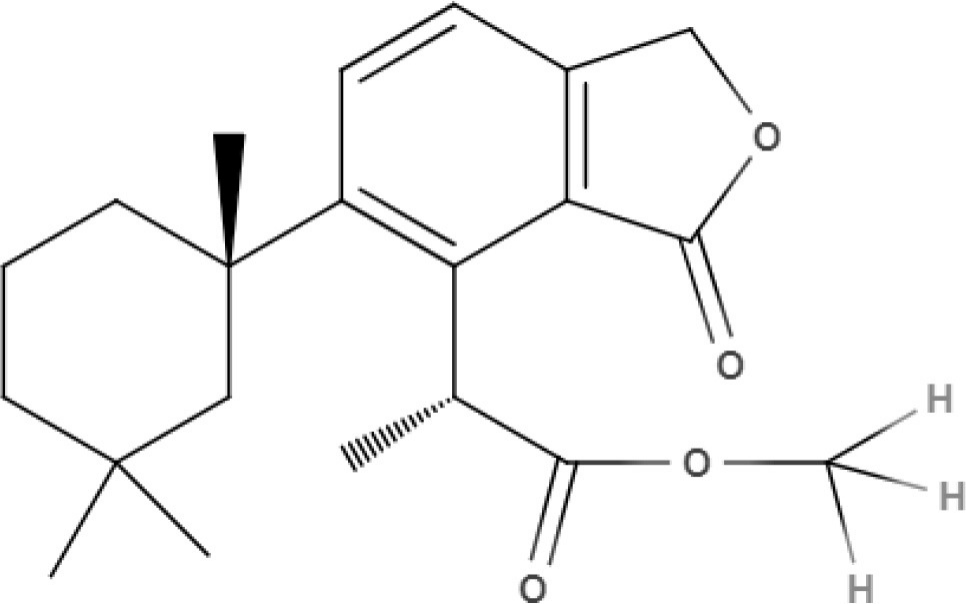	*L. donovani*	*In vitro*	**Intracellular amastigotes:** IC_50_: 9.7 μM	Shilling et al., 2020
		**Darwinolide** 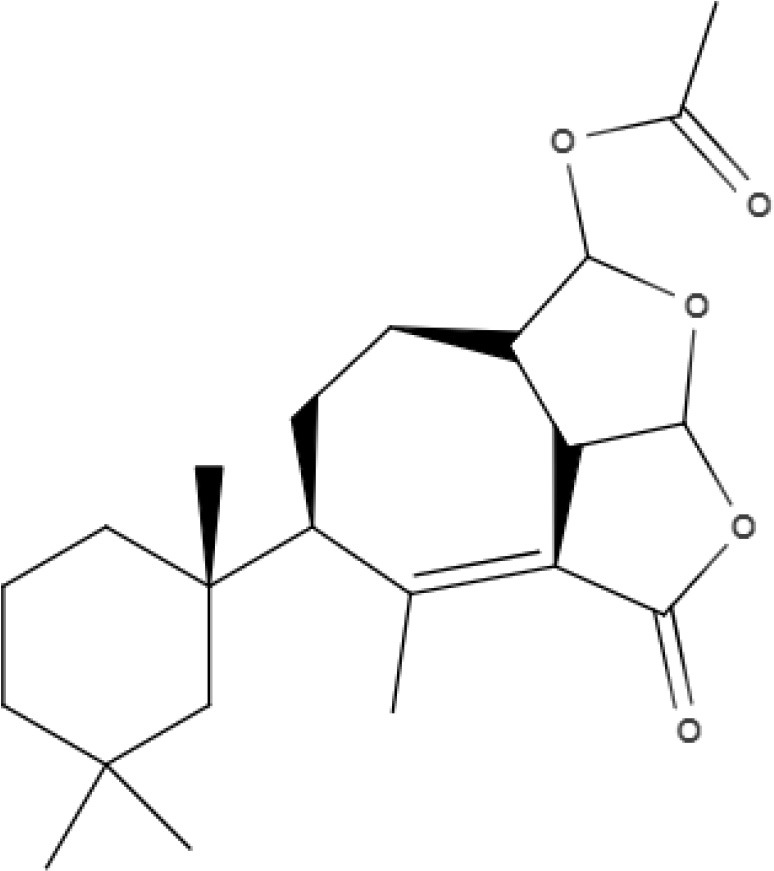			**Intracellular amastigotes:** 11.2 μM	
Terpenoids	Diterpenes	**Pukalide aldehyde** 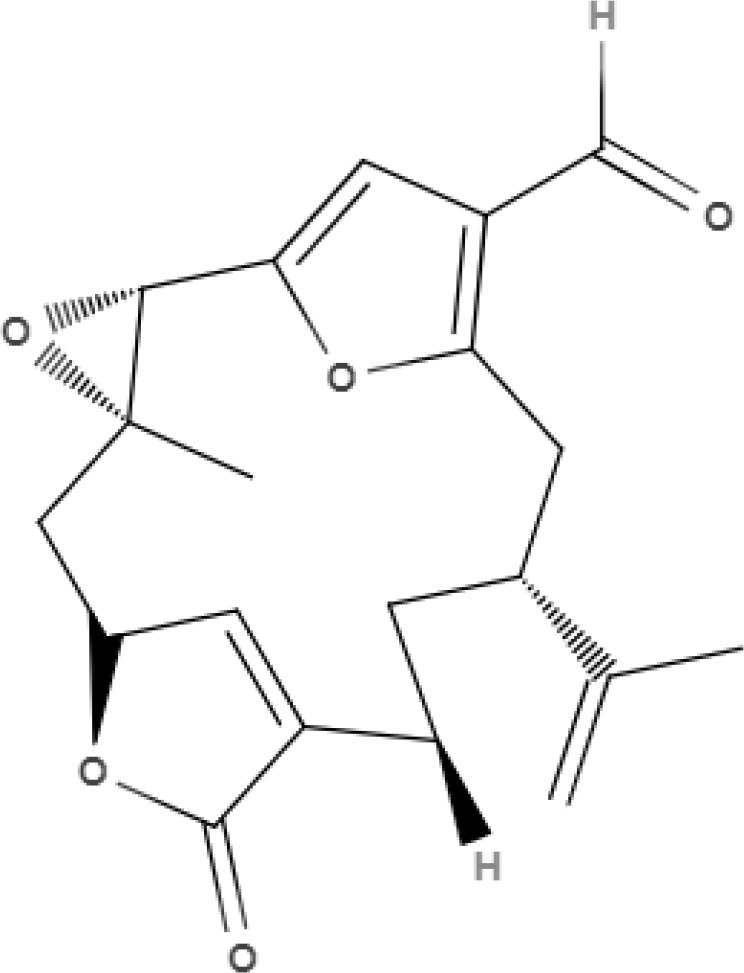	*L. donovani*	*In vitro*	**Intracellular amastigotes:** 1.9 μM	Thomas et al., 2018

a *Ursolic acid loaded N-octyl-chitosan surface decorated nanostructured lipid carrier system*.

*ND, Not demonstrated; SSGR, Sodium stibogluconate L. donovani resistant cells; PMMR, Paromomycim L. donovani resistant cells*.

## Discussion

Although leishmaniases are a group of diseases that have drug-based treatments, it remains a major challenger to research fields, since the currently available drug arsenal is reduced relative to the number of species that cause these diseases. Moreover, the chemotherapy that is utilized demands patient hospitalization and has been administered for many years, which can cause the occurrence of resistance and therapeutic failure. Therefore, research on new drugs for the treatment of these diseases is necessary.

The drug discovery process includes many steps to choose a new drug to treat a specific disease, and this process is expensive and time-consuming. Therefore, many strategies have been developed to optimize time and money.

There are 3 strategies more widely used in the drug discovery process:

(1) Fragment drug discovery based on molecules built for purpose. In this type of approach, automated techniques are used to trial compounds (i.e., high-throughput crystallography) to identify and optimize small molecules that bind to their target proteins with a variety of binding affinities (i.e., surface plasmon resonance). Hydrogen/deuterium exchange coupled with mass spectrometry (HDX-MS) and fragment libraries are techniques that play essential roles in this strategy. HDX-MS is a well-suited approach for investigating the alterations in protein conformation induced by small molecule ligand binding (Marciano et al., 2014). The fragment libraries identify smaller compounds, the “fragments,” which bind to different parts of a biological target. The primary rationale is that the identified hits provide access to a broader chemical space while screening a limited number of compounds (Schulz et al., 2011).
(2) Target direct screening is a biochemical approach based on repurposing or modifying existing molecules. In this approach, gene family platforms, compound libraries, computational models/informatics, structural biology and cellular and biochemical assays are extensively used to assess whether an existing molecule can be redirected as a treatment for the disease of interest (Lage et al., 2018). Some disadvantages of target direct screening, such as drug discovery and biochemical approach fragments for trypanosomatids, are the scarcity of fully validated drug targets and the need for additional screening to avoid off-target effects (Lage et al., 2018).
(3) Phenotypic drug discovery is a 'physiologically relevant' biological system or cellular signaling pathway that is directly investigated by chemical approaches to identify biologically active compounds. In contrast to the target-based strategies, these methods do not rely on knowledge of the identity of a specific drug target or a hypothesis about its role in disease. In this type of screening, advanced methodologies are able to answer many questions about a specific organic system, such as high content imaging, advanced informatics, advanced cellular assays, stem cells, SCORE, *in vivo* imaging, and the use of zebrafish models (Moffat et al., 2017).

The most widely employed methods for drug discovery against kinetoplastids are phenotypic, which is entirely justified, since the parasites have complex life cycles (the same parasite has different hosts and different forms in response to different temperatures and pH). The action of a compound on the parasite depends on its stage of life. As the drug discovery process can be developed at any stage of the parasite's life, the results may vary widely. Generally, in the case of trypanosomatids, the infective form of the parasite in the mammalian host is chosen to perform the drug discovery process; in the case of *Leishmania* sp., it is the intracellular amastigote (Lage et al., 2018).

In this review, the extensive models utilized for the drug discovery process employ dye-based indicators of parasite viability, such as resazourin, which is used for testing the drug susceptibilities of parasites. To evaluate the mechanism of action, methodologies are generally used to measure the levels of reactive oxygen species and the type of cell death triggered by the test compound.

Recent advances in automated microscopy have the capacity to increase throughput by replacing laborious manual microscopic observations with high-content imaging, and this technique is being successfully utilized in *in vitro* whole-organism screening against live kinetoplastid parasites (Siqueira-Neto et al., 2012). Bioinformatic tools that predict the potential of the compound as a drug, such as Linpinki's rules, along with other tools that predict the interaction of this compound with proteins (docking assays), provide robust data to assist in choosing cell-based assays to perform and enable the exclusion of certain compounds if they do not exhibit good results *in silico*.

Despite all the advantages of automated microscopy, it is important to note that there are some limitations that still need to be overcome; for example, the complex life cycles of trypanosomatids are challenging to reproduce in the laboratory, and effectiveness in one parasitic stage does not guarantee a strong *in vivo* effect (Lage et al., 2018).

Most drug discovery screenings for anti-kinetoplastid drugs are performed with synthetic compound libraries to search for active compounds, but such synthetic libraries are often limited in structural diversity and novelty (Fox et al., 2006). Natural products may be a solution to this problem, because as was extensively discussed throughout this review, in addition to having several biological activities, they have underexplored chemical entities that may be employed as templates for the synthesis of new drugs.

Research on natural products has been increasingly conducted over the years, as these products may represent an alternative treatment of leishmaniasis and may be considered potential chemotherapy agents, since they demonstrate promising results against *Leishmania* spp. All plant metabolic pathways described in this review showed strong activity against several species of *Leishmania in vitro*, and most importantly, some compounds showed activity *in vivo* using visceral and cutaneous leishmaniasis models of infection, which were often better than the results presented by the reference drugs.

Concerning the mechanism of action of these natural products, several compounds are capable of altering the mitochondrial membrane potential, causing an increase in intracellular ROS levels and a decrease in ATP concentration and leading to programmed cell death. Furthermore, using molecular docking approaches, some molecules were capable of interacting with important enzymes for the redox homeostasis of *Leishmania*, such as trypanothione reductase and trypanothione synthetase; however, it is important to demonstrate the inhibition of these activities by the selected molecules using recombinant enzymes. *In vivo*, some natural compounds were observed to reduce the parasite load and to act as immunomodulators.

In this scenario, it is possible that these compounds may be employed as a source of new treatments for leishmaniasis in the future, adding to the treatments already administered in the clinic.

## Author Contributions

All authors listed have made a substantial, direct and intellectual contribution to the work, and approved it for publication.

## Conflict of Interest

The authors declare that the research was conducted in the absence of any commercial or financial relationships that could be construed as a potential conflict of interest.

## Abbreviations

2HF: 2′-Hydroxyflavanone
ABC: ATP-binding cassette
AEPA: Aqueous extract of *Physalis angulata* root
AP-1: Activator protein 1
ATP: Adenosine triphosphate
BPQ: Buparvaquone
C: Carbon
CC_50_: Concentration that promotes 50% cytotoxicity
DHDE: Dehydrodieuginol
DNA: Deoxyribonucleic acid
DNDi: Drugs for Neglected Diseases Initiative
ED_50_: Median effective dose
EEPa: Ethanolic extract of *Physalis angulate*
EGCG: (–)-Epigallocatechin 3-O-gallate
FDA: Food and Drug Administration
GFP: Green fluorescent protein
HPLC: High-performance liquid chromatography
IC_50_: Half maximal inhibitory concentration
iNOS: Nitric oxide synthase inducible
LD_50_: Median lethal dose
MFI: Mean fluorescence intensity
mRNA: Messenger ribonucleic acid
MuEO: *Myracrodruon urundeuva* essential oil
NF-κB: Nuclear factor kappa-light-chain-enhancer of activated B cells
NO: Nitric oxide
PBS: Phosphate saline buffer
ROS: Reactive oxygen species
Sb^v^: Pentavalent antimony
SI: Selectivity index
∑FIC: Fractional inhibitory concentration sum
Th1: Helper T cell 1
Th2: Helper T cell 2
TIC: Total ion chromatogram
TrEO: *Tetradenia riparia* essential oil
TrROY: 6,7-Dehydroroyleanone
WHO: World Health Organization.
